# Ecological differentiation, speciation, and rarity: How do they match in *Tephroseris longifolia* agg. (Asteraceae)?

**DOI:** 10.1002/ece3.3770

**Published:** 2018-01-31

**Authors:** Monika Janišová, Katarína Skokanová, Tomáš Hlásny

**Affiliations:** ^1^ Institute of Botany, Plant Science and Biodiversity Center Slovak Academy of Sciences Bratislava Slovak Republic; ^2^ Faculty of Forestry and Wood Sciences Czech University of Life Sciences Prague Prague Czech Republic

**Keywords:** allopatry, biogeography, climate, co‐occurring species, distribution range, ecological niche, genome size, multivariate morphometrics

## Abstract

*Tephroseris longifolia* agg. is a complex group of outcrossing perennials distributed throughout Central Europe. Recent morphological study revealed six morphotypes corresponding to five previously distinguished subspecies, together with Alpine and Pannonian morphotypes of *T. longifolia* subsp. *longifolia*. The delimited morphotypes differ in relative DNA content, geographical range, and rarity. We compared ecological niches of the six morphotypes in order to assess the impact of ecological differentiation on the speciation processes within the *T. longifolia* agg. Further, we examined whether morphotypes with small range are more ecologically specialized than their widespread relatives. The distribution area of the aggregate includes the Alps, Apennines, Carpathians, and the Pannonian Basin. Ecological variables linked to climate, topography, soil, and vegetation were gathered from 135 circular plots recorded in 35 localities. Related variables were grouped to describe the partial ecological niches: climatic, topographic, pedological, biotic, and coenotic (based either on vascular plants or on bryophytes), each of them visualized as an envelope in the two‐dimensional nonmetric multidimensional scaling ordination space. Each partial ecological niche for a given morphotype was characterized by its position (location of the envelope centroid), breadth (surface of the envelope), and overlaps with envelopes of the other morphotypes. Mantel statistics based on Spearman correlation coefficients were used to quantify differentiation of morphotypes in ecological parameters represented by the partial ecological niches. The significant niche differentiation was confirmed for climatic, topographic, pedological, and vascular plant‐based coenotic niches. Ecological niche differentiation corresponded well to morphological and partially also to karyological differentiation. Narrowly distributed morphotypes occupied more specific habitats and had narrower ecological niches than their widespread relatives. Ecological differentiation could be considered an important driver in allopatric speciation within the *T. longifolia* agg. Our results demonstrate that quantification of ecological divergence is helpful in assessing evolutionary history of closely related taxa.

## 1 INTRODUCTION

European plant species ranges are shaped by current climate and range limits of a given species are maintained by its ecological niche (Normand et al., 2011; Wiens, 2011). However, also historical constraints strongly influence the current plant distribution. For instance, in the recent past temperature oscillations during Pleistocene glaciations (2.58–0.012 My ago) had dramatic impact on central European vegetation and caused extensive shifts, contractions, or expansions of plant distribution areas (Hewitt, 2011; Petit et al., 2003). Thus, current plant ranges are attributed to location of their refuge area(s) during the last glacial maxima (LGM) as well as postglacial migration conditioned by postglacial accessibility and the time factor (Normand et al., 2011; Wiens, 2011). Nevertheless, these natural processes might have also caused the fragmentation of previously continuous distribution ranges. In such cases, the interaction of the history and ecology often leads to allopatric speciation resulting in new taxa in different parts of the original distribution (Kreuzer, Tribsch, & Nyffeler, 2014; Thompson, Lavergne, Affre, Gaudeul, & Debussche, 2005).

Allopatric speciation conditioned by complete geographic isolation has two important consequences for plants: (1) gene flow is spatially limited and (2) different parts of the original distribution area differ in co‐occurring taxa due to distinct climatic, ecological, and historical conditions. Spatial isolation has a crucial role for homoploid speciation when reproduction isolation is usually lacking (e.g., Gross & Rieseberg, 2005; Martín‐Bravo, Valcárcel, Vargas, & Luceño, 2010; Watanabe, 1986). Additionally, distinct climate and ecological conditions allow gradual accumulation of genetic, morphological, ecological, and coenological differences in isolated populations and could result in establishing of new taxa. Thus, in spite of general tendency for species to retain similar ecological characteristic over evolutionary time scale (Kozak & Wiens, 2006), local speciation initiated by random genetic drift could be promoted by fixation of new gene combinations and selection of novel variants bringing adaptive advantage for plants in novel ecological conditions (Gross & Rieseberg, 2005; López‐Sepúlveda et al., 2013; Thompson et al., 2005).

Geographic isolation is the prevailing paradigm for the evolution of endemic taxa with narrow distribution (Thompson et al., 2005). In general, rarity could be caused by an interaction of historical (young or old species with narrow distribution), ecological (habitat specialist or/and poor competitors), and genetic (low genetic variation) factors (Baskin, Snyder, Walck, & Baskin, 1997; Stebbins, 1980; Walck, Baskin, & Baskin, 2001). In fact, narrow endemic species usually occur in habitats different from habitats of their widespread relatives (Kreuzer et al., 2014), but the question whether endemic taxa have a narrower range of ecological tolerance than their widespread relatives is still insufficiently studied (Fridley, Vandermast, Kuppinger, Manthey, & Peet, 2007; Thompson et al., 2005).

Ecological niche indicates the position of a species within an ecosystem, describing both the range of conditions necessary for persistence of the species, and its ecological role in the ecosystem (Polechová & Storch, 2008). A proper description of the ecological niche of particular species can be difficult because the number of niche dimensions is potentially infinite (Hutchinson, 1957), and the significant niche axes (and appropriate measures) may be rather hard to find. There are two basic approaches to measuring niches: (1) the classical method of determining niche breadth as the response of a species along environmental and resource gradients (e.g., Pannek, Ewald, & Diekmann, 2013) and (2) calculating niche breadth based on the co‐occurrence of other species (Fridley et al., 2007). The later approach is based on assumption that niches are delimited by the species that inhabit them (Levins & Lewontin, 1985) and environmental diversity is accurately reflected by the diversity of species that inhabit those environments (Fridley et al., 2007). Differences in abundance and distribution of individual taxa can be explained by different niche position and breadth. According to the niche position hypothesis (Hanski, Kouki, & Halkka, 1993), species utilizing common resources are common as well, whereas species specialized on rare habitats are also rare. In other words, generalists would have large geographical ranges and co‐occur with many different species, while specialists would likely be associated with only few species (Fridley et al., 2007). Accordingly, the niche breadth hypothesis (Brown, 1984) states that species able to exploit a wide range of resources are expected to occur over large areas and in high density.

To understand the context of ecological niche differentiation and ecological specialization, we focused on the group of *Tephroseris longifolia* agg. This aggregate represents an appropriate system for assessing ecological niche differentiation between widespread and endemic lineages as well as for testing the effects of niche divergence on speciation. The members of the aggregate have almost allopatric distribution through extensive part of Europe including Eastern Alps, Northern and Central Apennines, Western Carpathians, and Pannonian Basin. Interestingly, differences in vertical amplitude of occurrence and preferences of specific plant communities for particular morphotypes have been reported (Aeschimann, Lauber, Moser, & Theurillat, 2004; Hegedüšová, Škodová, Janišová, & Kochjarová, 2013; Janišová, Hegedüšová, Kráľ, & Škodová, 2012; Pignatti, Guarino, & La Rosa, in press). Thus, it seems that the habitats of morphotypes are climatically and ecologically differentiated, which suggests that the speciation involving adaptation to different environments and niche differentiation can be expected. Moreover, members of the aggregate are assumed for rather recent diversification due to weak reproduction barriers (Janišová, Škodová, & Hegedüšová, 2012; Šingliarová, Olšavská, Kochjarová, Labdíková, & Janišová, 2013) as well as only minute morphological differences (Olšavská et al., 2015). The chromosome number within the aggregate is conserved, but taxon‐specific relative DNA content was detected (Olšavská et al., 2015).

This study aims to relate a large set of quantitative ecological data to morphological and karyological patterns in *T. longifolia* agg. Along with the detailed characteristics of realized ecological niches (climatic, pedological, topographic, and biotic) of aggregate members, we investigated also the patterns of co‐occurrence of *T. longifolia* morphotypes with other species of vascular plants and bryophytes, representing their coenotic niches. We addressed the following questions: (1) What is ecological differentiation within the *T. longifolia* agg.? Does it correspond to morphological and karyological differentiation of the aggregate members? (2) Are morphotypes with small range more ecologically specialized than their widespread relatives?

## 2 METHODS

### 2.1 Object of study

#### 2.1.1 Morphology

Current morphological study of *T. longifolia* agg. (Olšavská et al., 2015) revealed six morphotypes roughly corresponding to previously distinguished subspecies (Greuter, 2006–2009): (1) Alpine morphotype of *T. longifolia* (Jacq.) Griseb. & Schenk subsp. *longifolia* (TLLA; Eastern Alps); (2) Pannonian morphotype of *T. l*. subsp. *longifolia* (TLLH; Pannonian Basin); (3) *T. l*. subsp. *moravica* Holub (TLM; Western Carpathians); (4) *T. l*. subsp. *pseudocrispa* (Fiori) Greuter (TLP; Julian Alps); (5) *T. l*. subsp. *gaudinii* (Gremli) Kerguélen (TLG; Eastern Alps); (6) *T. l*. subsp. *brachychaeta* (TLB; Northern and Central Apennines).

#### 2.1.2 Karyology

All members of *T. longifolia* agg. have the same chromosome number (2*n* = 48), and taxon‐specific relative DNA content was recovered for TLM + TLLH + TLLA, TLP, TLG, and TLB (Olšavská et al., 2015).

#### 2.1.3 Reproduction

Plants of *T. longifolia* agg. are outcrossing nonclonal herbaceous perennials. They are pollinated by insects and produce high number of achenes (up to about 2,000 per plant) with pappus allowing rather long dispersal (Janišová, Škodová, et al., 2012). Short‐persistence seed bank has been detected for *T. l*. subsp *moravica* (Janišová, Škodová, Hegedüšová, & Kochjarová, 2017). Flowering time differs little between the morphotypes.

#### 2.1.4 Habitats

*Tephroseris longifolia* agg. is distributed from lowlands to the subalpine regions in various types of habitats including open mesotrophic grasslands, light broad‐leaved forests, forest margins, and tall‐herb subalpine plant communities, but it is frequently present also in man‐influenced and disturbed secondary habitats.

#### 2.1.5 Protection

The narrow distribution and low number of populations of some aggregate members issued their conservation status. *Tephroseris longifolia* subsp. *moravica*, known just from nine localities, is treated as endangered taxon of national (Feráková, Maglocký, & Marhold, 2001; Grulich, 2012) as well as European importance (NATURA 2000, Directive 92/43/EEC, Annex II; Bilz, Kell, Maxted, & Lansdown, 2011). *Tephroseris longifolia* subsp. *longifolia* and *T. l*. subsp. *gaudinii* are considered as (regionally) endangered in Switzerland (Moser, Gygax, Bäumler, Wyler, & Palese, 2002), Austria (Niklfeld & Schratt‐Ehrendorfer, 1999), and Hungary (Király, 2007). Hungarian populations of *T. l*. subsp. *longifolia* are also under national legal protection (KvVM rendelet 23/2005).

### 2.2 Field sampling

**Figure 1:**
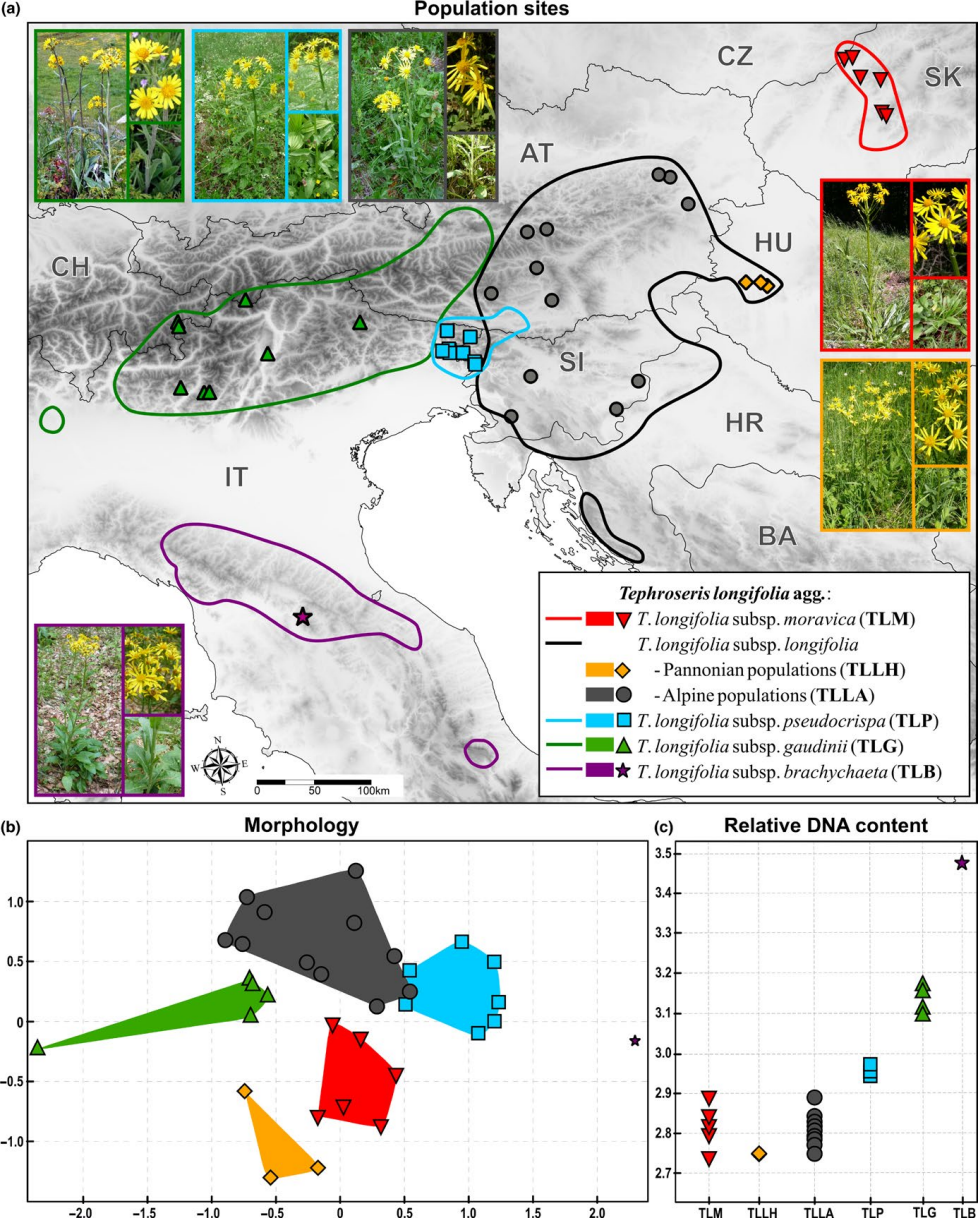
(a) Distribution map of the studied sites of the *Tephroseris longifolia* agg. General distribution of *T. longifolia* subspecies is marked by lines. Morphological (b) and DNA content (c) differentiation of morphotypes of *T. longifolia* agg. For details, see Olšavská et al. (2015)

Ecological data were recorded in 2011–2012 (May/June) for 35 population sites covering sites of the previous morphological and karyological study (Olšavská et al., 2015) within the whole distribution area of *T. longifolia* agg. (Figure 1, Table 1). Each investigated population site was assigned to one of the morphotypes of *T. longifolia* agg. (TLM, TLLH, TLLA, TLP, TLG, or TLB). Ecological variables linked to climate, topography, soil, and vegetation (Table 2) were gathered from two to eight circular plots recorded at each population site (altogether 135 plots; Table 1). Each plot was centered at randomly selected individual (vegetative or generative) of *T. longifolia* agg. within its typical habitat. Number of plots per population was determined by the heterogeneity of vegetation and habitat conditions at particular population site. From more heterogeneous population sites, more plot samples were obtained. Within each circular plot of 0.5 m^2^, the following data were recorded: geographical coordinates, altitude, aspect, inclination, % cover of co‐occurring species of rooting vascular plants and bryophytes, % covers of herb layer, moss layer, dead herb litter, fallen leaves of woody species, bare soil, and bare rock. Soil depth was measured using a metallic rod with a diameter of 4 mm (10 hits). At each plot, canopy light transmission was estimated using vertical hemispherical photographs taken 50 cm above the soil surface with a Nikon Coolpix 5400 digital camera (Nikon, Japan) equipped with a fisheye FC E9 objective. Canopy openness was estimated from the photographs using Gap Light Analyser 2.0 (Frazer, Canham, & Lertzman, 1999).

**Table 1:** Details on population sites including geographical coordinates, altitude, number of plots recorded, and number of plants examined for morphology (morf) or relative DNA content (kar) in previous study (Olšavská et al., 2015)

Population code	Population site	No. of plots	No. of plants morf/kar
*Tephroseris longifolia* subsp. *moravica* (TLM)			
CAV	Slovakia; Strážovské vrchy Mts., Čavoj village; 48°52′56.6″ N, 18°29′25.8″ E; 560–585 m	8	20/3
RAD	Slovakia; Tríbeč Mts., Radobica village; 48°34′27.2″ N, 18°29′54.6″ E; 480–560 m	7	20/3
HOD	Czech republic; Bíle Karpaty Mts., Hodňov village; 49°04′57.0″ N, 18°03′24.3″ E; 480–560 m	6	20/0
LYS	Slovakia; Biele Karpaty Mts., Vršatecké Podhradie village, Mt. Lysá; 49°04′17.0″ N, 18°08′41.4″ E; 740–780 m	4	12/3
OMS	Slovakia; Strážovské vrchy Mts., Omšenie village; 48°54′52.4″ N, 18°14′36.4″ E; 570–670 m	4	27/3
STR	Slovakia; Vtáčnik Mts., Mt. Stráž; 48°32′53.6″ N, 18°32′40.4″ E; 770–780 m	2	5/3
*T. longifolia* subsp*. longifolia—*Pannonian populations (TLLH)			
GOS	Hungaria; Veszprém county, Gösfa village, Mt. Göshegy; 46°58′08.0″ N, 16°52′13.0″ E; 210–230 m	4	16/3
HUS	Hungaria; Zala county, Huszonya village; 46°55′57.0″ N, 17°07′33.0″ E; 160–170 m	2	2/0
ZAL	Hungaria; Zala county, Zalabér village, Bagóvölgy valley; 46°58′05.0″ N, 17°02′49.0″ E; 210–220 m	2	10/3
*T. longifolia* subsp*. longifolia—*Alpine populations (TLLA)			
EBE	Austria; Lavantater Alpen Mts., Kärnten, Eberstein village; 46°47′51.0″ N, 14°33′07.0″ E; 570–622 m	2	20/3
FAL	Austria; Kärnten, Ebene Reichenau, Falkertsee; 46°51′45.4″ N, 13°49′36.8″ E; 1855–1890 m	2	21/3
FUR	Austria; Niederöstereich, Furth an der Triesting village; 47°57′35.2″ N, 15°57′49.8″ E; 413 m	0	0/3
HIR	Austria; Karawanken Mts., Ebriach, part Hirskeuche; 46°28′14.0″ N, 14°29′25.0″ E; 740–775 m	2	20/3
JAK	Slovenia; Polhov Gradec town, Mt. Sv. Jakob; 46°06′19.0″ N, 14°22′11.0″ E; 780–790 m	2	19/3
LOI	Austria; Karawanken Mts., Loiblpass saddle; 46°26′41.0″ N, 14°15′28.0″ E; 990–1005 m	2	21/3
LOR	Slovenia; Polhov Gradec town, Mt. Sv. Lorenz; 46°04′18.0″ N, 14°17′59.0″ E; 780–790 m	3	20/3
MAR	Austria; Ramsau bei Hainfeld village, Mariental valley; 47°59′04.9″ N, 15°49′49.4″ E; 510–525 m	2	4/3
PIT	Austria; Rosalien Gebirge Mts., Pitten village; 47°42′28.0″ N, 16°10′53.0″ E; 320–340 m	4	20/3
POD	Slovenia; Podsreda village; 46°01′34.0″ N, 15°35′11.0″ E; 470–480 m	4	9/2
TRD	Slovenia; Gabrje village, Mt. Trdinov vrh; 45°45′35.0″ N, 15°19′22.4″ E; 1135–1185 m	2	15/0
VRE	Slovenia; Senožeče village, Mt. Vremščica; 45°41′15.5″ N, 14°03′52.3″ E; 1004 m	2	15/2
*T. longifolia* subsp*. pseudocrispa* (TLP)			
GNI	Italy; Alpi Giulie, Gniviza village; 46°19′55.8″ N, 13°19′32.6″ E; 1066–1075 m	2	13/3
KAM	Italy; Alpi Giulie, Kamno village; 46°12′36.7″ N, 13°37′49.2″ E; 194–210 m	2	20/3
KOL	Italy; Alpi Giulie, Kolovrat saddle; 46°11′21.7″ N, 13°38′34.0″ E; 1062–1115 m	6	20/3
LAG	Italy; Alpi Giulie, Valle del Lago valley; 46°27′00.0″ N, 13°34′31.0″ E; 880–907 m; 16.5.2012	3	20/3
PON	Italy; Alpi Giulie, Pontebba village; 46°30′28.0″ N, 13°18′04.0″ E; 615–625 m; 16.5.2012	2	20/3
TAN	Italy; Alpi Giulie, Passo Tanemea saddle; 46°18′06.8″ N, 13°20′17.1″ E; 793–828 m	6	19/3
VOD	Slovenia; Alpi Giulie, Val Vodizza Valley, 46°18′47.5″ N, 13°15′04.6″ E; 839–883 m	6	0/0
ZAG	Slovenia; Alpi Giulie, Žaga village; 46°17′48.9″ N, 13°29′25.5″ E; 325–340 m	2	20/3
*T. longifolia* subsp*. gaudinii* (TLG)			
BAL	Italy; Monte Baldo Mts.; Mt. Altissimo; 45°48′12.6″ N, 10°53′26.3″ E; 1800–1850 m	6	20/3
BAZ	Italy; Breno town; Bazena saddle; 45°55′10.5″ N, 10°23′52.9″ E; 1869–1923 m	6	20/3
CHAS	Switzerland; Alp Trupchun; 46°35′35.5″ N, 10°04′52.0″ E; 2098 m	0	10/0
DOS	Italy; Darfo‐Boario; Dosso village; 45°57′52.1″ N, 10°06′59.7″ E; 1020–1050 m	5	0/3
FED	Italy; Val Federia Valley; 46°32′57″ N, 10°05′39″ E; 2030 m	0	0/3
FEN	Italy; Trento town; Mt. Fenner Joch; 46°17′29.1″ N, 11°09′20.1″ E; 1650–1680 m	4	0/3
GAV	Italy; Bagolino village; Siltar de Gaver valley; 45°55′19.0″ N, 10°27′34.7″ E; 1400–1563 m	5	19/3
MIS	Italy; Dolomity Mts., Auronzo Di Cadore; Missurina Lake; 46°35′24.0″ N, 12°15′30.0″ E; 1750–1770 m	4	13/3
*T. longifolia* subsp*. brachychaeta* (TLG)			
VAL	Italy; Secciata Mts., Mt. Vallombrosa; 43°44′22.2″ N, 11°34′29.2″ E; 1230–1325 m	8	15/5

**Table 2:** Environmental variables used to estimate six partial ecological niches of the studied morphotypes of *Tephroseris longifolia* agg

Environmental variable	Description and units
Climatic niche	
Altitude	(m a.s.l.)
AMT	Mean annual air temperature (°C)
ETR	Intra‐annual extreme temperature range (°C)
TX30	Number of extremely hot days with air temperature above 30°C (day)
SU	Number of summer days with air temperature above 25°C (day)
TNX0	Number of winter days with air temperature below 0°C (day)
TN10	Number of severe cold days with air temperature below −10°C (day)
PTGS	Precipitation total during growing season (April–September) (mm)
SDII	Simple daily precipitation intensity index, that is, total precipitation/total number of days with precipitation above 1 mm (mm/day)
CDD	Maximum number of consecutive dry days, that is, days with precipitation <1 mm (day)
RR1	Number of days with precipitation above 1 mm (day)
T_MIN	95% quantile of lowest daily air temperatures
GSS5	Starting day of growing season >5°C
Topographic niche	
Altitude	(m a.s.l.)
Slope	Inclination of microrelief (°)
Solar radiation	Potential direct solar irradiation (heat index) calculated from the slope and aspect data according to Parker (1988)
North	Northern aspect of a plot including aspect between 315° and 45° (binary variable)
East	Eastern aspect of a plot including aspect between 45° and 135° (binary variable)
South	Southern aspect of a plot including aspect between 135° and 225° (binary variable)
West	Western aspect of a plot including aspect between 225° and 315° (binary variable)
Pedological niche	
Soil depth	Depth of soil, measured by metallic rod with diameter of 4 mm, average of 10 measurements (cm)
pH‐KCl	Soil acidity estimated in KCl suspension
CEC	Effective cation exchange capacity of the soil
Na (%)	Percentage of natrium cations of the effective cation exchange capacity (%)
K (%)	Percentage of potassium cations of the effective cation exchange capacity (%)
Mg (%)	Percentage of magnesium cations of the effective cation exchange capacity (%)
Ca (%)	Percentage of calcium cations of the effective cation exchange capacity (%)
Ca:Mg ratio	Ratio of exchangeable calcium to exchangeable magnesium
P	Phosphorus (mg/kg of dry matter).
Humus	Soil humus content calculated from carbon content (%).
NH_4_	NH_4_ (mg/kg of dry matter).
NO_3_	NO_3_ (mg/kg of dry matter).
Biotic niche	
Cover of herb layer	Percentage cover of herb layer (%)
Cover of moss layer	Percentage cover of bryophytes (%)
Litter	Percentage cover of plant dead biomass (litter) in the herb layer (%)
Fallen leaves	Percentage cover of dead leaves of woody species on the plot surface (%)
Canopy Openness	Percentage of open sky seen from beneath a forest canopy calculated from hemispherical photography (%)
Vascular plants	Number of vascular plants in the plot except TLM
Number of bryophytes	Number of bryophyte species in the plot
Grasses	Proportion of grass and graminoid species in the total number of vascular plants (%)
Woody	Proportion of woody species in the total number of vascular plants (%)
Whittaker beta diversity	Whittaker multiplicative beta diversity according to Zelený (2009), average from 10 randomly selected plot pairs within a population site
Coenotic niche based on vascular plants	
Co‐occurring vascular plants	List of all co‐occurring vascular plant taxa recorded in plots within a locality (423 taxa altogether, taxa of *Tephroseris longifolia* agg. not included)
Coenotic niche based on bryophytes	
Co‐occurring bryophytes	List of all co‐occurring bryophyte taxa recorded in plots within a locality (55 taxa altogether)

### 2.3 Morphological data and relative DNA content

To quantify morphological and karyological differentiation of *T. longifolia* agg. members, we used previously published data from 33 populations (Olšavská et al., 2015). We analyzed 46 morphological characters on stem, leaves, and synflorescences for 527 individuals and determined relative nuclear DNA content for 98 individuals using DAPI flow cytometry (Figure 1, Table 1; for more detail, see Olšavská et al., 2015).

### 2.4 Vegetation data

The vegetation samples from circular plots were processed in the software JUICE (Tichý, 2002). Records of vascular plants and bryophytes were analyzed separately. Plant taxonomy and nomenclature of vascular plants follow Tutin et al. (1964–1993) and the one of bryophytes follows The Plant List (2013) (http://www.theplantlist.org/1.1/browse/B/). Altogether, 423 species of vascular plants and 55 species of bryophytes were recorded in the plots. For each plot, the number of vascular plant and bryophyte species was calculated to estimate species richness. Within the vascular plants, woody species and grasses including graminods (herbaceous plant with a grass‐like morphology) were distinguished and their proportion (%) was calculated. In order to characterize the heterogeneity of vegetation within each population site, Whittaker beta diversity (Zelený, 2009) was calculated in JUICE program for 10 randomly selected pairs of relevés. Several taxa determined only at the genus level were deleted prior to analysis. For determination of co‐occurring taxa of vascular plants and bryophytes with the highest fidelity to *T. longifolia* morphotypes, we calculated the *phi* coefficient after standardizing the size of relevé groups to the same size. Fisher's exact test (*p* < .01) was used to eliminate the fidelity value of species with a nonsignificant pattern of occurrence (Chytrý, Tichý, Holt, & Botta‐Dukát, 2002).

### 2.5 Soil data

Soil samples were taken from the uppermost mineral soil horizon from the depth of 5–10 cm. This zone is densely rooted by herbs, and a strong interaction between plants and soil factors is presumed. Three subsamples were taken for each circular plot (Table 1) and pooled together to form composite samples. The samples were air‐dried and measured for soil acidity in 1 mol/L KCl suspension (20 g soil plus 50 ml KCl), soil organic carbon content (standard Tyurin titrimetric method, Tyurin, 1951), and accessible phosphorus (Bray & Kurtz, 1945). Exchangeable potassium, calcium, magnesium, and sodium were estimated in NH_4_Cl extract using atomic absorption spectrometry. Nitrogen was estimated separately for ammonia (NH_4_) by SFS‐EN ISO 11732: 2005 and nitrate (NO_3_) by SFS‐EN ISO 13395:1996.

### 2.6 Climatic data

Climatic data for each population site were obtained from two observation‐based gridded datasets — the E‐OBS dataset, which contains daily data in ~25 × 25 km horizontal resolution (Haylock et al., 2008); and the CRU TS dataset v. 1.2 (Climatic Research Unit, University of East Anglia, UK; Mitchell, Carter, Jones, Hulme, & New, 2004), which contains monthly data in 1/6 × 1/6 degree horizontal resolution. The used climate data were averages for the period 1961–1990. External Drift Kriging‐based spatial interpolation was used to interpolate the climate data to site positions. This technique has been repeatedly proven well suited for interpolation of climate data (Goovaerts, 2000; Hudson & Wackernagel, 1994). Digital Elevation Model with spatial resolution 90 m (SRTM; Jarvis, Reuter, Nelson, & Guevara, 2008) was used as supportive variable. Additionally to mean annual air temperature and precipitation total during growing season, several climate indices providing specific information on sites’ climate were calculated (Table 2, Klein Tank & Können, 2003). Geostatistical software ISATIS v.8 (Geovariances, France) was used to interpolate the climate data.

### 2.7 Statistical analyses

To compare ecological niches of the *T. longifolia* morphotypes, the available environmental characteristics (Table 2) were grouped into six subsets, each representing a partial ecological niche: (1) climatic, (2) topographic, (3) pedological, (4) biotic, (5) coenotic based on vascular plants, and (6) coenotic based on bryophytes. Apart from climatic variables derived for the population sites, all variables were measured in circular plots and averaged for population sites. Each subset was analyzed by nonmetric multidimensional scaling (Canoco software, NMDS, two axes, Bray–Curtis or Euclidean distance measure selected so that the configuration “stress” is minimized; ter Braak & Šmilauer, 2012). The coordinates of population sites on two most important ordination axes were used to calculate distance matrices for population sites and to construct convex polygons (envelopes) for *T. longifolia* morphotypes using ArcMap GIS, v.10.3 (ESRI, USA). Partial ecological niches for each *T. longifolia* morphotype were characterized by their position (location of the envelope centroid) and breadth (envelope area). In addition, overlaps with envelopes of the other morphotypes were calculated. Similarly, the morphological differentiation was also quantified using the envelopes constructed from population coordinates in the two‐dimensional NMDS ordination space. For evaluation of differentiation in relative DNA content particular plants were analyzed in the unidimensional ordination space.

Mantel test was used to relate the distance matrices representing the partial ecological niches to (1) geographical distances derived from geographical coordinates of population sites, and (2) model explanatory matrix with the coded information on the taxonomical affiliation of individual *T. longifolia* populations to morphotypes (Appendix S1). The Mantel statistic based on Spearman correlation coefficient was used to quantify the level of segregation of the studied morphotypes. The morphological and karyological differentiation calculated from the multivariate morphometrical data and genome size, respectively, was then related to ecological (climatic, topographic, pedological, biotic, and coenotic) differentiation of the studied morphotypes. Partial Mantel test was used to control the effect of geographic distance, while Spearman correlation coefficients between the distance matrices and model matrix were calculated. Mantel test was calculated in the program XLSTAT (Addinsoft, 2009).

Pearson correlation coefficient was used to test the correlation between morphological characters or relative DNA content and climatic variables for particular populations of the *T. longifolia* agg.

## 3 RESULTS

### 3.1 Climatic niche

**Figure 2:**
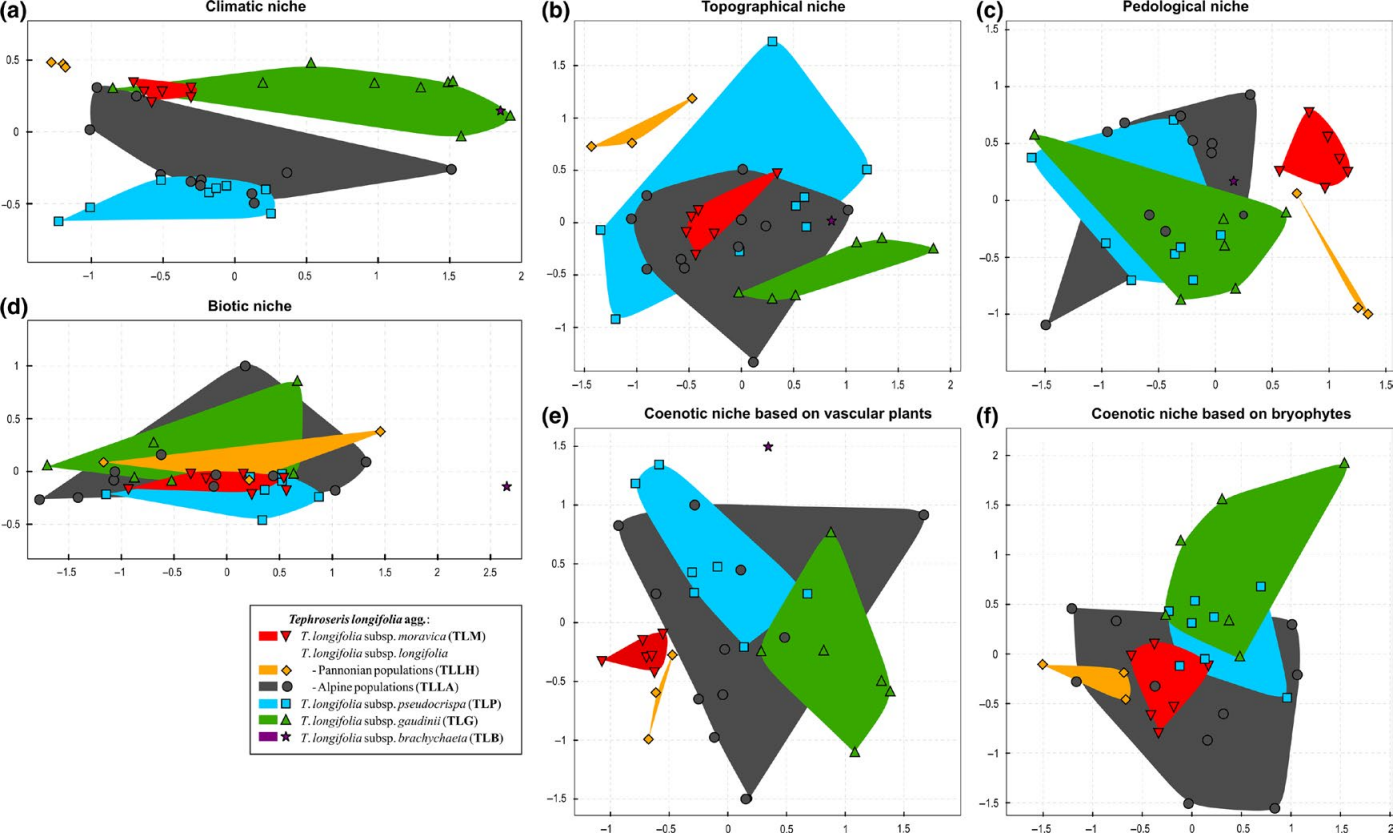
Differentiation of *Tephroseris longifolia* morphotypes in partial ecological niches depicted in a set of NMDS ordination plots: climatic (a), topographic (b), pedological, (c) biotic (d), vascular plant‐based (e), and bryophyte‐based coenotic (f) niches. For details, see Table 2

**Figure 3:**
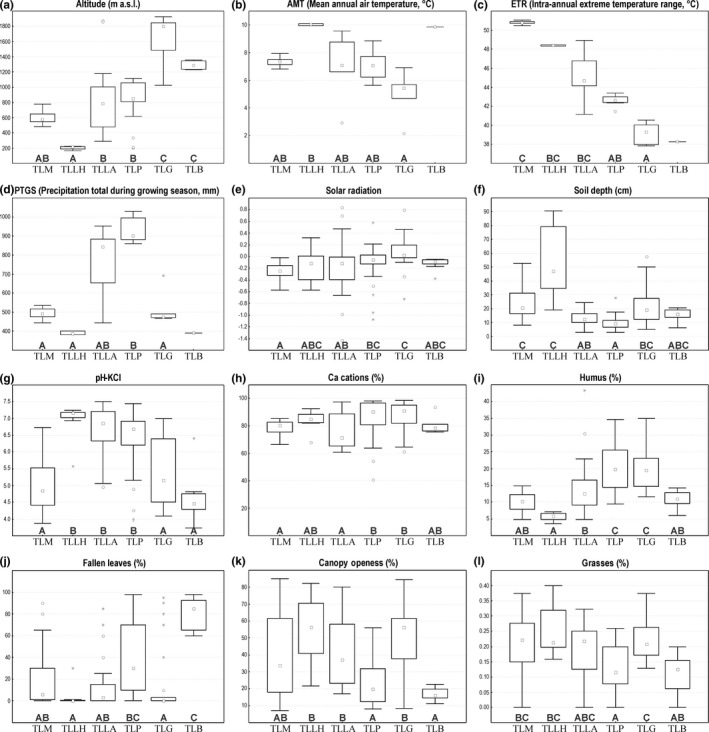
Boxplots depicting differences in selected climatic (a‐d), topographic (e), pedological (f–i), and biotic (j–l) characteristics

All morphotypes occupied sites with rather distinct climatic conditions (Figure 2a, Table 3). TLM and TLLH had much narrower climatic niches than the remaining morphotypes. The climatic niche of TLLH was isolated, and the one of TLM was overlapping mainly with TLG. TLM and TLLH were bound to warm continental climate (high number of warm and hot days, large intra‐annual extreme temperature range). TLG occupied regions with alpine climate (long winter, long duration of snow cover, little precipitation). For TLP, montane temperate climate was typical (200–1,200 m a.s.l., high precipitation totals; Figure 3a–e). The climate of TLLA population sites was most diverse; the populations in the SE Alps were similar to TLP, while the populations in NE Alps were similar to TLM.

**Table 3:** Morphological and karyological differentiation and differentiation of partial ecological niches of the *Tephroseris longifolia* morphotypes. To compare differences in morphology and partial ecological niches, convex envelopes in two‐dimensional ordination space and to compare differences in DNA content line segments in unidimensional ordination space were calculated

		Position (a.u.)					Breadth (a.u.)	Specific (%)	Sharing (%)				
		TLM	TLLH	TLLA	TLP	TLG			TLM	TLLH	TLLA	TLP	TLG
Morphology	TLM						0.32	100		0	0	0	0
	TLLH	0.76					0.15	100	0		0	0	0
	TLLA	1.11	1.62				0.84	99.5	0	0		0.50	0
	TLP	1.03	1.79	1.08			0.37	98.86	0	0	1.14		0
	TLG	1.38	1.22	1.20	2.05		0.26	100	0	0	0	0	
	TLB	2.21	2.89	2.54	1.47	3.47	NA	NA	NA	NA	NA	NA	NA
DNA content	TLM						0.75	47.53		0	52.47	0	0
	TLLH	0.33					0.01	0	100		100	0	0
	TLLA	0.23	0.10				1.26	68.13	31.37	0.50		0	0
	TLP	0.23	0.56	0.46			0.17	100	0	0	0		0
	TLG	0.83	1.16	1.06	0.60		0.57	100	0	0	0	0	
	TLB	1.78	2.10	2.00	1.55	0.94	NA	NA	NA	NA	NA	NA	NA
Climatic niche	TLM						0.05	23.35		0	0.85	0	75.79
	TLLH	0.76					0	100	0		0	0	0
	TLLA	0.68	1.40				0.98	93.59	0.04	0		6.31	0.05
	TLP	0.79	1.30	0.52			0.33	81.10	0	0	18.90		0
	TLG	1.27	2.02	0.82	1.32		0.73	95.13	4.80	0	0.07	0	
	TLB	2.38	3.14	1.86	2.30	1.12	NA	NA	NA	NA	NA	NA	NA
Topographic niche	TLM						0.25	0		0	97.66	100	0
	TLLH	1.11					0.09	100	0		0	0	0
	TLLA	0.37	1.47				2.29	33.86	55.15	0		60.20	5.94
	TLP	0.28	1.03	0.60			3.44	59.71	7.17	0	40.13		0
	TLG	1.24	2.31	0.97	1.29		0.43	68.49	0	0	31.51	0	
	TLB	1.06	2.03	0.93	1.01	0.46	NA	NA	NA	NA	NA	NA	NA
Pedologic niche	TLM						0.27	100		0	0	0	0
	TLLH	1.09					0.04	100	0		0	0	0
	TLLA	1.43	1.76				1.97	38.64	0	0		57.44	38.95
	TLP	1.62	1.91	0.19			1.55	9.83	0	0	72.72		61.80
	TLG	1.40	1.56	0.26	0.36		1.44	27.81	0	0	53.32	66.81	
	TLB	0.78	1.27	0.66	0.84	0.63	NA	NA	NA	NA	NA	NA	NA
Biotic niche	TLM						0.20	0		15.52	100	76.75	37.19
	TLLH	0.38					0.45	4.73	6.70		9.03	6.70	79.67
	TLLA	0.34	0.22				2.16	31.63	0.64	19.75		12.96	54.20
	TLP	0.21	0.38	0.45			0.54	48.28	28.69	5.55	51.72		10.46
	TLG	0.37	0.36	0.14	0.53		1.26	7.08	5.97	28.32	92.92	4.49	
	TLB	2.76	2.47	2.68	2.59	2.82	NA	NA	NA	NA	NA	NA	NA
Vascular plant‐based coenotic niche	TLM						0.11	100		0	0	0	0
	TLLH	0.37					0.02	100	0		0	0	0
	TLLA	1.10	1.16				3.45	57.23	0	0		23.04	20.54
	TLP	1.04	1.27	0.58			0.95	16.56	0	0	83.44		2.94
	TLG	1.58	1.49	0.66	1.23		1.14	37.54	0	0	62.46	2.47	
	TLB	2.06	2.30	1.36	1.03	1.79	NA	NA	NA	NA	NA	NA	NA
Bryophyte‐based coenotic niche	TLM						0.43	0		0	100	12.61	0
	TLLH	0.67					0.16	16.05	0		83.95	0	0
	TLLA	0.38	1.00				3.55	65.47	11.98	3.76		20.17	7.40
	TLP	0.74	1.37	0.66			0.90	0.24	5.96	0.00	79.53		48.89
	TLG	1.43	1.91	1.48	0.84		1.85	75.86	0.00	0.00	14.25	23.89	
	TLB	NA	NA	NA	NA	NA	NA	NA	NA	NA	NA	NA	NA

Position–distance of the envelope centroids or line segments (in arbitrary units, a.u.); breadth, area of the convex envelope; specific, % of area of particular morphotype without any overlap (a.u.); sharing, % of area of particular morphotype overlaping with other morphotype(s); NA, data not available.

### 3.2 Topographic niche

TLLH had a narrow and isolated topographic niche (low altitudes and mainly northern aspect). The niches of all other morphotypes were overlapping, TLLA + TLP + TLM with their centroid close to each other, TLG + TLLA with their centroids more distant from each other. The niches of TLM and TLG were rather narrow (reflecting a narrow altitudinal range of their distributions), but TLM preferred northern slopes, while TLG occurred mainly on slopes with a sun‐exposed aspect. TLLA and TLP had wide topographic niches suggesting that they are not bound to specific topographic conditions and reflecting their large altitudinal ranges (Figure 2b, Table 3).

### 3.3 Pedological niche

Isolated and narrow pedological niches were revealed for TLM and TLLH, while pedological niches of TLLA, TLP, and TLG were similar and fairly wide. Their centroids were close to each other and more than 2/3 of their pedological niches were overlapping (Figure 2c, Table 3). Compared to TLLA, TLP, and TLG, TLM and TLLH favored deeper soils poor in nitrogen and phosphorus. Populations of TLM occurred on more acidic soils (pH 3.4–6.7) than those of TLLH (pH 6.9–7.3). Within the aggregate, populations of TLP and TLG prevailed on calcareous soils with the highest effective cation exchange capacity (Figure 3f–i).

### 3.4 Biotic niche

All morphotypes occurred in habitats with similar biotic characteristics (with respect to cover of vegetation layers and litter, canopy openness, species richness, and beta diversity of co‐occurring vascular plants), which is reflected in large overlaps of their biotic niches and short distance among their centroids. TLM had the narrowest biotic niche (Figure 2d, Table 3). TLP occurred also in forest communities with closed canopy and high litter cover, which are rather species‐poor and host less grasses and graminoids (Figure 3j–i).

### 3.5 Coenotic niche based on vascular plants

**Figure 4:**
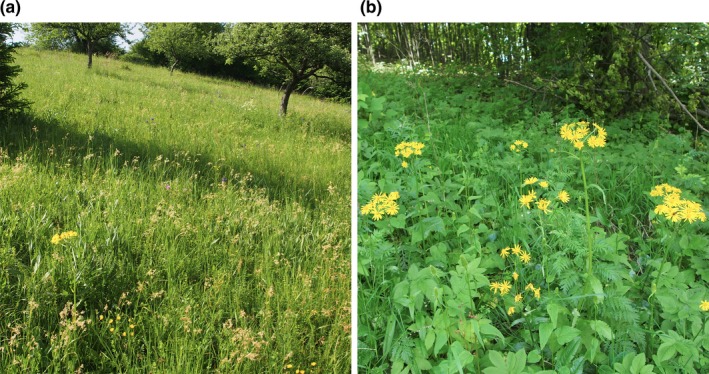
*Tephroseris longifolia* subsp. *moravica* is a rare endemic of the Western Carpathians occurring in semi‐natural meadows (a) and ecotones (b). Both pictures are from Čavoj, Slovakia. Photo: M. Janišová, 23 May 2014

TLM and TLLH had narrow and isolated niches (Figure 2e, Table 3). The plant communities with the occurrence of TLM were rich in grassland species (especially species of semi‐dry and mesic meadows, Figure 4). Vascular plants co‐occurring with TLLH consisted mainly of grassland species, species of thermophilous open forests and included also several taxa of man‐made habitats (Table 4). TLLA had the widest coenotic niche, substantially overlapping with the niche of TLP (with their centroids located close to each other). Both of them co‐occured mainly with typical forest species. The coenotic niche of TLG partly overlapped with the niche of TLLA, but their centroids were more removed due to high frequency of species typical of alpine meadows in the sites of TLG (Table 3).

**Table 4:** Vascular plants and bryophytes (B) with the highest fidelity (*phi* > 0.2, *phi* > 0.4 in bold) to the *Tephroseris longifolia* agg. morphotypes

***T. longifolia*** **subsp. ** ***moravica*** (TLM): *Arrhenatherum elatius, **Colchicum autumnale,** Crataegus monogyna, Crepis mollis, **Cruciata glabra,** Festuca rubra* agg*., Filipendula vulgaris, Fragaria moschata, **Knautia kitaibelii,** Lathyrus vernus, **Luzula luzuloides,** Lysimachia nummularia, Plagiomnium affine* (B)*, Poa trivialis, Potentilla erecta, **Primula veris,** Ranunculus acris, **Ranunculus auricomus** agg., Rhytidiadelphus triquetrus* (B), *Rosa canina* agg*., Rumex acetosa, Salvia pratensis, Symphytum tuberosum, Trisetum flavescens, Viola canina*
***T. longifolia*** **subsp. ** ***longifolia,*** Pannonian populations (TLLH): ***Ajuga reptans, Convallaria majalis, Eurhynchium hians*** **(B)** ***, Heracleum sphondylium, Knautia drymeia, Pimpinella saxifraga, Poa pratensis*** **agg** ***., Rhodobryum roseum*** **(B), ** ***Tragopogon pratensis*** **subsp** ***. orientalis, Vicia sepium, Viola hirta***
***T. longifolia*** **subsp. ** ***longifolia***, Alpine populations (TLLA): *Acer pseudoplatanus, Anemone nemorosa, Brachypodium pinnatum* agg*., Buphthalmum salicifolium, Campanula persicifolia, **Cyclamen purpurascens,** Euphorbia brittingeri, Geranium phaeum, Hedera helix, Helleborus odorus, Melica nutans, Mercurialis perennis, Picea abies, Primula vulgaris, Ranunculus bulbosus, Rhamnus cathartica*
***T. longifolia*** **subsp** ***. pseudocrispa*** (TLP): *Adoxa moschatellina, Allium ursinum, **Anemone trifolia, Angelica sylvestris,** Campanula rotundifolia, Cardaminopsis halleri, Equisetum arvense, Fraxinus excelsior, Galeopsis speciosa, Geum urbanum, Lamiastrum galeobdolon, Mentha arvensis, Mentha longifolia, Rubus idaeus, Silene dioica, Urtica dioica, Veratrum album, **Vincetoxicum hirundinaria***
***T. longifolia*** **subsp** ***. gaudinii*** (TLG): *Agrostis capillaris, **Alchemilla*** **species** ***,** Anthyllis vulneraria, Aposeris foetida, Biscutella laevigata, Brachythecium salebrosum* (B), *Carduus defloratus * **s.lat., ** ***Centaurea nigrescens, Crocus vernus*** **subsp** ***. albiflorus,** Deschampsia cespitosa, **Galium pumilum*** **agg** ***.,** Geranium sylvaticum, **Horminum pyrenaicum,** Juniperus communis, Lotus corniculatus, Luzula multiflora, Luzula sieberi, **Phyteuma orbiculare,** Pimpinella major, Poa alpina, **Polygonum viviparum,** Potentilla aurea, **Potentilla crantzii,** Primula elatior, Prunella vulgaris, **Ranunculus montanus*** **agg** ***., Ranunculus serpens,** Senecio doronicum, Sesleria albicans, **Soldanella alpina,** Stellaria graminea**, Thymus praecox,** Trifolium pratense, Trollius europaeus, **Viola biflora***
***T. longifolia*** **subsp** ***. brachychaeta*** (TLB): ***Euphorbia cyparissias, Fragaria vesca, Poa nemoralis, Rubus*** **species**

### 3.6 Coenotic niche based on bryophytes

TLLA had the broadest coenotic niche based on bryophytes completely involving the niche of TLM and partly overlapping also with the niches of TLLH, TLP, and TLG. Still, the majority of the TLLA niche (65%) remained nonoverlapping. The niche of TLG had least overlaps due to high number of bryophyte species occurring only at higher altitudes (Figure 2f, Table 3).

### 3.7 Correspondence of taxonomic and ecological differentiation

Morphology and DNA content have been proved as the most taxa‐specific characteristics (Table 5). Studied morphotypes also differed significantly in particular partial ecological niches, except the coenotic niche based on bryophytes. The major differences among the morphotypes were indicated in climatic, topographic, and vascular plant‐based coenotic niches. Foremost, in the case of climate, niche distance between morphotypes increased with geographic distance. After controlling the effect of geographical distance, the major differences among the morphotypes were found in topographic and vascular plant‐based coenotic niches.

**Table 5:** Segregation of *Tephroseris longifolia* morphotypes quantified by Mantel statistics (Spearman correlation coefficients). Distance matrices of populations for morphology, genetics, and partial ecological niches were related to a model matrix for taxonomical affiliation (the second column) as well as to the geographical distances among the population sites (the third column). The last column shows partial Mantel correlations between the distance matrices and a model matrix, while the effect of geographical distance was controlled

Distance matrix	Mantel statistics for distance matrices and		
	Taxonomical affiliation	Geographical distances	Taxonomical affiliation with controlled effect of geographical distance
Morphometrics	−0.456***	0.298***	−0.367***
Genome size	−0.582***	0.579***	−0.344***
Climatic niche	−0.337***	0.445***	−0.112**
Topographic niche	−0.288***	0.237***	−0.204***
Pedological niche	−0.156***	0.101*	−0.126***
Biotic niche	−0.090**	0.073^n.s.^	−0.078^n.s.^
Coenotic niche based on vascular plants	−0.286***	0.278***	−0.185***
Coenotic niche based on bryophytes	−0.083^n.s.^	0.157***	−0.016^n.s.^

***p < .001, **p < .01, *p < .05, n.s. not significant

At the population level, we found that several morphological characters are significantly correlated with climatic variables (Appendix S2). For example, populations grown in the areas with higher precipitation differed mainly in the shape of lower stem leaves (their leaves were shorter with its widest part closer to the leaf base). Populations occurring at higher altitudes with alpine climate (long winter, long duration of snow cover, little precipitation) tended to have densely hairy to arachnoid indument of involucral bracts, smaller diameter of capitulas, wider involucrum, and shorter pedicels in inflorescences. Similarly, we found correlation of relative DNA content and environmental variables: Populations with larger genomes tended to occur at higher altitudes with alpine climate (Appendix S2).

## 4 DISCUSSION

### 4.1 Ecological niche differentiation within the *T. longifolia* agg. corresponded well to morphological and partially also to karyological differentiation

Studies focusing on ecological niche of intraspecific taxa are very few (e.g., Jaime, Alcántara, Bastida, & Rey, 2015; Kreuzer et al., 2014) and data published so far suggest that substantial ecological niche differentiation occurs also on rather low taxonomic levels. The studied morphotypes of the *T. longifolia* agg. differed in position (ecological optimum) and breadth (ecological amplitude) of their realized ecological niches. The significant niche differentiation was confirmed for climatic, topographic, pedological, and vascular plant‐based coenotic niches. According to the recent studies (Blanco‐Pastor & Vargas, 2013; Jaime et al., 2015; Kreuzer et al., 2014; Normand et al., 2011; Pannek et al., 2013), soil parameters are expected to be among the major predictors of plant distribution at smaller spatial scales, while climate and altitude become increasingly important toward large scales. The indicated morphotype differences in co‐occurring vascular plant species are also important as they support our suggestion on gradual niche divergence within the geographically and climatically distinct areas due to the adaptation to local coenotic conditions. We admit that this coenotic adaptations might have been the first step of the speciation process and that the resulting niche differentiation has been subsequently manifested in morphological differentiation. Lacking differences in bryophyte‐based coenotic niches suggest the absence of ecological adaptations in the studied morphotypes induced by vascular plants–bryophytes competition. These two taxonomic groups are known to respond differently to most environmental factors (Herben, 1987; Virtanen & Crawley, 2010). With regard to the above‐mentioned findings, our results are well in accordance with the niche‐assembly theory rather that with the neutral theory of species coexistence (Wiens, 2011).

In our study, ecological data were highly correlated with morphological ones: (1) The distinct position of *T. l*. subsp. *moravica* (TLM) and Pannonian morphotype of *T. l*. subsp. *longifolia* (TLLH) was supported mainly by isolation of their pedological and vascular plant‐based coenotic niches. Pannonian morphotype of *T. l*. subsp. *longifolia* (TLLH) had also clearly isolated topographic and climatic niche. (2) Alpine morphotype of *T. l*. subsp. *longifolia* (TLLA) had the widest partial niches (except the topographic one), which usually occupied intermediate position among the niches of remaining *T. longifolia* morphotypes. (3) The clear separation of coenotic and biotic niches of *T. l*. subsp. *brachychaeta* (TLB) corresponded well to its distinct morphology (although for TLB, only a single population was investigated and these results should be verified after including more TLB populations).

Interestingly, the ecological pattern within the *T. longifolia* agg. does not fully correspond to variation in relative DNA content. The importance of environmental conditions and/or geographical distribution to the variation of genome size has been emphasized in several studies (e.g., Dušková et al., 2010; Pecinka, Suchánková, Lysak, Trávníček, & Doležel, 2006). In general, the variation in DNA content within the *T. longifolia* agg. is correlated with environmental variables such as altitude and geographic location. Also, at the population level, we found a significant trend for higher genome sizes in alpine conditions. However, in our study, *T. l*. subsp. *moravica* (TLM), Pannonian and Alpine morphotypes of *T. l*. subsp. *longifolia* (TLLA and TLLH) were well ecologically differentiated in spite of overlapping values of their genome size. This fact could suggest that genome size in this case is not strongly influenced by ecological conditions of populations but is rather taxon‐specific and could indicate common evolution.

### 4.2 Ecological differentiation played an important role in evolution of *T. longifolia* morphotypes

Our study confirmed that generally all studied populations of *T. longifolia* agg. occur in very similar habitats including mesotrophic grasslands, tall‐herb subalpine plant communities, open forests and forest margins, usually with deeper soils of intermediate pH values. The habitats of *T. longifolia* noticeable differ from the habitats preferred by other members of the *Tephroseris* genus occurring in Central Europe: *Tephroseris crispa* (Jacq.) Rchb. and *Tephroseris helenitis* (L.) B. Nord occurring mainly in neutral to acidophilus wet meadows and fens, and *Tephroseris integrifolia* (L.) Holub growing mainly in nutrient‐poor dry and semi‐dry calcareous grassland and open forest communities (Hegedüšová et al., 2013; Meindl, 2011; Pflugbeil, 2012). This could imply that phylogenetic niche conservatism limits the ecological adaptation of the *T. longifolia* agg. members (cf. Kozak & Wiens, 2006). However, to confirm it more accurate data are needed also for the other *Teproseris* taxa mentioned above.

Over the last decades, several studies have documented contrasting impact of niche conservatism and ecological adaptation during the evolution of plants (e.g., Prinzing, et al. 2001; Wasof et al., 2015; Kolanowska, Grochocka, & Konowalik, 2017) and animals (e.g., Dormann, Gruber, Winter, & Herrmann, 2010; Kozak & Wiens, 2006; Rissler & Apodaca, 2007). However, the importance of niche differentiation in the evolution of a particular plant group remains still poorly recognized. In our study, thanks to very precise and complex data collected mostly directly in the field, we are able to look deeply in ecological differentiation among closely related taxa of the *T. longifolia* agg. Within the aggregate, we found clear differentiation of particular morphotypes in climate, soil, and vascular plant co‐occurrence patterns. This fact could indicate that taxonomic differentiation within the aggregate had been accompanied by adaptation to new climatic conditions that may arise after colonization of distinct areas. Because experimental hybridizations showed no reproductive isolation among the morphotypes (Janišová, Škodová, et al., 2012; Šingliarová et al., 2013), the divergence within the aggregate could be attributed mainly to geographical isolation. Geographic isolation resulting from range shifts during Pleistocene in central Europe could lead to selection and local adaptation to different environments and promote niche differentiation (Jaime et al., 2015; Kreuzer et al., 2014; Thompson et al., 2005). Speciation of outcrossing plants is driven by fine adaptation of ecotypes through extensive recombination and heterozygosity (Blanco‐Pastor & Vargas, 2013; Polechová & Storch, 2008). Taxa can adapt to different resources by an independent process of evolutionary optimization, as plant speciation is driven by selection (and subsequent fixation) of novel variants bringing adaptive advantage for plants in novel ecological conditions (Gross & Rieseberg, 2005; Thompson, 1999; Thompson et al., 2005; López‐Sepúlveda et al., 2013; Polechová & Storch, 2008). In case of *T. longifolia* agg., some morphological traces point to adaptation promoted by different ecological and climatic conditions: Plants of *T. l*. subsp. *gaudinii* (TLG) occurring at the highest altitudes with the highest solar radiation (Figure 3e) have most hairy and condense synflorescences, while plants of *T. l*. subsp. *moravica* (TLM) and Pannonian morphotype of *T. l*. subsp. *longifolia* (TLLH) growing at lowest altitudes (Figure 3a) have predominantly grabrescent and lax synflorescence. Plants of *T. l*. subsp. *pseudocrispa* (TLP) with distribution constraint to an area with the highest precipitation (Figure 3d) have the widest leave laminas within the aggregate (Appendix S2; Olšavská et al., 2015).

Unfortunately, we were not able to distinguish whether *T. l*. subsp. *moravica* (TLM) and Pannonian morphotype of *T. l*. subsp. *longifolia* (TLLH) occupied similar ecological niches due to niche conservatism or if their morphological affinities, similar DNA contents and ecology resulted from a parallel evolution. In order to understand the past processes, further genetic analyses of the *T. longifolia* agg. are required.

### 4.3 Narrowly distributed morphotypes of *T. longifolia* agg. occupied more specific habitats and had narrower ecological niches than their widespread relatives

Narrowly distributed endemics *T. l*. subsp. *moravica* (TLM) and Pannonian morphotype of *T. l*. subsp. *longifolia* (TLLH) showed the highest level of ecological specialization and had the narrowest partial ecological niches (Figure 2, Table 3). Particularly edaphic, climatic, and coenotic variables were responsible for their niche differentiation within the aggregate. On the other hand, in spite of its narrow geographical distribution, most partial niches of *T. l*. subsp. *pseudocrispa* (TLP) were wide resembling rather widespread *T. l. *subsp. *gaudinii* (TLG) and Alpine morphotype of *T. l*. subsp. *longifolia* (TLLA). In addition, more abundant populations were observed for *T. l*. subsp. *pseudocrispa* (TLP) in comparison with *T. l*. subsp. *moravica* (TLM) and Pannonian morphotype of *T. l*. subsp. *longifolia* (TLLH) (M. Janišová, personal observation).

In the recent decades, several studies focused on biological and ecological differentiation between narrow endemic and widespread congeneric plants. Comparative studies documented that narrow endemics are very often associated with stressful or unusual conditions with disturbances and low competition (e.g., rocky habitats, steeper slopes, extreme cold or dry climates, flooding, hight fire frequency; Médail & Verlaque, 1997; Lavergne, Thompson, Garnier, & Debussche, 2004; Thompson et al., 2005; Fridley et al., 2007). Thus, theoretically a lower competitive ability has been expected for narrow endemic species (Baskin et al., 1997). However, the results of experimental studies are ambiguous, indicating that narrow endemics, in comparison with widespread relatives, either differ (Lavergne et al., 2004; Thompson et al., 2005; Walck et al., 2001) or not differ, or even are more successful in germination rate, seedling survival, and/or competition ability (Baskauf & Eickmeier, 1994; Matesanz, Valladares, & Escudero, 2009; Imbert, Youssef, Carbonell, & Baumel, 2011). Similarly, the expectation of low genetic variation of narrow endemic species (Stebbins, 1980; Walck et al., 2001) has not been universally proved (Matesanz et al., 2009). On the other hand, the biological traits such as understorey grow habit and seed dispersal importantly influenced the breadth of ecological niches (Blanco‐Pastor & Vargas, 2013; Fridley et al., 2007; Lavergne et al., 2004; Thompson et al., 2005; Walck et al., 2001; ).

The members of the *T. longifolia* agg. are very similar in their biology and do not differ considerably in any biological traits attributed to reproduction or plant dispersal. Ongoing studies also indicate that seed production and germination of narrowly distributed *T. l*. subsp. *moravica* (TLM) and Pannonian morphotype of *T. l*. subsp. *longifolia* (TLLH) are not reduced (Janišová, Škodová, et al., 2012; and unpublished results). Thus, these factors cannot explain the rarity of these morphotypes, but further studies have to be conducted to expose competitive ability and genetic variation of particular members of the aggregate.

Further, it has been assumed that narrow endemic species exploit narrower range of environmental conditions in comparison with widespread relatives (Imbert et al., 2011; Lavergne et al., 2004; Pannek et al., 2013; Thompson et al., 2005) and co‐occur with fewer mainly ecologically similar species (Fridley et al., 2007; Kreuzer et al., 2014). For of *T. l*. subsp. *moravica* (TLM) and Pannonian morphotype of *T. l*. subsp. *longifolia* (TLLH), narrower ranges of important enviromental variables have been indicated, particularly among the pedological and climatic factors, which could restrict their distribution. However, both these morphotypes grow in species‐rich plant communities and co‐occur with high number of plant species with distinct functional traits.

The number and size of the existing population sites for each of the *T. longifolia* agg. member depend to some extent on human activities (forest and grasslad management and especially mowing and grazing). Mainly in case of *T. l*. subsp. *moravica* (TLM) and Pannonian morphotype of *T. l. *subsp. *longifolia* (TLLH), land‐use changes connected with socio‐economical changes in the eastern part of Central Europe lead to decrease of site number (Olšavská et al., 2015). We would like to stress that the population sites of *T. longifolia* deserve protection and regular management as habitat stability may be crucial for persistence of its endemic morphotypes (c.f. Thompson et al., 2005; Janišová, Hegedüšová, et al., 2012; Janišová et al., 2017).

## CONFLICT OF INTEREST

None declared.

## AUTHOR CONTRIBUTIONS

MJ conceived the ideas and organized data collection; all authors analyzed the data and contributed to the writing.
