# Supplementary material for: Ecological differentiation, speciation, and rarity: How do they match in Tephroseris longifolia agg. (Asteraceae)?
Source: Ecol Evol. 2018 Jan 31;8(5):2453–70. doi: 10.1002/ece3.3770 (PMC5838062; doi:10.1002/ece3.3770)
Supplement: Supplementary file 1 [file ECE3-8-2453-s001.pdf]

Supporting information to the manuscript of Janišová et al., Ecological differetiation, speciation and rarity – how do they match in *Tephroseris longifolia* agg. (Asteraceae)? submitted to Diversity and Distributions

Appendix S1 Distance matrices for calculation of Mantel correlation coefficients: a) geographic distances, b) taxonomical affiliation, c) morphometrics, d) genome size, e) climatic niche, f) topographic niche, g) pedological niche, h) biotic niche, i) coenotic niche based on vascular plants, j) coenotic niche based on bryophytes.

a) Geographic distances

Locality	BAL	BAZ	CAV	DOS	EBE	FAL	FEN	FUR	GAV	GNI	GOS	HIR	HOD	HUS	CHAS	FED	JAK	KAM	KOL	LAG	LOI	LOR	LYS	MAR	MIS	OMS	PIT	POD	PON	RAD	STR	TAN	TRD	VAL	VOD	VRE	ZAG	ZAL
BAL	0.00	0.51	8.20	0.79	3.79	2.57	0.56	5.51	0.45	2.49	6.09	3.66	7.88	6.34	1.13	1.09	3.49	2.77	2.78	2.76	3.43	3.42	7.96	5.40	1.60	7.98	5.65	4.71	2.51	8.10	8.13	2.51	4.43	2.17	2.41	3.18	2.65	6.27
BAZ	0.51	0.00	8.62	0.29	4.25	2.99	0.85	5.93	0.06	2.96	6.56	4.13	8.28	6.80	0.74	0.70	3.98	3.25	3.26	3.22	3.90	3.90	8.36	5.81	1.99	8.40	6.08	5.20	2.96	8.52	8.56	2.97	4.93	2.48	2.88	3.67	3.12	6.73
CAV	8.20	8.62	0.00	8.87	4.46	5.63	7.77	2.69	8.56	5.76	2.51	4.67	0.48	2.38	8.72	8.71	4.97	5.55	5.55	5.48	4.88	5.05	0.39	2.81	6.62	0.25	2.55	4.06	5.71	0.31	0.34	5.76	4.45	8.61	5.84	5.46	5.63	2.40
DOS	0.79	0.29	8.87	0.00	4.51	3.24	1.09	6.18	0.35	3.23	6.83	4.40	8.53	7.08	0.63	0.59	4.26	3.52	3.53	3.49	4.17	4.18	8.61	6.06	2.25	8.65	6.33	5.48	3.23	8.78	8.82	3.25	5.21	2.66	3.15	3.96	3.39	7.00
EBE	3.79	4.25	4.46	4.51	0.00	1.32	3.43	1.83	4.18	1.31	2.32	0.33	4.18	2.58	4.48	4.46	0.72	1.09	1.09	1.04	0.46	0.77	4.25	1.74	2.29	4.26	1.91	1.29	1.28	4.33	4.36	1.30	1.29	4.26	1.39	1.21	1.17	2.50
FAL	2.57	2.99	5.63	3.24	1.32	0.00	2.15	2.94	2.93	0.53	3.64	1.31	5.31	3.89	3.17	3.16	1.36	0.75	0.78	0.53	1.10	1.32	5.39	2.83	1.00	5.41	3.09	2.49	0.35	5.54	5.57	0.56	2.36	3.53	0.54	1.43	0.61	3.81
FEN	0.56	0.85	7.77	1.09	3.43	2.15	0.00	5.08	0.79	2.17	5.75	3.33	7.43	6.00	1.12	1.10	3.21	2.47	2.48	2.42	3.10	3.15	7.52	4.97	1.15	7.55	5.24	4.44	2.15	7.68	7.72	2.19	4.20	2.59	2.09	2.97	2.33	5.92
FUR	5.51	5.93	2.69	6.18	1.83	2.94	5.08	0.00	5.87	3.10	1.34	2.09	2.37	1.55	6.04	6.04	2.45	2.92	2.92	2.83	2.28	2.52	2.45	0.14	3.93	2.47	0.28	1.95	3.03	2.61	2.65	3.10	2.29	6.09	3.17	2.96	2.98	1.47
GAV	0.45	0.06	8.56	0.35	4.18	2.93	0.79	5.87	0.00	2.89	6.50	4.07	8.22	6.74	0.77	0.73	3.91	3.18	3.19	3.16	3.83	3.84	8.31	5.75	1.94	8.34	6.02	5.14	2.90	8.46	8.50	2.91	4.87	2.45	2.82	3.61	3.05	6.67
GNI	2.49	2.96	5.76	3.23	1.31	0.53	2.17	3.10	2.89	0.00	3.60	1.17	5.47	3.85	3.26	3.24	1.07	0.33	0.35	0.28	0.94	1.01	5.54	3.00	1.09	5.55	3.21	2.29	0.18	5.64	5.67	0.04	2.08	3.13	0.08	0.98	0.17	3.78
GOS	6.09	6.56	2.51	6.83	2.32	3.64	5.75	1.34	6.50	3.60	0.00	2.43	2.42	0.26	6.80	6.79	2.65	3.33	3.32	3.34	2.66	2.72	2.46	1.45	4.61	2.38	1.07	1.58	3.60	2.29	2.30	3.59	1.96	6.20	3.68	3.08	3.45	0.18
HIR	3.66	4.13	4.67	4.40	0.33	1.31	3.33	2.09	4.07	1.17	2.43	0.00	4.42	2.68	4.41	4.40	0.38	0.90	0.89	0.92	0.23	0.44	4.49	2.02	2.22	4.48	2.15	1.18	1.19	4.53	4.56	1.16	1.10	3.99	1.25	0.89	1.02	2.60
HOD	7.88	8.28	0.48	8.53	4.18	5.31	7.43	2.37	8.22	5.47	2.42	4.42	0.00	2.34	8.35	8.35	4.74	5.27	5.27	5.19	4.62	4.81	0.09	2.48	6.29	0.25	2.27	3.91	5.40	0.68	0.73	5.47	4.30	8.39	5.54	5.24	5.34	2.34
HUS	6.34	6.80	2.38	7.08	2.58	3.89	6.00	1.55	6.74	3.85	0.26	2.68	2.34	0.00	7.05	7.04	2.88	3.57	3.56	3.58	2.91	2.95	2.37	1.67	4.87	2.28	1.28	1.77	3.85	2.14	2.15	3.83	2.15	6.40	3.92	3.30	3.69	0.09
CHAS	1.13	0.74	8.72	0.63	4.48	3.17	1.12	6.04	0.77	3.26	6.80	4.41	8.35	7.05	0.00	0.05	4.32	3.57	3.58	3.50	4.18	4.25	8.44	5.92	2.19	8.49	6.22	5.54	3.22	8.65	8.69	3.28	5.31	3.22	3.18	4.09	3.42	6.98
FED	1.09	0.70	8.71	0.59	4.46	3.16	1.10	6.04	0.73	3.24	6.79	4.40	8.35	7.04	0.05	0.00	4.30	3.55	3.57	3.48	4.17	4.23	8.44	5.91	2.18	8.49	6.21	5.52	3.21	8.64	8.68	3.26	5.29	3.18	3.17	4.06	3.41	6.97
JAK	3.49	3.98	4.97	4.26	0.72	1.36	3.21	2.45	3.91	1.07	2.65	0.38	4.74	2.88	4.32	4.30	0.00	0.75	0.73	0.87	0.36	0.08	4.80	2.38	2.16	4.79	2.47	1.23	1.14	4.81	4.84	1.04	1.02	3.66	1.14	0.52	0.90	2.81
KAM	2.77	3.25	5.55	3.52	1.09	0.75	2.47	2.92	3.18	0.33	3.33	0.90	5.27	3.57	3.57	3.55	0.75	0.00	0.02	0.25	0.67	0.68	5.34	2.83	1.42	5.35	3.00	1.97	0.44	5.41	5.44	0.30	1.75	3.21	0.39	0.68	0.16	3.50
KOL	2.78	3.26	5.55	3.53	1.09	0.78	2.48	2.92	3.19	0.35	3.32	0.89	5.27	3.56	3.58	3.57	0.73	0.02	0.00	0.27	0.67	0.67	5.35	2.83	1.43	5.35	3.00	1.96	0.47	5.41	5.44	0.32	1.74	3.20	0.41	0.66	0.19	3.49
LAG	2.76	3.22	5.48	3.49	1.04	0.53	2.42	2.83	3.16	0.28	3.34	0.92	5.19	3.58	3.50	3.48	0.87	0.25	0.27	0.00	0.68	0.82	5.27	2.73	1.31	5.28	2.93	2.06	0.28	5.36	5.39	0.27	1.88	3.37	0.35	0.91	0.18	3.51
LOI	3.43	3.90	4.88	4.17	0.46	1.10	3.10	2.28	3.83	0.94	2.66	0.23	4.62	2.91	4.18	4.17	0.36	0.67	0.67	0.68	0.00	0.38	4.69	2.20	1.99	4.69	2.35	1.40	0.96	4.75	4.78	0.92	1.27	3.81	1.02	0.78	0.78	2.84
LOR	3.42	3.90	5.05	4.18	0.77	1.32	3.15	2.52	3.84	1.01	2.72	0.44	4.81	2.95	4.25	4.23	0.08	0.68	0.67	0.82	0.38	0.00	4.88	2.45	2.10	4.86	2.55	1.29	1.09	4.89	4.91	0.98	1.07	3.58	1.08	0.45	0.84	2.89
LYS	7.96	8.36	0.39	8.61	4.25	5.39	7.52	2.45	8.31	5.54	2.46	4.49	0.09	2.37	8.44	8.44	4.80	5.34	5.35	5.27	4.69	4.88	0.00	2.56	6.37	0.19	2.34	3.96	5.48	0.61	0.66	5.54	4.35	8.46	5.62	5.30	5.42	2.37
MAR	5.40	5.81	2.81	6.06	1.74	2.83	4.97	0.14	5.75	3.00	1.45	2.02	2.48	1.67	5.92	5.91	2.38	2.83	2.83	2.73	2.20	2.45	2.56	0.00	3.82	2.59	0.40	1.96	2.93	2.73	2.77	3.00	2.28	6.01	3.07	2.90	2.89	1.59
MIS	1.60	1.99	6.62	2.25	2.29	1.00	1.15	3.93	1.94	1.09	4.61	2.22	6.29	4.87	2.19	2.18	2.16	1.42	1.43	1.31	1.99	2.10	6.37	3.82	0.00	6.40	4.09	3.37	1.03	6.53	6.57	1.12	3.17	2.95	1.02	2.02	1.26	4.79
OMS	7.98	8.40	0.25	8.65	4.26	5.41	7.55	2.47	8.34	5.55	2.38	4.48	0.25	2.28	8.49	8.49	4.79	5.35	5.35	5.28	4.69	4.86	0.19	2.59	6.40	0.00	2.35	3.91	5.50	0.43	0.47	5.55	4.30	8.44	5.63	5.28	5.43	2.28
PIT	5.65	6.08	2.55	6.33	1.91	3.09	5.24	0.28	6.02	3.21	1.07	2.15	2.27	1.28	6.22	6.21	2.47	3.00	3.00	2.93	2.35	2.55	2.34	0.40	4.09	2.35	0.00	1.84	3.15	2.45	2.48	3.20	2.21	6.13	3.28	2.98	3.08	1.19
POD	4.71	5.20	4.06	5.48	1.29	2.49	4.44	1.95	5.14	2.29	1.58	1.18	3.91	1.77	5.54	5.52	1.23	1.97	1.96	2.06	1.40	1.29	3.96	1.96	3.37	3.91	1.84	0.00	2.34	3.85	3.87	2.26	0.39	4.63	2.36	1.57	2.12	1.72
PON	2.51	2.96	5.71	3.23	1.28	0.35	2.15	3.03	2.90	0.18	3.60	1.19	5.40	3.85	3.22	3.21	1.14	0.44	0.47	0.28	0.96	1.09	5.48	2.93	1.03	5.50	3.15	2.34	0.00	5.59	5.63	0.21	2.16	3.26	0.20	1.12	0.28	3.77
RAD	8.10	8.52	0.31	8.78	4.33	5.54	7.68	2.61	8.46	5.64	2.29	4.53	0.68	2.14	8.65	8.64	4.81	5.41	5.41	5.36	4.75	4.89	0.61	2.73	6.53	0.43	2.45	3.85	5.59	0.00	0.05	5.63	4.24	8.44	5.71	5.29	5.50	2.17
STR	8.13	8.56	0.34	8.82	4.36	5.57	7.72	2.65	8.50	5.67	2.30	4.56	0.73	2.15	8.69	8.68	4.84	5.44	5.44	5.39	4.78	4.91	0.66	2.77	6.57	0.47	2.48	3.87	5.63	0.05	0.00	5.66	4.26	8.46	5.75	5.32	5.53	2.18
TAN	2.51	2.97	5.76	3.25	1.30	0.56	2.19	3.10	2.91	0.04	3.59	1.16	5.47	3.83	3.28	3.26	1.04	0.30	0.32	0.27	0.92	0.98	5.54	3.00	1.12	5.55	3.20	2.26	0.21	5.63	5.66	0.00	2.05	3.11	0.10	0.95	0.14	3.76
TRD	4.43	4.93	4.45	5.21	1.29	2.36	4.20	2.29	4.87	2.08	1.96	1.10	4.30	2.15	5.31	5.29	1.02	1.75	1.74	1.88	1.27	1.07	4.35	2.28	3.17	4.30	2.21	0.39	2.16	4.24	4.26	2.05	0.00	4.25	2.15	1.26	1.91	2.10
VAL	2.17	2.48	8.61	2.66	4.26	3.53	2.59	6.09	2.45	3.13	6.20	3.99	8.39	6.40	3.22	3.18	3.66	3.21	3.20	3.37	3.81	3.58	8.46	6.01	2.95	8.44	6.13	4.63	3.26	8.44	8.46	3.11	4.25	0.00	3.07	3.16	3.19	6.35
VOD	2.41	2.88	5.84	3.15	1.39	0.54	2.09	3.17	2.82	0.08	3.68	1.25	5.54	3.92	3.18	3.17	1.14	0.39	0.41	0.35	1.02	1.08	5.62	3.07	1.02	5.63	3.28	2.36	0.20	5.71	5.75	0.10	2.15	3.07	0.00	1.03	0.24	3.85
VRE	3.18	3.67	5.46	3.96	1.21	1.43	2.97	2.96	3.61	0.98	3.08	0.89	5.24	3.30	4.09	4.06	0.52	0.68	0.66	0.91	0.78	0.45	5.30	2.90	2.02	5.28	2.98	1.57	1.12	5.29	5.32	0.95	1.26	3.16	1.03	0.00	0.84	3.25
ZAG	2.65	3.12	5.63	3.39	1.17	0.61	2.33	2.98	3.05	0.17	3.45	1.02	5.34	3.69	3.42	3.41	0.90	0.16	0.19	0.18	0.78	0.84	5.42	2.89	1.26	5.43	3.08	2.12	0.28	5.50	5.53	0.14	1.91	3.19	0.24	0.84	0.00	3.62
ZAL	6.27	6.73	2.40	7.00	2.50	3.81	5.92	1.47	6.67	3.78	0.18	2.60	2.34	0.09	6.98	6.97	2.81	3.50	3.49	3.51	2.84	2.89	2.37	1.59	4.79	2.28	1.19	1.72	3.77	2.17	2.18	3.76	2.10	6.35	3.85	3.25	3.62	0.00

b) Taxonomic affiliation

Locality	BAL	BAZ	CAV	DOS	EBE	FAL	FED	FEN	FUR	GAV	GNI	GOS	HIR	HOD	HUS	CHAS	JAK	KAM	KOL	LAG	LOI	LOR	LYS	MAR	MIS	OMS	PIT	POD	PON	RAD	STR	TAN	TRD	VAL	VOD	VRE	ZAG	ZAL
BAL	**0**	1	0	1	0	0	1	1	0	1	0	0	0	0	0	1	0	0	0	0	0	0	0	0	1	0	0	0	0	0	0	0	0	0	0	0	0	0
BAZ	1	**0**	0	1	0	0	1	1	0	1	0	0	0	0	0	1	0	0	0	0	0	0	0	0	1	0	0	0	0	0	0	0	0	0	0	0	0	0
CAV	0	0	**0**	0	0	0	0	0	0	0	0	0	0	1	0	0	0	0	0	0	0	0	1	0	0	1	0	0	0	1	1	0	0	0	0	0	0	0
DOS	1	1	**0**	**0**	0	0	1	1	0	1	0	0	0	0	0	1	0	0	0	0	0	0	0	0	1	0	0	0	0	0	0	0	0	0	0	0	0	0
EBE	0	0	0	0	**0**	1	0	**0**	1	0	0	0	1	0	0	0	1	0	0	0	1	1	0	1	0	0	1	1	0	0	0	0	1	0	0	1	0	0
FAL	0	0	0	0	1	**0**	0	0	1	0	0	0	1	0	0	0	1	0	0	0	1	1	0	1	0	0	1	1	0	0	0	0	1	0	0	1	0	0
FED	1	1	0	1	0	0	**0**	1	0	1	0	0	0	0	0	1	0	0	0	0	0	0	0	0	1	0	0	0	0	0	0	0	0	0	0	0	0	0
FEN	1	1	0	1	0	0	1	**0**	0	1	0	0	0	0	0	1	0	0	0	0	0	0	0	0	1	0	0	0	0	0	0	0	0	0	0	0	0	0
FUR	0	0	0	0	1	1	0	0	**0**	0	0	0	1	0	0	0	1	0	0	0	1	1	0	1	0	0	1	1	0	0	0	0	1	0	0	1	0	0
GAV	1	1	0	1	0	0	1	1	0	**0**	0	0	0	0	0	1	0	0	0	0	0	0	0	0	1	0	0	0	0	0	0	0	0	0	0	0	0	0
GNI	0	0	0	0	0	0	0	0	0	0	**0**	0	0	0	0	0	0	1	1	1	0	0	0	0	0	0	0	0	1	0	0	1	0	0	1	0	1	0
GOS	0	0	0	0	0	0	0	0	0	0	0	**0**	0	0	1	0	0	0	0	0	0	0	0	0	0	0	0	0	0	0	0	0	0	0	0	0	0	1
HIR	0	0	0	0	1	1	0	0	1	0	0	0	**0**	0	0	0	1	0	0	0	1	1	0	1	0	0	1	1	0	0	0	0	1	0	0	1	0	0
HOD	0	0	1	0	0	0	0	0	0	0	0	0	0	**0**	0	0	0	0	0	0	0	0	1	0	0	1	0	0	0	1	1	0	0	0	0	0	0	0
HUS	0	0	0	0	0	0	0	0	0	0	0	1	0	0	**0**	0	0	0	0	0	0	0	0	0	0	0	0	0	0	0	0	0	0	0	0	0	0	1
CHAS	1	1	0	1	0	0	1	1	0	1	0	0	0	0	0	**0**	0	0	0	0	0	0	0	0	1	0	0	0	0	0	0	0	0	0	0	0	0	0
JAK	0	0	0	0	1	1	0	0	1	0	0	0	1	0	0	0	**0**	0	0	0	1	1	0	1	0	0	1	1	0	0	0	0	1	0	0	1	0	0
KAM	0	0	0	0	0	0	0	0	0	0	1	0	0	0	0	0	0	**0**	1	1	0	0	0	0	0	0	0	0	1	0	0	1	0	0	1	0	1	0
KOL	0	0	0	0	0	0	0	0	0	0	1	0	0	0	0	0	0	1	**0**	1	0	0	0	0	0	0	0	0	1	0	0	1	0	0	1	0	1	0
LAG	0	0	0	0	0	0	0	0	0	0	1	0	0	0	0	0	0	1	1	**0**	0	0	0	0	0	0	0	0	1	0	0	1	0	0	1	0	1	0
LOI	0	0	0	0	1	1	0	0	1	0	0	0	1	0	0	0	1	0	0	0	**0**	1	0	1	0	0	1	1	0	0	0	0	1	0	0	1	0	0
LOR	0	0	0	0	1	1	0	0	1	0	0	0	1	0	0	0	1	0	0	0	1	**0**	0	1	0	0	1	1	0	0	0	0	1	0	0	1	0	0
LYS	0	0	1	0	0	0	0	0	0	0	0	0	0	1	0	0	0	0	0	0	0	0	**0**	0	0	1	0	0	0	1	1	0	0	0	0	0	0	0
MAR	0	0	0	0	1	1	0	0	1	0	0	0	1	0	0	0	1	0	0	0	1	1	0	**0**	0	0	1	1	0	0	0	0	1	0	0	1	0	0
MIS	1	1	0	1	0	0	1	1	0	1	0	0	0	0	0	1	0	0	0	0	0	0	0	0	**0**	0	0	0	0	0	0	0	0	0	0	0	0	0
OMS	0	0	1	0	0	0	0	0	0	0	0	0	0	1	0	0	0	0	0	0	0	0	1	0	0	**0**	0	0	0	1	1	0	0	0	0	0	0	0
PIT	0	0	0	0	1	1	0	0	1	0	0	0	1	0	0	0	1	0	0	0	1	1	0	1	0	0	**0**	1	0	0	0	0	1	0	0	1	0	0
POD	0	0	0	0	1	1	0	0	1	0	0	0	1	0	0	0	1	0	0	0	1	1	0	1	0	0	1	**0**	0	0	0	0	1	0	0	1	0	0
PON	0	0	0	0	0	0	0	0	0	0	1	0	0	0	0	0	0	1	1	1	0	0	0	0	0	0	0	0	**0**	0	0	1	0	0	1	0	1	0
RAD	0	0	1	0	0	0	0	0	0	0	0	0	0	1	0	0	0	0	0	0	0	0	1	0	0	1	0	0	0	**0**	1	0	0	0	0	0	0	0
STR	0	0	1	0	0	0	0	0	0	0	0	0	0	1	0	0	0	0	0	0	0	0	1	0	0	1	0	0	0	1	**0**	0	0	0	0	0	0	0
TAN	0	0	0	0	0	0	0	0	0	0	1	0	0	0	0	0	0	1	1	1	0	0	0	0	0	0	0	0	1	0	0	**0**	0	0	1	0	1	0
TRD	0	0	0	0	1	1	0	0	1	0	0	0	1	0	0	0	1	0	0	0	1	1	0	1	0	0	1	1	0	0	0	0	**0**	0	0	1	0	0
VAL	0	0	0	0	0	0	0	0	0	0	0	0	0	0	0	0	0	0	0	0	0	0	0	0	0	0	0	0	0	0	0	0	0	**0**	0	0	0	0
VOD	0	0	0	0	0	0	0	0	0	0	1	0	0	0	0	0	0	1	1	1	0	0	0	0	0	0	0	0	1	0	0	1	0	0	**0**	0	1	0
VRE	0	0	0	0	1	1	0	0	1	0	0	0	1	0	0	0	1	0	0	0	1	1	0	1	0	0	1	1	0	0	0	0	1	0	0	**0**	0	0
ZAG	0	0	0	0	0	0	0	0	0	0	1	0	0	0	0	0	0	1	1	1	0	0	0	0	0	0	0	0	1	0	0	1	0	0	1	0	**0**	0
ZAL	0	0	0	0	0	0	0	0	0	0	0	1	0	0	1	0	0	0	0	0	0	0	0	0	0	0	0	0	0	0	0	0	0	0	0	0	0	**0**

c) Morphometrics

Locality	BAL	BAZ	CAV	EBE	FAL	GAV	GNI	GOS	HIR	HOD	HUS	CHAS	JAK	KAM	KOL	LAG	LOI	LOR	LYS	MAR	MIS	OMS	PIT	POD	PON	RAD	STR	TAN	TRD	VAL	VRE	ZAG	ZAL
BAL	0	0.29	0.84	1.17	1.24	0.25	1.83	1.22	0.59	0.6	1.26	1.67	0.62	1.9	1.23	1.69	0.95	0.61	0.89	0.8	0.23	0.96	0.92	1.05	1.88	1.18	1.3	1.73	1.38	2.97	0.56	1.14	0.55
BAZ	0.29	0	0.94	1.1	1.24	0.05	1.87	1.51	0.45	0.68	1.53	1.72	0.54	1.87	1.19	1.63	0.98	0.35	1.15	0.52	0.21	1.18	0.63	0.9	1.9	1.32	1.5	1.79	1.17	3.01	0.27	1.17	0.84
CAV	0.84	0.94	0	0.66	0.52	0.9	1.01	1.28	0.69	0.26	1.03	2.47	0.56	1.17	0.61	1.06	0.26	1.27	0.67	1.19	0.74	0.53	1.37	0.86	1.09	0.39	0.7	0.9	1.28	2.14	1.13	0.4	0.96
EBE	1.17	1.1	0.66	0	0.31	1.09	0.9	1.94	0.66	0.68	1.69	2.82	0.57	0.77	0.15	0.52	0.4	1.3	1.33	1.03	0.97	1.19	1.21	0.39	0.88	0.88	1.3	0.89	0.72	1.98	1.15	0.38	1.52
FAL	1.24	1.24	0.52	0.31	0	1.21	0.65	1.79	0.83	0.66	1.49	2.9	0.71	0.67	0.17	0.54	0.3	1.49	1.17	1.27	1.07	1	1.46	0.69	0.66	0.61	1.04	0.6	1.03	1.78	1.34	0.13	1.47
GAV	0.25	0.05	0.9	1.09	1.21	0	1.84	1.47	0.44	0.64	1.48	1.73	0.52	1.85	1.17	1.61	0.95	0.4	1.1	0.56	0.16	1.13	0.67	0.9	1.87	1.28	1.46	1.75	1.18	2.98	0.32	1.14	0.8
GNI	1.83	1.87	1.01	0.9	0.65	1.84	0	2.08	1.48	1.23	1.7	3.49	1.35	0.46	0.76	0.67	0.9	2.14	1.51	1.92	1.69	1.3	2.1	1.29	0.15	0.82	1.15	0.16	1.55	1.14	1.99	0.7	1.95
GOS	1.22	1.51	1.28	1.94	1.79	1.47	2.08	0	1.66	1.3	0.43	2.02	1.6	2.38	1.89	2.33	1.54	1.82	0.61	2.01	1.39	0.8	2.14	2.04	2.2	1.27	0.96	1.92	2.44	3.05	1.78	1.66	0.69
HIR	0.59	0.45	0.69	0.66	0.83	0.44	1.48	1.66	0	0.47	1.56	2.17	0.14	1.43	0.76	1.18	0.62	0.66	1.16	0.49	0.36	1.11	0.68	0.46	1.49	1.08	1.37	1.42	0.81	2.61	0.5	0.79	1.07
HOD	0.6	0.68	0.26	0.68	0.66	0.64	1.23	1.3	0.47	0	1.12	2.26	0.35	1.32	0.69	1.16	0.36	1.01	0.73	0.95	0.48	0.65	1.13	0.75	1.29	0.65	0.91	1.13	1.16	2.37	0.87	0.55	0.84
HUS	1.26	1.53	1.03	1.69	1.49	1.48	1.7	0.43	1.56	1.12	0	2.36	1.46	2.04	1.62	2.02	1.29	1.87	0.4	1.98	1.36	0.5	2.13	1.87	1.83	0.92	0.55	1.54	2.28	2.63	1.8	1.37	0.83
CHAS	1.67	1.72	2.47	2.82	2.9	1.73	3.49	2.02	2.17	2.26	2.36	0	2.25	3.56	2.89	3.34	2.61	1.65	2.2	2.03	1.85	2.38	1.98	2.61	3.54	2.76	2.71	3.37	2.81	4.61	1.77	2.81	1.63
JAK	0.62	0.54	0.56	0.57	0.71	0.52	1.35	1.6	0.14	0.35	1.46	2.25	0	1.32	0.65	1.09	0.48	0.79	1.07	0.63	0.41	1	0.82	0.46	1.37	0.94	1.25	1.28	0.85	2.48	0.64	0.66	1.05
KAM	1.9	1.87	1.17	0.77	0.67	1.85	0.46	2.38	1.43	1.32	2.04	3.56	1.32	0	0.67	0.31	0.97	2.07	1.78	1.79	1.71	1.58	1.95	1.11	0.32	1.12	1.51	0.59	1.26	1.25	1.92	0.78	2.13
KOL	1.23	1.19	0.61	0.15	0.17	1.17	0.76	1.89	0.76	0.69	1.62	2.89	0.65	0.67	0	0.47	0.35	1.41	1.28	1.16	1.04	1.12	1.34	0.54	0.74	0.77	1.19	0.74	0.86	1.85	1.26	0.26	1.52
LAG	1.69	1.63	1.06	0.52	0.54	1.61	0.67	2.33	1.18	1.16	2.02	3.34	1.09	0.31	0.47	0	0.82	1.8	1.72	1.5	1.49	1.54	1.66	0.82	0.57	1.11	1.54	0.76	0.95	1.56	1.65	0.67	1.99
LOI	0.95	0.98	0.26	0.4	0.3	0.95	0.9	1.54	0.62	0.36	1.29	2.61	0.48	0.97	0.35	0.82	0	1.26	0.93	1.1	0.79	0.78	1.29	0.65	0.94	0.53	0.91	0.81	1.05	2.04	1.11	0.2	1.18
LOR	0.61	0.35	1.27	1.3	1.49	0.4	2.14	1.82	0.66	1.01	1.87	1.65	0.79	2.07	1.41	1.8	1.26	0	1.5	0.39	0.55	1.53	0.38	1	2.15	1.65	1.85	2.07	1.16	3.26	0.15	1.44	1.14
LYS	0.89	1.15	0.67	1.33	1.17	1.1	1.51	0.61	1.16	0.73	0.4	2.2	1.07	1.78	1.28	1.72	0.93	1.5	0	1.58	0.97	0.21	1.74	1.48	1.62	0.69	0.5	1.35	1.89	2.54	1.41	1.05	0.58
MAR	0.8	0.52	1.19	1.03	1.27	0.56	1.92	2.01	0.49	0.95	1.98	2.03	0.63	1.79	1.16	1.5	1.1	0.39	1.58	0	0.63	1.57	0.19	0.68	1.91	1.57	1.85	1.87	0.77	3.01	0.29	1.26	1.35
MIS	0.23	0.21	0.74	0.97	1.07	0.16	1.69	1.39	0.36	0.48	1.36	1.85	0.41	1.71	1.04	1.49	0.79	0.55	0.97	0.63	0	0.99	0.77	0.83	1.73	1.12	1.3	1.6	1.16	2.83	0.45	0.99	0.75
OMS	0.96	1.18	0.53	1.19	1	1.13	1.3	0.8	1.11	0.65	0.5	2.38	1	1.58	1.12	1.54	0.78	1.53	0.21	1.57	0.99	0	1.74	1.38	1.41	0.48	0.34	1.14	1.79	2.33	1.43	0.87	0.75
PIT	0.92	0.63	1.37	1.21	1.46	0.67	2.1	2.14	0.68	1.13	2.13	1.98	0.82	1.95	1.34	1.66	1.29	0.38	1.74	0.19	0.77	1.74	0	0.84	2.08	1.76	2.03	2.06	0.87	3.19	0.36	1.44	1.47
POD	1.05	0.9	0.86	0.39	0.69	0.9	1.29	2.04	0.46	0.75	1.87	2.61	0.46	1.11	0.54	0.82	0.65	1	1.48	0.68	0.83	1.38	0.84	0	1.26	1.18	1.56	1.28	0.42	2.35	0.86	0.72	1.51
PON	1.88	1.9	1.09	0.88	0.66	1.87	0.15	2.2	1.49	1.29	1.83	3.54	1.37	0.32	0.74	0.57	0.94	2.15	1.62	1.91	1.73	1.41	2.08	1.26	0	0.93	1.29	0.3	1.49	1.12	1.99	0.74	2.04
RAD	1.18	1.32	0.39	0.88	0.61	1.28	0.82	1.27	1.08	0.65	0.92	2.76	0.94	1.12	0.77	1.11	0.53	1.65	0.69	1.57	1.12	0.48	1.76	1.18	0.93	0	0.43	0.67	1.57	1.88	1.52	0.51	1.16
STR	1.3	1.5	0.7	1.3	1.04	1.46	1.15	0.96	1.37	0.91	0.55	2.71	1.25	1.51	1.19	1.54	0.91	1.85	0.5	1.85	1.3	0.34	2.03	1.56	1.29	0.43	0	0.99	1.97	2.09	1.74	0.93	1.08
TAN	1.73	1.79	0.9	0.89	0.6	1.75	0.16	1.92	1.42	1.13	1.54	3.37	1.28	0.59	0.74	0.76	0.81	2.07	1.35	1.87	1.6	1.14	2.06	1.28	0.3	0.67	0.99	0	1.58	1.25	1.92	0.63	1.81
TRD	1.38	1.17	1.28	0.72	1.03	1.18	1.55	2.44	0.81	1.16	2.28	2.81	0.85	1.26	0.86	0.95	1.05	1.16	1.89	0.77	1.16	1.79	0.87	0.42	1.49	1.57	1.97	1.58	0	2.51	1.04	1.09	1.88
VAL	2.97	3.01	2.14	1.98	1.78	2.98	1.14	3.05	2.61	2.37	2.63	4.61	2.48	1.25	1.85	1.56	2.04	3.26	2.54	3.01	2.83	2.33	3.19	2.35	1.12	1.88	2.09	1.25	2.51	0	3.11	1.84	3.04
VRE	0.56	0.27	1.13	1.15	1.34	0.32	1.99	1.78	0.5	0.87	1.8	1.77	0.64	1.92	1.26	1.65	1.11	0.15	1.41	0.29	0.45	1.43	0.36	0.86	1.99	1.52	1.74	1.92	1.04	3.11	0	1.29	1.11
ZAG	1.14	1.17	0.4	0.38	0.13	1.14	0.7	1.66	0.79	0.55	1.37	2.81	0.66	0.78	0.26	0.67	0.2	1.44	1.05	1.26	0.99	0.87	1.44	0.72	0.74	0.51	0.93	0.63	1.09	1.84	1.29	0	1.35
ZAL	0.55	0.84	0.96	1.52	1.47	0.8	1.95	0.69	1.07	0.84	0.83	1.63	1.05	2.13	1.52	1.99	1.18	1.14	0.58	1.35	0.75	0.75	1.47	1.51	2.04	1.16	1.08	1.81	1.88	3.04	1.11	1.35	0

d) Genome size

Locality	BAL	BAZ	CAV	DOS	EBE	FAL	FED	FEN	FUR	GAV	GNI	GOS	HIR	JAK	KAM	KOL	LAG	LOI	LOR	LYS	MAR	MIS	OMS	PIT	POD	PON	RAD	STR	TAN	VAL	VRE	ZAG	ZAL
BAL	0	0.1650173	0.93516162	0.1614458	0.94248886	0.81375291	0.15754285	0.01881059	1.20836256	0.03142695	0.59794613	1.20074958	1.00225009	1.10584765	0.59351071	0.57999268	0.62505363	0.98698499	1.04363347	0.79407319	1.13767141	0.20558599	1.07944116	1.09370283	1.03334623	0.65025395	1.0114492	1.25245201	0.65345348	0.89909468	1.08419375	0.57400228	1.20698083
BAZ	0.1650173	0	0.77014432	0.00357151	0.77747156	0.64873561	0.00747445	0.18382789	1.04334525	0.13359035	0.43292882	1.03573227	0.83723279	0.94083035	0.42849341	0.41497538	0.46003633	0.82196768	0.87861616	0.62905588	0.97265411	0.04056868	0.91442386	0.92868552	0.86832893	0.48523664	0.8464319	1.08743471	0.48843618	1.06411198	0.91917644	0.40898497	1.04196353
CAV	0.93516162	0.77014432	0	0.77371582	0.00732724	0.12140871	0.77761877	0.95397221	0.27320093	0.90373467	0.3372155	0.26558796	0.06708847	0.17068603	0.34165091	0.35516894	0.31010799	0.05182337	0.10847185	0.14108844	0.20250979	0.72957563	0.14427954	0.1585412	0.09818461	0.28490767	0.07628758	0.31729039	0.28170814	1.8342563	0.14903213	0.36115934	0.27181921
DOS	0.1614458	0.00357151	0.77371582	0	0.78104306	0.65230711	0.00390295	0.18025638	1.04691676	0.13001885	0.43650033	1.03930378	0.84080429	0.94440185	0.43206492	0.41854688	0.46360783	0.82553919	0.88218767	0.63262739	0.97622561	0.04414019	0.91799536	0.93225703	0.87190043	0.48880815	0.8500034	1.09100621	0.49200769	1.06054048	0.92274795	0.41255648	1.04553503
EBE	0.94248886	0.77747156	0.00732724	0.78104306	0	0.12873595	0.78494601	0.96129945	0.26587369	0.91106191	0.34454274	0.25826072	0.05976123	0.16335879	0.34897815	0.36249618	0.31743523	0.04449613	0.10114461	0.14841568	0.19518255	0.73690287	0.1369523	0.15121397	0.09085737	0.29223491	0.06896034	0.30996315	0.28903538	1.84158354	0.14170489	0.36848658	0.26449197
FAL	0.81375291	0.64873561	0.12140871	0.65230711	0.12873595	0	0.65621006	0.8325635	0.39460964	0.78232596	0.21580679	0.38699667	0.18849718	0.29209474	0.2202422	0.23376023	0.18869928	0.17323207	0.22988055	0.01967973	0.3239185	0.60816692	0.26568825	0.27994991	0.21959332	0.16349897	0.19769629	0.4386991	0.16029943	1.71284759	0.27044084	0.23975063	0.39322792
FED	0.15754285	0.00747445	0.77761877	0.00390295	0.78494601	0.65621006	0	0.17635344	1.0508197	0.1261159	0.44040327	1.04320673	0.84470724	0.9483048	0.43596786	0.42244983	0.46751078	0.82944214	0.88609062	0.63653033	0.98012856	0.04804314	0.92189831	0.93615997	0.87580338	0.4927111	0.85390635	1.09490916	0.49591063	1.05663753	0.9266509	0.41645943	1.04943798
FEN	0.01881059	0.18382789	0.95397221	0.18025638	0.96129945	0.8325635	0.17635344	0	1.22717314	0.05023754	0.61675671	1.21956016	1.02106068	1.12465824	0.6123213	0.59880327	0.64386422	1.00579557	1.06244405	0.81288377	1.156482	0.22439657	1.09825175	1.11251341	1.05215682	0.66906453	1.03025979	1.2712626	0.67226407	0.88028409	1.10300433	0.59281286	1.22579142
FUR	1.20836256	1.04334525	0.27320093	1.04691676	0.26587369	0.39460964	1.0508197	1.22717314	0	1.1769356	0.61041643	0.00761298	0.20611246	0.1025149	0.61485184	0.62836987	0.58330892	0.22137757	0.16472909	0.41428937	0.07069114	1.00277657	0.12892139	0.11465973	0.17501632	0.55810861	0.19691335	0.04408946	0.55490907	2.10745723	0.12416881	0.63436028	0.00138172
GAV	0.03142695	0.13359035	0.90373467	0.13001885	0.91106191	0.78232596	0.1261159	0.05023754	1.1769356	0	0.56651917	1.16932263	0.97082314	1.0744207	0.56208376	0.54856573	0.59362668	0.95555804	1.01220652	0.76264623	1.10624446	0.17415904	1.04801421	1.06227587	1.00191928	0.618827	0.98002225	1.22102506	0.62202653	0.93052163	1.0527668	0.54257533	1.17555388
GNI	0.59794613	0.43292882	0.3372155	0.43650033	0.34454274	0.21580679	0.44040327	0.61675671	0.61041643	0.56651917	0	0.60280345	0.40430397	0.50790153	0.00443541	0.01795344	0.02710751	0.38903886	0.44568734	0.19612706	0.53972529	0.39236014	0.48149504	0.4957567	0.43540011	0.05230782	0.41350308	0.65450589	0.05550736	1.4970408	0.48624762	0.02394385	0.60903471
GOS	1.20074958	1.03573227	0.26558796	1.03930378	0.25826072	0.38699667	1.04320673	1.21956016	0.00761298	1.16932263	0.60280345	0	0.19849948	0.09490193	0.60723886	0.6207569	0.57569595	0.21376459	0.15711611	0.40667639	0.06307817	0.99516359	0.12130842	0.10704675	0.16740335	0.55049563	0.18930038	0.05170243	0.54729609	2.09984426	0.11655583	0.6267473	0.00623125
HIR	1.00225009	0.83723279	0.06708847	0.84080429	0.05976123	0.18849718	0.84470724	1.02106068	0.20611246	0.97082314	0.40430397	0.19849948	0	0.10359756	0.40873938	0.42225741	0.37719646	0.01526511	0.04138337	0.20817691	0.13542132	0.7966641	0.07719107	0.09145273	0.03109614	0.35199615	0.00919911	0.25020192	0.34879661	1.90134477	0.08194365	0.42824782	0.20473074
JAK	1.10584765	0.94083035	0.17068603	0.94440185	0.16335879	0.29209474	0.9483048	1.12465824	0.1025149	1.0744207	0.50790153	0.09490193	0.10359756	0	0.51233694	0.52585497	0.48079402	0.11886266	0.06221418	0.31177447	0.03182376	0.90026166	0.02640649	0.01214483	0.07250142	0.4555937	0.09439845	0.14660436	0.45239417	2.00494233	0.0216539	0.53184537	0.10113318
KAM	0.59351071	0.42849341	0.34165091	0.43206492	0.34897815	0.2202422	0.43596786	0.6123213	0.61485184	0.56208376	0.00443541	0.60723886	0.40873938	0.51233694	0	0.01351803	0.03154292	0.39347427	0.45012275	0.20056247	0.5441607	0.38792473	0.48593045	0.50019211	0.43983552	0.05674323	0.41793849	0.6589413	0.05994277	1.49260539	0.49068303	0.01950844	0.61347012
KOL	0.57999268	0.41497538	0.35516894	0.41854688	0.36249618	0.23376023	0.42244983	0.59880327	0.62836987	0.54856573	0.01795344	0.6207569	0.42225741	0.52585497	0.01351803	0	0.04506095	0.40699231	0.46364079	0.2140805	0.55767873	0.37440669	0.49944848	0.51371014	0.45335355	0.07026127	0.43145652	0.67245933	0.0734608	1.47908736	0.50420107	0.0059904	0.62698815
LAG	0.62505363	0.46003633	0.31010799	0.46360783	0.31743523	0.18869928	0.46751078	0.64386422	0.58330892	0.59362668	0.02710751	0.57569595	0.37719646	0.48079402	0.03154292	0.04506095	0	0.36193135	0.41857983	0.16901955	0.51261778	0.41946764	0.45438753	0.46864919	0.4082926	0.02520031	0.38639557	0.62739838	0.02839985	1.52414831	0.45914012	0.05105135	0.5819272
LOI	0.98698499	0.82196768	0.05182337	0.82553919	0.04449613	0.17323207	0.82944214	1.00579557	0.22137757	0.95555804	0.38903886	0.21376459	0.01526511	0.11886266	0.39347427	0.40699231	0.36193135	0	0.05664848	0.1929118	0.15068643	0.781399	0.09245618	0.10671784	0.04636125	0.33673104	0.02446422	0.26546703	0.3335315	1.88607966	0.09720876	0.41298271	0.21999584
LOR	1.04363347	0.87861616	0.10847185	0.88218767	0.10114461	0.22988055	0.88609062	1.06244405	0.16472909	1.01220652	0.44568734	0.15711611	0.04138337	0.06221418	0.45012275	0.46364079	0.41857983	0.05664848	0	0.24956028	0.09403795	0.83804748	0.0358077	0.05006936	0.01028723	0.39337952	0.03218426	0.20881855	0.39017998	1.94272815	0.04056028	0.46963119	0.16334736
LYS	0.79407319	0.62905588	0.14108844	0.63262739	0.14841568	0.01967973	0.63653033	0.81288377	0.41428937	0.76264623	0.19612706	0.40667639	0.20817691	0.31177447	0.20056247	0.2140805	0.16901955	0.1929118	0.24956028	0	0.34359823	0.5884872	0.28536798	0.29962964	0.23927305	0.14381924	0.21737602	0.45837883	0.1406197	1.69316786	0.29012056	0.22007091	0.41290765
MAR	1.13767141	0.97265411	0.20250979	0.97622561	0.19518255	0.3239185	0.98012856	1.156482	0.07069114	1.10624446	0.53972529	0.06307817	0.13542132	0.03182376	0.5441607	0.55767873	0.51261778	0.15068643	0.09403795	0.34359823	0	0.93208542	0.05823025	0.04396859	0.10432518	0.48741747	0.12622221	0.1147806	0.48421793	2.03676609	0.05347766	0.56366913	0.06930942
MIS	0.20558599	0.04056868	0.72957563	0.04414019	0.73690287	0.60816692	0.04804314	0.22439657	1.00277657	0.17415904	0.39236014	0.99516359	0.7966641	0.90026166	0.38792473	0.37440669	0.41946764	0.781399	0.83804748	0.5884872	0.93208542	0	0.87385517	0.88811684	0.82776024	0.44466796	0.80586321	1.04686602	0.4478675	1.10468067	0.87860776	0.36841629	1.00139484
OMS	1.07944116	0.91442386	0.14427954	0.91799536	0.1369523	0.26568825	0.92189831	1.09825175	0.12892139	1.04801421	0.48149504	0.12130842	0.07719107	0.02640649	0.48593045	0.49944848	0.45438753	0.09245618	0.0358077	0.28536798	0.05823025	0.87385517	0	0.01426166	0.04609493	0.42918722	0.06799196	0.17301085	0.42598768	1.97853584	0.00475258	0.50543889	0.12753967
PIT	1.09370283	0.92868552	0.1585412	0.93225703	0.15121397	0.27994991	0.93615997	1.11251341	0.11465973	1.06227587	0.4957567	0.10704675	0.09145273	0.01214483	0.50019211	0.51371014	0.46864919	0.10671784	0.05006936	0.29962964	0.04396859	0.88811684	0.01426166	0	0.06035659	0.44344888	0.08225362	0.15874919	0.44024934	1.9927975	0.00950908	0.51970055	0.113278
POD	1.03334623	0.86832893	0.09818461	0.87190043	0.09085737	0.21959332	0.87580338	1.05215682	0.17501632	1.00191928	0.43540011	0.16740335	0.03109614	0.07250142	0.43983552	0.45335355	0.4082926	0.04636125	0.01028723	0.23927305	0.10432518	0.82776024	0.04609493	0.06035659	0	0.38309229	0.02189703	0.21910578	0.37989275	1.93244091	0.05084752	0.45934395	0.1736346
PON	0.65025395	0.48523664	0.28490767	0.48880815	0.29223491	0.16349897	0.4927111	0.66906453	0.55810861	0.618827	0.05230782	0.55049563	0.35199615	0.4555937	0.05674323	0.07026127	0.02520031	0.33673104	0.39337952	0.14381924	0.48741747	0.44466796	0.42918722	0.44344888	0.38309229	0	0.36119526	0.60219807	0.00319954	1.54934863	0.4339398	0.07625167	0.55672688
RAD	1.0114492	0.8464319	0.07628758	0.8500034	0.06896034	0.19769629	0.85390635	1.03025979	0.19691335	0.98002225	0.41350308	0.18930038	0.00919911	0.09439845	0.41793849	0.43145652	0.38639557	0.02446422	0.03218426	0.21737602	0.12622221	0.80586321	0.06799196	0.08225362	0.02189703	0.36119526	0	0.24100281	0.35799572	1.91054388	0.07274454	0.43744693	0.19553163
STR	1.25245201	1.08743471	0.31729039	1.09100621	0.30996315	0.4386991	1.09490916	1.2712626	0.04408946	1.22102506	0.65450589	0.05170243	0.25020192	0.14660436	0.6589413	0.67245933	0.62739838	0.26546703	0.20881855	0.45837883	0.1147806	1.04686602	0.17301085	0.15874919	0.21910578	0.60219807	0.24100281	0	0.59899853	2.15154669	0.16825826	0.67844973	0.04547118
TAN	0.65345348	0.48843618	0.28170814	0.49200769	0.28903538	0.16029943	0.49591063	0.67226407	0.55490907	0.62202653	0.05550736	0.54729609	0.34879661	0.45239417	0.05994277	0.0734608	0.02839985	0.3335315	0.39017998	0.1406197	0.48421793	0.4478675	0.42598768	0.44024934	0.37989275	0.00319954	0.35799572	0.59899853	0	1.55254816	0.43074026	0.07945121	0.55352735
VAL	0.89909468	1.06411198	1.8342563	1.06054048	1.84158354	1.71284759	1.05663753	0.88028409	2.10745723	0.93052163	1.4970408	2.09984426	1.90134477	2.00494233	1.49260539	1.47908736	1.52414831	1.88607966	1.94272815	1.69316786	2.03676609	1.10468067	1.97853584	1.9927975	1.93244091	1.54934863	1.91054388	2.15154669	1.55254816	0	1.98328843	1.47309696	2.10607551
VRE	1.08419375	0.91917644	0.14903213	0.92274795	0.14170489	0.27044084	0.9266509	1.10300433	0.12416881	1.0527668	0.48624762	0.11655583	0.08194365	0.0216539	0.49068303	0.50420107	0.45914012	0.09720876	0.04056028	0.29012056	0.05347766	0.87860776	0.00475258	0.00950908	0.05084752	0.4339398	0.07274454	0.16825826	0.43074026	1.98328843	0	0.51019147	0.12278708
ZAG	0.57400228	0.40898497	0.36115934	0.41255648	0.36848658	0.23975063	0.41645943	0.59281286	0.63436028	0.54257533	0.02394385	0.6267473	0.42824782	0.53184537	0.01950844	0.0059904	0.05105135	0.41298271	0.46963119	0.22007091	0.56366913	0.36841629	0.50543889	0.51970055	0.45934395	0.07625167	0.43744693	0.67844973	0.07945121	1.47309696	0.51019147	0	0.63297855
ZAL	1.20698083	1.04196353	0.27181921	1.04553503	0.26449197	0.39322792	1.04943798	1.22579142	0.00138172	1.17555388	0.60903471	0.00623125	0.20473074	0.10113318	0.61347012	0.62698815	0.5819272	0.21999584	0.16334736	0.41290765	0.06930942	1.00139484	0.12753967	0.113278	0.1736346	0.55672688	0.19553163	0.04547118	0.55352735	2.10607551	0.12278708	0.63297855	0

e) Climatic niche

Locality	BAL	BAZ	CAV	DOS	EBE	FAL	FED	FEN	FUR	GAV	GNI	GOS	HIR	HOD	HUS	CHAS	JAK	KAM	KOL	LAG	LOI	LOR	LYS	MAR	MIS	OMS	PIT	POD	PON	RAD	STR	TAN	TRD	VAL	VOD	VRE	ZAG	ZAL
BAL	0	0.04845916	2.10174385	1.32775319	2.11916769	0.61766536	0.4496123	0.22923867	2.37681532	0.55083474	1.44017551	2.70093031	1.93686513	2.09674089	2.80436343	0.55834193	1.8666846	2.90103793	1.51311875	1.7227167	1.58637363	1.88623	1.77432355	2.19646875	0.40834968	1.97300865	2.53688303	2.54022313	2.13226813	2.26007241	1.76224814	1.84871337	1.30053184	0.99803332	1.79054541	1.60268237	2.66020896	2.7174979
BAZ	0.04845916	0	2.06197789	1.28920882	2.07933517	0.61177611	0.47249037	0.19055498	2.337667	0.5120165	1.40210482	2.66275202	1.89727365	2.05742504	2.76627279	0.58394135	1.82765704	2.86203393	1.47750901	1.68367358	1.54814965	1.84733277	1.73464455	2.15706277	0.39953857	1.93368931	2.49823208	2.50090706	2.09276082	2.22121327	1.72295284	1.81006018	1.26211664	0.96152001	1.7519535	1.56554941	2.62109731	2.67940525
CAV	2.10174385	2.06197789	0	0.77979418	0.52515717	2.25220251	2.47287594	1.87433967	0.29110826	1.55141195	1.0193214	0.647254	0.62792358	0.07697136	0.75410595	2.58921966	0.65422164	1.05787382	1.2225841	0.79479507	0.959678	0.68632282	0.3277691	0.10551019	2.16620704	0.15073706	0.46917301	0.47142649	0.55672256	0.2285857	0.35214033	0.75688518	1.0758997	1.13709475	0.76264567	1.0154497	0.85458079	0.67139014
DOS	1.32775319	1.28920882	0.77979418	0	0.93692461	1.55134948	1.71752065	1.10262846	1.04948081	0.77723863	0.69331905	1.37620299	0.82872679	0.77002973	1.48095687	1.83462456	0.77846606	1.70012605	0.93796337	0.74391272	0.7602369	0.81585409	0.45311855	0.87080657	1.4348061	0.64640057	1.20988057	1.23208809	0.96053958	0.93358236	0.43597648	0.82761806	0.63646961	0.36449472	0.78009637	0.82011413	1.46306948	1.39408706
EBE	2.11916769	2.07933517	0.52515717	0.93692461	0	2.12581083	2.42954194	1.89211459	0.69768088	1.59821701	0.76348948	1.01274343	0.21071865	0.59930141	1.10752537	2.54094058	0.28065005	0.78819631	0.88354924	0.45786009	0.64419045	0.28298684	0.60516493	0.58386266	2.08875625	0.60330786	0.85357686	0.59505194	0.03773195	0.71963369	0.67532323	0.3533795	0.87636925	1.28489983	0.39462107	0.67629781	0.54672723	1.04313384
FAL	0.61766536	0.61177611	2.25220251	1.55134948	2.12581083	0	0.45780242	0.65625368	2.54242993	0.88389235	1.37108935	2.8923993	1.9207806	2.26820778	2.99941318	0.5220656	1.85269077	2.86898463	1.3414785	1.67870095	1.50553806	1.85967486	1.94353868	2.35568073	0.21487927	2.15034699	2.71713255	2.64109456	2.13166469	2.44790478	1.95444315	1.80296856	1.25177767	1.31744531	1.75055467	1.50056058	2.63782097	2.91390007
FED	0.4496123	0.47249037	2.47287594	1.71752065	2.42954194	0.45780242	0	0.63480759	2.756116	0.95274211	1.70189374	3.09175897	2.23413584	2.47591926	3.19676798	0.11713979	2.16471824	3.19913835	1.71855967	2.00294286	1.84481737	2.17824891	2.14937493	2.57203621	0.35946877	2.35321817	2.92267131	2.89661779	2.43966391	2.64638029	2.14555429	2.13040053	1.56909665	1.4126687	2.07444345	1.85026475	2.9616293	3.10999206
FEN	0.22923867	0.19055498	1.87433967	1.10262846	1.89211459	0.65625368	0.63480759	0	2.15085906	0.32633826	1.22559094	2.47775521	1.71193351	1.87061595	2.58174962	0.75044204	1.64221736	2.67517815	1.31542284	1.50168205	1.37133353	1.66274887	1.54721477	1.96973059	0.45778225	1.74699118	2.31237696	2.31145768	1.90573189	2.03565574	1.53671968	1.62730084	1.08519872	0.78263392	1.56898883	1.39123658	2.43407898	2.49485357
FUR	2.37681532	2.337667	0.29110826	1.04948081	0.69768088	2.54242993	2.756116	2.15085906	0	1.82601276	1.29729309	0.35814659	0.85264929	0.28055311	0.46445943	2.87275063	0.89225114	0.98914228	1.48875609	1.05170051	1.22638441	0.9181559	0.60681752	0.18676397	2.45377632	0.40410991	0.17983462	0.31751457	0.72308899	0.14081981	0.61510491	0.99298786	1.36125945	1.39713991	1.00902534	1.27738204	0.83536356	0.38368276
GAV	0.55083474	0.5120165	1.55141195	0.77723863	1.59821701	0.88389235	0.95274211	0.32633826	1.82601276	0	0.98718968	2.15164425	1.42887611	1.54590951	2.25554319	1.06951671	1.36124991	2.38390901	1.12364574	1.23584258	1.12811835	1.38565445	1.22382326	1.64574219	0.71743285	1.42217603	1.98661751	1.99350771	1.61415619	1.70979239	1.21147229	1.35841323	0.8486379	0.4612713	1.30010317	1.15854782	2.14228225	2.16865089
GNI	1.44017551	1.40210482	1.0193214	0.69331905	0.76348948	1.37108935	1.70189374	1.22559094	1.29729309	0.98718968	0	1.65195878	0.55473923	1.06219465	1.75761511	1.80905325	0.4855679	1.50228383	0.248118	0.30952909	0.14633417	0.4895516	0.79801838	1.12059935	1.35154253	0.97248289	1.47563475	1.31694552	0.76538083	1.2432714	0.84858391	0.43263986	0.14104007	0.86906112	0.37956687	0.17383788	1.26868019	1.67871011
GOS	2.70093031	2.66275202	0.647254	1.37620299	1.01274343	2.8923993	3.09175897	2.47775521	0.35814659	2.15164425	1.65195878	0	1.18798703	0.62452219	0.10751456	3.20871915	1.23244232	1.07008995	1.83829411	1.39920904	1.57717911	1.25496551	0.94977878	0.54194765	2.79751971	0.74313576	0.17841673	0.46635497	1.03295295	0.4467825	0.94675942	1.3307478	1.71745303	1.70886921	1.35149869	1.62526654	0.98942499	0.03124388
HIR	1.93686513	1.89727365	0.62792358	0.82872679	0.21071865	1.9207806	2.23413584	1.71193351	0.85264929	1.42887611	0.55473923	1.18798703	0	0.69500219	1.28732586	2.34434288	0.0794983	0.96782123	0.6759748	0.24736833	0.43349097	0.07968588	0.59371781	0.70891713	1.88981704	0.66126384	1.020541	0.79436477	0.21176839	0.84628869	0.6664085	0.14986581	0.67217028	1.15375638	0.18616906	0.46737015	0.72845797	1.21751331
HOD	2.09674089	2.05742504	0.07697136	0.77002973	0.59930141	2.26820778	2.47591926	1.87061595	0.28055311	1.54590951	1.06219465	0.62452219	0.69500219	0	0.73144376	2.59260387	0.71662734	1.11848681	1.27257931	0.85109749	1.01049301	0.75017337	0.32721383	0.10552663	2.17559593	0.1238338	0.44953705	0.50766939	0.6304141	0.18419623	0.33547492	0.81938529	1.11107927	1.12060671	0.82184953	1.06743553	0.92044484	0.64641017
HUS	2.80436343	2.76627279	0.75410595	1.48095687	1.10752537	2.99941318	3.19676798	2.58174962	0.46445943	2.25554319	1.75761511	0.10751456	1.28732586	0.73144376	0	3.31374723	1.33354242	1.10522297	1.94170621	1.50196976	1.68104591	1.35504631	1.05645766	0.64892667	2.90377832	0.84969106	0.28503742	0.54298241	1.12665555	0.5527337	1.05236585	1.43092374	1.8242308	1.81085779	1.45312895	1.72845478	1.04589848	0.08689983
CHAS	0.55834193	0.58394135	2.58921966	1.83462456	2.54094058	0.5220656	0.11713979	0.75044204	2.87275063	1.06951671	1.80905325	3.20871915	2.34434288	2.59260387	3.31374723	0	2.27497603	3.30823254	1.81804471	2.11157195	1.95116678	2.2877986	2.26599373	2.68855663	0.45981745	2.46996588	3.03950972	3.0118653	2.55066427	2.76325497	2.26243513	2.23881868	1.67758346	1.5292064	2.18320527	1.95497822	3.07134174	3.22698009
JAK	1.8666846	1.82765704	0.65422164	0.77846606	0.28065005	1.85269077	2.16471824	1.64221736	0.89225114	1.36124991	0.4855679	1.23244232	0.0794983	0.71662734	1.33354242	2.27497603	0	1.03678808	0.6169885	0.18324031	0.36918551	0.04119248	0.58235211	0.74010834	1.82091938	0.67105045	1.06288778	0.85106652	0.28117591	0.87576206	0.65359153	0.10311401	0.60200921	1.09483553	0.12100934	0.40443781	0.79766242	1.26154982
KAM	2.90103793	2.86203393	1.05787382	1.70012605	0.78819631	2.86898463	3.19913835	2.67517815	0.98914228	2.38390901	1.50228383	1.07008995	0.96782123	1.11848681	1.10522297	3.30823254	1.03678808	0	1.55055464	1.19679322	1.36408104	1.0213198	1.30284067	1.03299547	2.85168421	1.19907956	1.01892609	0.67491554	0.77102335	1.10782913	1.35894285	1.07028294	1.63097582	2.05757116	1.12565144	1.37226412	0.2417246	1.09683516
KOL	1.51311875	1.47750901	1.2225841	0.93796337	0.88354924	1.3414785	1.71855967	1.31542284	1.48875609	1.12364574	0.248118	1.83829411	0.6759748	1.27257931	1.94170621	1.81804471	0.6169885	1.55055464	0	0.44124143	0.26305142	0.60481419	1.03043582	1.3202928	1.36110788	1.19277124	1.66468978	1.46784049	0.87694545	1.44915309	1.0853468	0.53188793	0.32205993	1.082147	0.49637144	0.21478866	1.3310205	1.86642345
LAG	1.7227167	1.68367358	0.79479507	0.74391272	0.45786009	1.67870095	2.00294286	1.50168205	1.05170051	1.23584258	0.30952909	1.39920904	0.24736833	0.85109749	1.50196976	2.11157195	0.18324031	1.19679322	0.44124143	0	0.18747792	0.18254821	0.65735093	0.88872881	1.65494845	0.78641375	1.22641971	1.03040537	0.45791811	1.02135774	0.72356661	0.1298119	0.4345908	1.0202232	0.07754386	0.23055778	0.96137351	1.42762919
LOI	1.58637363	1.54814965	0.959678	0.7602369	0.64419045	1.50553806	1.84481737	1.37133353	1.22638441	1.12811835	0.14633417	1.57717911	0.43349097	1.01049301	1.68104591	1.95116678	0.36918551	1.36408104	0.26305142	0.18747792	0	0.36522007	0.7796181	1.05734417	1.4927863	0.93355725	1.40287117	1.2168923	0.64259711	1.18645946	0.83873384	0.30047379	0.28624662	0.97912484	0.25363765	0.0725242	1.13299138	1.60507976
LOR	1.88623	1.84733277	0.68632282	0.81585409	0.28298684	1.85967486	2.17824891	1.66274887	0.9181559	1.38565445	0.4895516	1.25496551	0.07968588	0.75017337	1.35504631	2.2877986	0.04119248	1.0213198	0.60481419	0.18254821	0.36522007	0	0.62291043	0.77003071	1.8325384	0.70801818	1.08698311	0.8642045	0.27884973	0.90633253	0.69430997	0.07592628	0.61160069	1.12813488	0.11244394	0.39465851	0.78390732	1.28437041
LYS	1.77432355	1.73464455	0.3277691	0.45311855	0.60516493	1.94353868	2.14937493	1.54721477	0.60681752	1.22382326	0.79801838	0.94977878	0.59371781	0.32721383	1.05645766	2.26599373	0.58235211	1.30284067	1.03043582	0.65735093	0.7796181	0.62291043	0	0.42314638	1.84858283	0.20686793	0.77627452	0.78551019	0.63607381	0.50443685	0.07311204	0.67344378	0.82171516	0.81288805	0.65361065	0.84298609	1.07618726	0.97064759
MAR	2.19646875	2.15706277	0.10551019	0.87080657	0.58386266	2.35568073	2.57203621	1.96973059	0.18676397	1.64574219	1.12059935	0.54194765	0.70891713	0.10552663	0.64892667	2.68855663	0.74010834	1.03299547	1.3202928	0.88872881	1.05734417	0.77003071	0.42314638	0	2.26782023	0.22670452	0.36399652	0.4056199	0.61319322	0.13791613	0.43800613	0.84265539	1.17930685	1.22390062	0.85222461	1.11125424	0.84510902	0.56615298
MIS	0.40834968	0.39953857	2.16620704	1.4348061	2.08875625	0.21487927	0.35946877	0.45778225	2.45377632	0.71743285	1.35154253	2.79751971	1.88981704	2.17559593	2.90377832	0.45981745	1.82091938	2.85168421	1.36110788	1.65494845	1.4927863	1.8325384	1.84858283	2.26782023	0	2.05482997	2.62485413	2.57554524	2.09762207	2.35128736	1.85217819	1.78211794	1.22151896	1.1685754	1.72705393	1.49666467	2.6157125	2.81745926
OMS	1.97300865	1.93368931	0.15073706	0.64640057	0.60330786	2.15034699	2.35321817	1.74699118	0.40410991	1.42217603	0.97248289	0.74313576	0.66126384	0.1238338	0.84969106	2.46996588	0.67105045	1.19907956	1.19277124	0.78641375	0.93355725	0.70801818	0.20686793	0.22670452	2.05482997	0	0.57027236	0.61785296	0.63532086	0.29759341	0.21174274	0.7717713	1.01112893	0.99760432	0.76597949	0.99329084	0.98890615	0.76386209
PIT	2.53688303	2.49823208	0.46917301	1.20988057	0.85357686	2.71713255	2.92267131	2.31237696	0.17983462	1.98661751	1.47563475	0.17841673	1.020541	0.44953705	0.28503742	3.03950972	1.06288778	1.01892609	1.66468978	1.22641971	1.40287117	1.08698311	0.77627452	0.36399652	2.62485413	0.57027236	0	0.36055664	0.8761476	0.2776346	0.77731354	1.16244176	1.54012116	1.5493811	1.18103404	1.45239703	0.90202221	0.20395321
POD	2.54022313	2.50090706	0.47142649	1.23208809	0.59505194	2.64109456	2.89661779	2.31145768	0.31751457	1.99350771	1.31694552	0.46635497	0.79436477	0.50766939	0.54298241	3.0118653	0.85106652	0.67491554	1.46784049	1.03040537	1.2168923	0.8642045	0.78551019	0.4056199	2.57554524	0.61785296	0.36055664	0	0.60814165	0.44528503	0.81855675	0.93893194	1.40841555	1.59398795	0.97167708	1.25537386	0.54183231	0.49735864
PON	2.13226813	2.09276082	0.55672256	0.96053958	0.03773195	2.13166469	2.43966391	1.90573189	0.72308899	1.61415619	0.76538083	1.03295295	0.21176839	0.6304141	1.12665555	2.55066427	0.28117591	0.77102335	0.87694545	0.45791811	0.64259711	0.27884973	0.63607381	0.61319322	2.09762207	0.63532086	0.8761476	0.60814165	0	0.74818866	0.70626411	0.34672567	0.88163485	1.30626423	0.39124382	0.67094528	0.52947716	1.06353684
RAD	2.26007241	2.22121327	0.2285857	0.93358236	0.71963369	2.44790478	2.64638029	2.03565574	0.14081981	1.70979239	1.2432714	0.4467825	0.84628869	0.18419623	0.5527337	2.76325497	0.87576206	1.10782913	1.44915309	1.02135774	1.18645946	0.90633253	0.50443685	0.13791613	2.35128736	0.29759341	0.2776346	0.44528503	0.74818866	0	0.50090194	0.97857195	1.29467081	1.27351494	0.98654965	1.24143782	0.9379935	0.4667552
STR	1.76224814	1.72295284	0.35214033	0.43597648	0.67532323	1.95444315	2.14555429	1.53671968	0.61510491	1.21147229	0.84858391	0.94675942	0.6664085	0.33547492	1.05236585	2.26243513	0.65359153	1.35894285	1.0853468	0.72356661	0.83873384	0.69430997	0.07311204	0.43800613	1.85217819	0.21174274	0.77731354	0.81855675	0.70626411	0.50090194	0	0.74367736	0.86291341	0.78682487	0.72213938	0.90248981	1.13547452	0.9658251
TAN	1.84871337	1.81006018	0.75688518	0.82761806	0.3533795	1.80296856	2.13040053	1.62730084	0.99298786	1.35841323	0.43263986	1.3307478	0.14986581	0.81938529	1.43092374	2.23881868	0.10311401	1.07028294	0.53188793	0.1298119	0.30047379	0.07592628	0.67344378	0.84265539	1.78211794	0.7717713	1.16244176	0.93893194	0.34672567	0.97857195	0.74367736	0	0.56132512	1.12418688	0.05869344	0.32425209	0.83609486	1.36011281
TRD	1.30053184	1.26211664	1.0758997	0.63646961	0.87636925	1.25177767	1.56909665	1.08519872	1.36125945	0.8486379	0.14104007	1.71745303	0.67217028	1.11107927	1.8242308	1.67758346	0.60200921	1.63097582	0.32205993	0.4345908	0.28624662	0.61160069	0.82171516	1.17930685	1.22151896	1.01112893	1.54012116	1.40841555	0.88163485	1.29467081	0.86291341	0.56132512	0	0.7616812	0.50566175	0.31237703	1.39498398	1.7429424
VAL	0.99803332	0.96152001	1.13709475	0.36449472	1.28489983	1.31744531	1.4126687	0.78263392	1.39713991	0.4612713	0.86906112	1.70886921	1.15375638	1.12060671	1.81085779	1.5292064	1.09483553	2.05757116	1.082147	1.0202232	0.97912484	1.12813488	0.81288805	1.22390062	1.1685754	0.99760432	1.5493811	1.59398795	1.30626423	1.27351494	0.78682487	1.12418688	0.7616812	0	1.07007324	1.02854737	1.81884922	1.72417718
VOD	1.79054541	1.7519535	0.76264567	0.78009637	0.39462107	1.75055467	2.07444345	1.56898883	1.00902534	1.30010317	0.37956687	1.35149869	0.18616906	0.82184953	1.45312895	2.18320527	0.12100934	1.12565144	0.49637144	0.07754386	0.25363765	0.11244394	0.65361065	0.85222461	1.72705393	0.76597949	1.18103404	0.97167708	0.39124382	0.98654965	0.72213938	0.05869344	0.50566175	1.07007324	0	0.2841407	0.89032087	1.38041893
VRE	1.60268237	1.56554941	1.0154497	0.82011413	0.67629781	1.50056058	1.85026475	1.39123658	1.27738204	1.15854782	0.17383788	1.62526654	0.46737015	1.06743553	1.72845478	1.95497822	0.40443781	1.37226412	0.21478866	0.23055778	0.0725242	0.39465851	0.84298609	1.11125424	1.49666467	0.99329084	1.45239703	1.25537386	0.67094528	1.24143782	0.90248981	0.32425209	0.31237703	1.02854737	0.2841407	0	1.14516075	1.65354815
ZAG	2.66020896	2.62109731	0.85458079	1.46306948	0.54672723	2.63782097	2.9616293	2.43407898	0.83536356	2.14228225	1.26868019	0.98942499	0.72845797	0.92044484	1.04589848	3.07134174	0.79766242	0.2417246	1.3310205	0.96137351	1.13299138	0.78390732	1.07618726	0.84510902	2.6157125	0.98890615	0.90202221	0.54183231	0.52947716	0.9379935	1.13547452	0.83609486	1.39498398	1.81884922	0.89032087	1.14516075	0	1.01902968
ZAL	2.7174979	2.67940525	0.67139014	1.39408706	1.04313384	2.91390007	3.10999206	2.49485357	0.38368276	2.16865089	1.67871011	0.03124388	1.21751331	0.64641017	0.08689983	3.22698009	1.26154982	1.09683516	1.86642345	1.42762919	1.60507976	1.28437041	0.97064759	0.56615298	2.81745926	0.76386209	0.20395321	0.49735864	1.06353684	0.4667552	0.9658251	1.36011281	1.7429424	1.72417718	1.38041893	1.65354815	1.01902968	0

f) Topographic niche

Locality	BAL	BAZ	CAV	DOS	EBE	FAL	FEN	GAV	GNI	GOS	HIR	HOD	HUS	JAK	KAM	KOL	LAG	LOI	LOR	LYS	MAR	MIS	OMS	PIT	POD	PON	RAD	STR	TAN	TRD	VAL	VOD	VRE	ZAG	ZAL
BAL	0	0.22924893	1.17542681	0.36400118	1.37134788	0.54819315	1.21844049	0.98796588	0.76390827	1.90743597	1.07122632	1.23692194	2.15583641	1.02533449	2.37844301	0.86190464	1.70337196	0.91303983	1.02955906	0.86409612	1.44611379	1.67663165	1.444754	1.48342946	1.19571954	1.73405425	1.28618817	0.97864787	1.00356029	1.28712006	1.08523139	1.15203636	0.82274898	1.76265977	1.77392168
BAZ	0.22924893	0	1.31205362	0.56818656	1.5272375	0.69454755	1.01830026	0.79755397	0.80500404	1.97411551	1.20622493	1.31826192	2.30875062	1.16081471	2.36746543	0.74264178	1.54245138	0.87724059	1.04921112	1.01580583	1.45350886	1.45324729	1.3918269	1.66491341	1.38427108	1.87992157	1.38167013	1.05209017	0.93562395	1.11575062	0.92994496	1.05824293	0.85973398	1.94001461	1.92444576
CAV	1.17542681	1.31205362	0	0.88667258	0.48136793	1.47630803	1.91875119	1.68529783	0.68222702	1.3241407	0.33884947	0.35664194	1.1184995	0.40096939	2.03376048	1.29464195	1.92934075	0.88893497	0.65306992	0.33283735	0.89327557	2.49010436	1.18688955	0.55754093	0.80560048	1.08622442	0.30002923	0.42825474	1.1729203	1.71774548	1.4537529	1.24750578	0.66301519	0.71335589	0.97107482
DOS	0.36400118	0.56818656	0.88667258	0	1.11000199	0.67487613	1.44119443	1.19822037	0.69360785	1.66013702	0.83942455	1.01079567	1.81080799	0.80671537	2.23497239	0.95763916	1.82203936	0.90105247	0.91852818	0.58814694	1.30006183	1.96145397	1.3914691	1.12960596	0.84054678	1.54553652	1.03621545	0.8043192	1.00204058	1.43927471	1.22776543	1.18077389	0.74384411	1.4274884	1.43185113
EBE	1.37134788	1.5272375	0.48136793	1.11000199	0	1.53703268	2.26069791	2.02368361	0.90412921	1.7692667	0.35166171	0.76050543	1.28900645	0.38543462	2.50058619	1.66523557	2.28641138	1.19894428	0.99008729	0.56686194	1.31029759	2.79132979	1.58458631	0.69446222	1.07398976	0.61413485	0.71496835	0.73497363	1.58955476	2.07565971	1.76919985	1.63281686	0.89649476	0.62849768	1.28234689
FAL	0.54819315	0.69454755	1.47630803	0.67487613	1.53703268	0	1.67917565	1.47205292	1.19323821	2.31060951	1.32462261	1.63954367	2.39441259	1.26879993	2.85248484	1.40421989	2.23425505	1.42231663	1.50215234	1.15039922	1.94258667	2.04768706	1.97981962	1.65792823	1.35180124	1.77587896	1.66676714	1.38467111	1.54501442	1.80852516	1.61134497	1.69957804	1.2558935	1.91278998	2.02583368
FEN	1.21844049	1.01830026	1.91875119	1.44119443	2.26069791	1.67917565	0	0.2465562	1.37805735	2.04326681	1.91044696	1.7363465	2.77309412	1.88832485	2.03212998	0.63233626	0.81206574	1.13495715	1.38397077	1.74042335	1.51112809	0.6155404	1.22474215	2.32908013	2.04988465	2.67484411	1.8252011	1.56848747	0.84402506	0.41643585	0.63834434	0.8489869	1.39232938	2.61413123	2.37301839
GAV	0.98796588	0.79755397	1.68529783	1.19822037	2.02368361	1.47205292	0.2465562	0	1.14942271	1.89349687	1.6740771	1.52111765	2.5628106	1.651305	1.97960391	0.41810969	0.87172454	0.92915227	1.17444611	1.49781816	1.34584648	0.83084736	1.09862278	2.09321695	1.818934	2.44477202	1.60777785	1.34254927	0.66154324	0.41615298	0.51254321	0.70301129	1.16697907	2.37871909	2.16451407
GNI	0.76390827	0.80500404	0.68222702	0.69360785	0.90412921	1.19323821	1.37805735	1.14942271	0	1.64009738	0.55522697	0.6158403	1.77241644	0.53034885	2.13848371	0.822873	1.4614729	0.36330886	0.3569471	0.49685142	0.88830471	1.8928675	0.95617776	1.20014695	1.20540507	1.31258526	0.69477416	0.30107785	0.82993236	1.20278096	0.88224863	0.83647119	0.06381512	1.37398165	1.53835731
GOS	1.90743597	1.97411551	1.3241407	1.66013702	1.7692667	2.31060951	2.04326681	1.89349687	1.64009738	0	1.64505831	1.19243554	1.13252605	1.69654688	0.86675654	1.54085875	1.91934479	1.53271057	1.38478921	1.49881937	1.00366621	2.64162753	1.23659629	1.30909721	1.23176387	2.37451015	1.16187203	1.43313705	1.28022883	1.82368875	1.80416131	1.42312228	1.61070961	1.5566941	0.81293235
HIR	1.07122632	1.20622493	0.33884947	0.83942455	0.35166171	1.32462261	1.91044696	1.6740771	0.55522697	1.64505831	0	0.55133361	1.41339145	0.07161431	2.31306634	1.32131011	1.96219791	0.86146688	0.67806708	0.28376452	1.07791419	2.44008876	1.30499813	0.78702372	1.00342189	0.85509209	0.54755455	0.41463589	1.26385761	1.73200492	1.42484422	1.30603704	0.55095944	0.8698133	1.29092402
HOD	1.23692194	1.31826192	0.35664194	1.01079567	0.76050543	1.63954367	1.7363465	1.52111765	0.6158403	1.19243554	0.55133361	0	1.22152281	0.60306442	1.79933345	1.11224906	1.63212997	0.67630172	0.40541193	0.54639595	0.55239506	2.30988392	0.85315036	0.84481407	1.02986171	1.31462032	0.10622377	0.32177662	0.95461549	1.47774443	1.22593134	0.97812779	0.56998106	0.98458631	1.07408482
HUS	2.15583641	2.30875062	1.1184995	1.81080799	1.28900645	2.39441259	2.77309412	2.5628106	1.77241644	1.13252605	1.41339145	1.22152281	0	1.47923249	1.95122747	2.15605296	2.67688023	1.87137349	1.61615634	1.40290913	1.46795957	3.38398176	1.84382118	0.76619466	1.13326496	1.76799995	1.12001806	1.48990723	1.94109157	2.54520668	2.37319289	2.04985496	1.74459752	0.74710286	0.54883701
JAK	1.02533449	1.16081471	0.40096939	0.80671537	0.38543462	1.26879993	1.88832485	1.651305	0.53034885	1.69654688	0.07161431	0.60306442	1.47923249	0	2.35631482	1.30946769	1.96062317	0.85478466	0.69119136	0.28452506	1.11698369	2.40954826	1.32757898	0.83675666	1.02591842	0.84764005	0.6067739	0.43093704	1.26690729	1.72062821	1.40893657	1.30892125	0.5329319	0.92461103	1.34582904
KAM	2.37844301	2.36746543	2.03376048	2.23497239	2.50058619	2.85248484	2.03212998	1.97960391	2.13848371	0.86675654	2.31306634	1.79933345	1.95122747	2.35631482	0	1.74076789	1.75204732	1.89909914	1.8429891	2.16626261	1.36742496	2.53471686	1.38422965	2.14302872	2.0145314	3.09194095	1.8093513	1.99563013	1.49843932	1.80164007	1.9291805	1.5723731	2.10516902	2.38212969	1.67919527
KOL	0.86190464	0.74264178	1.29464195	0.95763916	1.66523557	1.40421989	0.63233626	0.41810969	0.822873	1.54085875	1.32131011	1.11224906	2.15605296	1.30946769	1.74076789	0	0.88562332	0.57632204	0.78720534	1.14578153	0.94854459	1.23131262	0.75944731	1.71134932	1.48862264	2.13097558	1.19736224	0.96360318	0.27423211	0.49651656	0.40732701	0.37822399	0.82941426	1.98816062	1.78157327
LAG	1.70337196	1.54245138	1.92934075	1.82203936	2.28641138	2.23425505	0.81206574	0.87172454	1.4614729	1.91934479	1.96219791	1.63212997	2.67688023	1.96062317	1.75204732	0.88562332	0	1.10811773	1.29950676	1.87099803	1.22748486	1.11961517	0.84740107	2.39758337	2.27861841	2.70149804	1.72885699	1.55771229	0.91067998	0.45864812	0.64839308	0.70334248	1.44028721	2.61112957	2.39008444
LOI	0.91303983	0.87724059	0.88893497	0.90105247	1.19894428	1.42231663	1.13495715	0.92915227	0.36330886	1.53271057	0.86146688	0.67630172	1.87137349	0.85478466	1.89909914	0.57632204	1.10811773	0	0.27541086	0.78634302	0.68334622	1.66671362	0.63340746	1.40772731	1.3775083	1.63005081	0.77778155	0.47771173	0.55833524	0.88817851	0.5786248	0.48883099	0.33549411	1.59928626	1.61695241
LOR	1.02955906	1.04921112	0.65306992	0.91852818	0.99008729	1.50215234	1.38397077	1.17444611	0.3569471	1.38478921	0.67806708	0.40541193	1.61615634	0.69119136	1.8429891	0.78720534	1.29950676	0.27541086	0	0.63486759	0.5378668	1.93317907	0.64580568	1.18285944	1.23483675	1.46512631	0.50921408	0.26618047	0.68864686	1.12189743	0.83723243	0.65458196	0.30123589	1.35073958	1.40501401
LYS	0.86409612	1.01580583	0.33283735	0.58814694	0.56686194	1.15039922	1.74042335	1.49781816	0.49685142	1.49881937	0.28376452	0.54639595	1.40290913	0.28452506	2.16626261	1.14578153	1.87099803	0.78634302	0.63486759	0	1.02199696	2.2904238	1.23399399	0.73596609	0.78897064	1.0971251	0.54736016	0.39244401	1.08911692	1.5968104	1.32049629	1.18592959	0.50898635	0.94188152	1.17173779
MAR	1.44611379	1.45350886	0.89327557	1.30006183	1.31029759	1.94258667	1.51112809	1.34584648	0.88830471	1.00366621	1.07791419	0.55239506	1.46795957	1.11698369	1.36742496	0.94854459	1.22748486	0.68334622	0.5378668	1.02199696	0	2.08173243	0.38133689	1.29480055	1.36104608	1.83819072	0.61494563	0.71530923	0.71761737	1.17919244	1.02364692	0.67392738	0.83657562	1.46370954	1.27774743
MIS	1.67663165	1.45324729	2.49010436	1.96145397	2.79132979	2.04768706	0.6155404	0.83084736	1.8928675	2.64162753	2.44008876	2.30988392	3.38398176	2.40954826	2.53471686	1.23131262	1.11961517	1.66671362	1.93317907	2.2904238	2.08173243	0	1.76161103	2.91868589	2.63475616	3.14183914	2.4037779	2.11646477	1.45073947	0.91102165	1.12090859	1.40874541	1.91079661	3.19313596	2.98713848
OMS	1.444754	1.3918269	1.18688955	1.3914691	1.58458631	1.97981962	1.22474215	1.09862278	0.95617776	1.23659629	1.30499813	0.85315036	1.84382118	1.32757898	1.38422965	0.75944731	0.84740107	0.63340746	0.64580568	1.23399399	0.38133689	1.76161103	0	1.62916499	1.6200019	2.07330237	0.9379218	0.89751007	0.5637861	0.85166311	0.74352013	0.40054904	0.91134171	1.81558472	1.6147924
PIT	1.48342946	1.66491341	0.55754093	1.12960596	0.69446222	1.65792823	2.32908013	2.09321695	1.20014695	1.30909721	0.78702372	0.84481407	0.76619466	0.83675666	2.14302872	1.71134932	2.39758337	1.40772731	1.18285944	0.73596609	1.29480055	2.91868589	1.62916499	0	0.57180261	1.2379495	0.75148414	0.97517367	1.569088	2.16618355	1.9425626	1.70337906	1.19305727	0.39006452	0.63850247
POD	1.19571954	1.38427108	0.80560048	0.84054678	1.07398976	1.35180124	2.04988465	1.818934	1.20540507	1.23176387	1.00342189	1.02986171	1.13326496	1.02591842	2.0145314	1.48862264	2.27861841	1.3775083	1.23483675	0.78897064	1.36104608	2.63475616	1.6200019	0.57180261	0	1.61964019	0.97451215	1.07460442	1.37570055	1.97299286	1.80900377	1.58795205	1.21832338	0.9554337	0.70417581
PON	1.73405425	1.87992157	1.08622442	1.54553652	0.61413485	1.77587896	2.67484411	2.44477202	1.31258526	2.37451015	0.85509209	1.31462032	1.76799995	0.84764005	3.09194095	2.13097558	2.70149804	1.63005081	1.46512631	1.0971251	1.83819072	3.14183914	2.07330237	1.2379495	1.61964019	0	1.28665009	1.23069223	2.10100514	2.50358518	2.1635647	2.1045639	1.31256607	1.04874655	1.85327544
RAD	1.28618817	1.38167013	0.30002923	1.03621545	0.71496835	1.66676714	1.8252011	1.60777785	0.69477416	1.16187203	0.54755455	0.10622377	1.12001806	0.6067739	1.8093513	1.19736224	1.72885699	0.77778155	0.50921408	0.54736016	0.61494563	2.4037779	0.9379218	0.75148414	0.97451215	1.28665009	0	0.39647938	1.0336061	1.5726991	1.32677297	1.0718644	0.65331172	0.88646413	0.98829933
STR	0.97864787	1.05209017	0.42825474	0.8043192	0.73497363	1.38467111	1.56848747	1.34254927	0.30107785	1.43313705	0.41463589	0.32177662	1.48990723	0.43093704	1.99563013	0.96360318	1.55771229	0.47771173	0.26618047	0.39244401	0.71530923	2.11646477	0.89751007	0.97517367	1.07460442	1.23069223	0.39647938	0	0.88217886	1.3484734	1.05424465	0.89893992	0.26035357	1.13095962	1.29802321
TAN	1.00356029	0.93562395	1.1729203	1.00204058	1.58955476	1.54501442	0.84402506	0.66154324	0.82993236	1.28022883	1.26385761	0.95461549	1.94109157	1.26690729	1.49843932	0.27423211	0.91067998	0.55833524	0.68864686	1.08911692	0.71761737	1.45073947	0.5637861	1.569088	1.37570055	2.10100514	1.0336061	0.88217886	0	0.62522823	0.55873137	0.28543115	0.82043041	1.84561519	1.57532434
TRD	1.28712006	1.11575062	1.71774548	1.43927471	2.07565971	1.80852516	0.41643585	0.41615298	1.20278096	1.82368875	1.73200492	1.47774443	2.54520668	1.72062821	1.80164007	0.49651656	0.45864812	0.88817851	1.12189743	1.5968104	1.17919244	0.91102165	0.85166311	2.16618355	1.97299286	2.50358518	1.5726991	1.3484734	0.62522823	0	0.3613216	0.51567887	1.19875301	2.41863952	2.19824096
VAL	1.08523139	0.92994496	1.4537529	1.22776543	1.76919985	1.61134497	0.63834434	0.51254321	0.88224863	1.80416131	1.42484422	1.22593134	2.37319289	1.40893657	1.9291805	0.40732701	0.64839308	0.5786248	0.83723243	1.32049629	1.02364692	1.12090859	0.74352013	1.9425626	1.80900377	2.1635647	1.32677297	1.05424465	0.55873137	0.3613216	0	0.39243698	0.87762484	2.16534374	2.06456033
VOD	1.15203636	1.05824293	1.24750578	1.18077389	1.63281686	1.69957804	0.8489869	0.70301129	0.83647119	1.42312228	1.30603704	0.97812779	2.04985496	1.30892125	1.5723731	0.37822399	0.70334248	0.48883099	0.65458196	1.18592959	0.67392738	1.40874541	0.40054904	1.70337906	1.58795205	2.1045639	1.0718644	0.89893992	0.28543115	0.51567887	0.39243698	0	0.81434665	1.9359048	1.74310521
VRE	0.82274898	0.85973398	0.66301519	0.74384411	0.89649476	1.2558935	1.39232938	1.16697907	0.06381512	1.61070961	0.55095944	0.56998106	1.74459752	0.5329319	2.10516902	0.82941426	1.44028721	0.33549411	0.30123589	0.50898635	0.83657562	1.91079661	0.91134171	1.19305727	1.21832338	1.31256607	0.65331172	0.26035357	0.82043041	1.19875301	0.87762484	0.81434665	0	1.35899353	1.52187976
ZAG	1.76265977	1.94001461	0.71335589	1.4274884	0.62849768	1.91278998	2.61413123	2.37871909	1.37398165	1.5566941	0.8698133	0.98458631	0.74710286	0.92461103	2.38212969	1.98816062	2.61112957	1.59928626	1.35073958	0.94188152	1.46370954	3.19313596	1.81558472	0.39006452	0.9554337	1.04874655	0.88646413	1.13095962	1.84561519	2.41863952	2.16534374	1.9359048	1.35899353	0	0.88754882
ZAL	1.77392168	1.92444576	0.97107482	1.43185113	1.28234689	2.02583368	2.37301839	2.16451407	1.53835731	0.81293235	1.29092402	1.07408482	0.54883701	1.34582904	1.67919527	1.78157327	2.39008444	1.61695241	1.40501401	1.17173779	1.27774743	2.98713848	1.6147924	0.63850247	0.70417581	1.85327544	0.98829933	1.29802321	1.57532434	2.19824096	2.06456033	1.74310521	1.52187976	0.88754882	0

g) Pedological niche

Locality	BAL	BAZ	CAV	DOS	EBE	FAL	FEN	GAV	GNI	GOS	HIR	HOD	HUS	JAK	KAM	KOL	LAG	LOI	LOR	LYS	MAR	MIS	OMS	PIT	POD	PON	RAD	STR	TAN	TRD	VAL	VOD	VRE	ZAG	ZAL
BAL	0	0.76201392	1.85986878	1.00223652	1.52733368	1.50552257	1.30145821	1.07263566	0.6865488	1.61031731	2.00022843	1.34307823	2.12345232	1.54052803	0.69154321	1.32128568	2.00588983	1.40428227	1.72523697	1.525159	0.71109978	2.21127104	1.72646815	0.77006312	0.9695098	0.61463052	1.71877078	1.56057566	0.86388192	1.77373472	1.1559656	0.18165646	1.54477213	0.7936452	2.1181102
BAZ	0.76201392	0	1.31862041	0.29239995	1.00304761	1.28753238	0.68767776	0.48997342	0.44354586	0.92283826	1.46012967	0.85173285	1.39923626	1.07808388	0.85844927	1.26105398	1.92692649	0.92032322	1.43768423	1.04804617	0.56501517	1.92963344	1.45920228	0.57959678	0.75434658	0.27906583	1.34934175	1.26065566	1.12850141	1.44228334	0.62838397	0.58860402	1.59550261	0.39996047	1.46888787
CAV	1.85986878	1.31862041	0	1.23932599	1.18529053	1.55430952	0.66123041	1.53828926	1.72930562	0.5977712	0.98506386	0.60809061	1.37517163	1.3652497	2.14701946	1.62349353	2.76119653	1.18662848	1.94090002	0.40660184	1.71286236	2.69111333	0.85269128	1.14964262	1.80069783	1.29452174	0.47240773	0.62105904	2.21913941	2.03691658	1.00704312	1.69371208	2.87322909	1.65308097	1.40056793
DOS	1.00223652	0.29239995	1.23932599	0	0.75600911	1.1377873	0.66625659	0.57214863	0.53469647	0.75407284	1.23430373	0.77072686	1.29156828	0.83796847	0.97095204	1.18186719	1.78778744	0.69642715	1.23112295	1.05247369	0.58358625	1.73445314	1.40237037	0.67280397	0.67215624	0.46987961	1.31388852	1.31052675	1.17179746	1.22303768	0.46270417	0.83761009	1.63729632	0.43084903	1.4433075
EBE	1.52733368	1.00304761	1.18529053	0.75600911	0	0.62613114	0.99752085	1.30547936	1.13154766	0.7800389	0.59937208	0.74321703	1.61656164	0.18714799	1.47706299	0.85294065	1.59797777	0.13777982	0.76596437	1.22105108	0.97956822	1.54574311	1.11592398	0.95437123	0.89457464	1.00814888	1.20096096	1.42914499	1.38486624	0.85507017	0.45091504	1.39112176	2.09740076	1.03886202	1.87016785
FAL	1.50552257	1.28753238	1.55430952	1.1377873	0.62613114	0	1.40602844	1.69916777	1.30743267	1.30987722	0.9304166	1.03546679	2.21504491	0.5207315	1.51826714	0.31963686	1.42056844	0.55470796	0.63663353	1.54746022	1.04741893	1.60228632	1.09363721	1.01938221	0.96185797	1.17003612	1.37290038	1.61479436	1.1896062	0.87601974	0.78302225	1.42667857	2.20340899	1.26894103	2.42735653
FEN	1.30145821	0.68767776	0.66123041	0.66625659	0.99752085	1.40602844	0	0.89327911	1.12058253	0.43474282	1.16068712	0.46452184	1.12432111	1.16036945	1.54273096	1.43463005	2.39151553	0.95940789	1.69599776	0.41487927	1.16943009	2.33094438	1.1016987	0.7251497	1.29968926	0.72527109	0.82558024	0.75030503	1.72221885	1.74396519	0.64909714	1.12309146	2.25344859	1.04979452	1.14697127
GAV	1.07263566	0.48997342	1.53828926	0.57214863	1.30547936	1.69916777	0.89327911	0	0.63802312	1.08176209	1.74365656	1.21073476	1.13479995	1.38657133	0.98757809	1.71523905	2.11086867	1.26203678	1.71998568	1.2857446	0.88730755	1.99913368	1.87192011	1.05786407	1.01833854	0.7569395	1.68981906	1.56627972	1.42626764	1.65390247	1.01738495	0.92589562	1.47707769	0.57862811	1.19678681
GNI	0.6865488	0.44354586	1.72930562	0.53469647	1.13154766	1.30743267	1.12058253	0.63802312	0	1.28596171	1.68617855	1.2106197	1.6833727	1.13573414	0.43992102	1.26788324	1.60740776	1.04531877	1.31323913	1.48043742	0.29319214	1.65402914	1.75926331	0.84091482	0.47684858	0.5493446	1.72811208	1.66857277	0.78832014	1.27833312	0.86975005	0.58740461	1.18474866	0.13023337	1.79717335
GOS	1.61031731	0.92283826	0.5977712	0.75407284	0.7800389	1.30987722	0.43474282	1.08176209	1.28596171	0	0.83113735	0.51292173	1.02049451	0.96368232	1.72178311	1.43280083	2.29412783	0.80230608	1.53077054	0.64147016	1.29292353	2.16040569	1.10450157	0.97779849	1.33846468	0.99720564	0.88004482	0.99083387	1.84605751	1.56777558	0.63807236	1.43299341	2.35939418	1.18449894	1.18492292
HIR	2.00022843	1.46012967	0.98506386	1.23430373	0.59937208	0.9304166	1.16068712	1.74365656	1.68617855	0.83113735	0	0.8343747	1.72428657	0.70964522	2.05471208	1.16667502	2.07883472	0.68066404	1.19216235	1.20967531	1.54908543	1.99540151	0.93959911	1.29642086	1.48980628	1.44089644	1.02850416	1.38197012	1.9435594	1.31570607	0.86108561	1.8550631	2.68945117	1.59201982	1.96979866
HOD	1.34307823	0.85173285	0.60809061	0.77072686	0.74321703	1.03546679	0.46452184	1.21073476	1.2106197	0.51292173	0.8343747	0	1.48715885	0.89883984	1.6038297	1.06821583	2.22176239	0.68858512	1.44379232	0.51552894	1.14251166	2.22900269	0.68231287	0.59469807	1.23501343	0.77113883	0.55416291	0.68935273	1.61836353	1.55489053	0.44239636	1.18391801	2.37633251	1.14671492	1.57305214
HUS	2.12345232	1.39923626	1.37517163	1.29156828	1.61656164	2.21504491	1.12432111	1.13479995	1.6833727	1.02049451	1.72428657	1.48715885	0	1.76406548	2.08051106	2.34926821	2.79989477	1.66168995	2.22625487	1.36367895	1.84306582	2.49141729	2.09977139	1.77026491	1.88319471	1.62016605	1.78630928	1.74490305	2.42702571	2.1478742	1.50826604	1.95081979	2.42487471	1.57712601	0.43774332
JAK	1.54052803	1.07808388	1.3652497	0.83796847	0.18714799	0.5207315	1.16036945	1.38657133	1.13573414	0.96368232	0.70964522	0.89883984	1.76406548	0	1.43922971	0.77533725	1.42389573	0.21685315	0.5797768	1.39033772	0.9513177	1.3995399	1.21905349	1.02772236	0.82602488	1.07378241	1.35011192	1.57956601	1.29199908	0.68665591	0.56608385	1.41973047	2.03440533	1.051176	2.02827751
KAM	0.69154321	0.85844927	2.14701946	0.97095204	1.47706299	1.51826714	1.54273096	0.98757809	0.43992102	1.72178311	2.05471208	1.6038297	2.08051106	1.43922971	0	1.4303855	1.51472549	1.38384336	1.4615955	1.88105442	0.51233014	1.66060364	2.08528603	1.14803711	0.63263382	0.89351715	2.10009726	2.0262812	0.58489817	1.41543125	1.25419632	0.70691359	0.8931698	0.55159624	2.18208108
KOL	1.32128568	1.26105398	1.62349353	1.18186719	0.85294065	0.31963686	1.43463005	1.71523905	1.26788324	1.43280083	1.16667502	1.06821583	2.34926821	0.77533725	1.4303855	0	1.51188007	0.74544929	0.86318603	1.55161676	0.9954073	1.78050821	1.07247326	0.9022493	0.97260423	1.08612596	1.37143883	1.55899223	1.0548305	1.09196565	0.84877366	1.26847653	2.18328704	1.25940402	2.51491536
LAG	2.00588983	1.92692649	2.76119653	1.78778744	1.59797777	1.42056844	2.39151553	2.11086867	1.60740776	2.29412783	2.07883472	2.22176239	2.79989477	1.42389573	1.51472549	1.51188007	0	1.57957499	0.89296747	2.69834997	1.41881755	0.61817354	2.49483871	2.02294914	1.17776008	1.94241164	2.69899792	2.8577994	1.19041464	0.79028618	1.79718862	1.99861917	1.58387565	1.59739143	3.09296946
LOI	1.40428227	0.92032322	1.18662848	0.69642715	0.13777982	0.55470796	0.95940789	1.26203678	1.04531877	0.80230608	0.68066404	0.68858512	1.66168995	0.21685315	1.38384336	0.74544929	1.57957499	0	0.76648215	1.18311084	0.87785576	1.57329509	1.0633569	0.8334382	0.81349544	0.89895907	1.16189971	1.36968297	1.27780876	0.87344373	0.35713562	1.27204139	2.04448753	0.96244703	1.89080085
LOR	1.72523697	1.43768423	1.94090002	1.23112295	0.76596437	0.63663353	1.69599776	1.71998568	1.31323913	1.53077054	1.19216235	1.44379232	2.22625487	0.5797768	1.4615955	0.86318603	0.89296747	0.76648215	0	1.94556347	1.08064176	0.97717991	1.67386849	1.41553573	0.85745725	1.42634097	1.88135265	2.10489725	1.16648353	0.26789233	1.06787884	1.65330654	1.90774986	1.25798931	2.51747427
LYS	1.525159	1.04804617	0.40660184	1.05247369	1.22105108	1.54746022	0.41487927	1.2857446	1.48043742	0.64147016	1.20967531	0.51552894	1.36367895	1.39033772	1.88105442	1.55161676	2.69834997	1.18311084	1.94556347	0	1.49548458	2.6719246	0.90882587	0.88122035	1.63532563	1.00330038	0.52195247	0.38853264	2.00175408	2.03448245	0.90218679	1.36166085	2.63973616	1.42414416	1.31655248
MAR	0.71109978	0.56501517	1.71286236	0.58358625	0.97956822	1.04741893	1.16943009	0.88730755	0.29319214	1.29292353	1.54908543	1.14251166	1.84306582	0.9513177	0.51233014	0.9954073	1.41881755	0.87785576	1.08064176	1.49548458	0	1.53684289	1.61508479	0.75539979	0.26825134	0.55854008	1.65504918	1.64972095	0.59120103	1.08557289	0.76102045	0.63187727	1.29994126	0.32342304	1.98364406
MIS	2.21127104	1.92963344	2.69111333	1.73445314	1.54574311	1.60228632	2.33094438	1.99913368	1.65402914	2.16040569	1.99540151	2.22900269	2.49141729	1.3995399	1.66060364	1.78050821	0.61817354	1.57329509	0.97717991	2.6719246	1.53684289	0	2.60081889	2.1477163	1.27321012	2.02379862	2.7341809	2.9037819	1.52648253	0.74124645	1.80685358	2.16226684	1.59273887	1.60353297	2.83634622
OMS	1.72646815	1.45920228	0.85269128	1.40237037	1.11592398	1.09363721	1.1016987	1.87192011	1.75926331	1.10450157	0.93959911	0.68231287	2.09977139	1.21905349	2.08528603	1.07247326	2.49483871	1.0633569	1.67386849	0.90882587	1.61508479	2.60081889	0	0.98916833	1.69054429	1.29051538	0.44346348	0.77897175	1.9378912	1.86652469	0.98100981	1.61017577	2.91091553	1.71890497	2.16432363
PIT	0.77006312	0.57959678	1.14964262	0.67280397	0.95437123	1.01938221	0.7251497	1.05786407	0.84091482	0.97779849	1.29642086	0.59469807	1.77026491	1.02772236	1.14803711	0.9022493	2.02294914	0.8334382	1.41553573	0.88122035	0.75539979	2.1477163	0.98916833	0	0.94217762	0.33088731	0.98036723	0.92929115	1.15576919	1.52420582	0.53016606	0.63179821	2.00635931	0.83854792	1.81120346
POD	0.9695098	0.75434658	1.80069783	0.67215624	0.89457464	0.96185797	1.29968926	1.01833854	0.47684858	1.33846468	1.48980628	1.23501343	1.88319471	0.82602488	0.63263382	0.97260423	1.17776008	0.81349544	0.85745725	1.63532563	0.26825134	1.27321012	1.69054429	0.94217762	0	0.78274457	1.76238359	1.81132699	0.60180917	0.83236353	0.80788421	0.89944328	1.26824342	0.45821163	2.07765312
PON	0.61463052	0.27906583	1.29452174	0.46987961	1.00814888	1.17003612	0.72527109	0.7569395	0.5493446	0.99720564	1.44089644	0.77113883	1.62016605	1.07378241	0.89351715	1.08612596	1.94241164	0.89895907	1.42634097	1.00330038	0.55854008	2.02379862	1.29051538	0.33088731	0.78274457	0	1.22779525	1.13556733	1.047726	1.4790606	0.5884529	0.44147021	1.72334311	0.55120887	1.66556155
RAD	1.71877078	1.34934175	0.47240773	1.31388852	1.20096096	1.37290038	0.82558024	1.68981906	1.72811208	0.88004482	1.02850416	0.55416291	1.78630928	1.35011192	2.10009726	1.37143883	2.69899792	1.16189971	1.88135265	0.52195247	1.65504918	2.7341809	0.44346348	0.98036723	1.76238359	1.22779525	0	0.42074616	2.07593083	2.02942661	0.98206653	1.57757911	2.90810663	1.67772644	1.79635101
STR	1.56057566	1.26065566	0.62105904	1.31052675	1.42914499	1.61479436	0.75030503	1.56627972	1.66857277	0.99083387	1.38197012	0.68935273	1.74490305	1.57956601	2.0262812	1.55899223	2.8577994	1.36968297	2.10489725	0.38853264	1.64972095	2.9037819	0.77897175	0.92929115	1.81132699	1.13556733	0.42074616	0	2.08382766	2.23158013	1.09719445	1.42335259	2.84549565	1.63595049	1.6609227
TAN	0.86388192	1.12850141	2.21913941	1.17179746	1.38486624	1.1896062	1.72221885	1.42626764	0.78832014	1.84605751	1.9435594	1.61836353	2.42702571	1.29199908	0.58489817	1.0548305	1.19041464	1.27780876	1.16648353	2.00175408	0.59120103	1.52648253	1.9378912	1.15576919	0.60180917	1.047726	2.07593083	2.08382766	0	1.19636297	1.24980711	0.9111997	1.2428781	0.86715091	2.56696461
TRD	1.77373472	1.44228334	2.03691658	1.22303768	0.85507017	0.87601974	1.74396519	1.65390247	1.27833312	1.56777558	1.31570607	1.55489053	2.1478742	0.68665591	1.41543125	1.09196565	0.79028618	0.87344373	0.26789233	2.03448245	1.08557289	0.74124645	1.86652469	1.52420582	0.83236353	1.4790606	2.02942661	2.23158013	1.19636297	0	1.15269863	1.69871487	1.7512505	1.2142867	2.46113641
VAL	1.1559656	0.62838397	1.00704312	0.46270417	0.45091504	0.78302225	0.64909714	1.01738495	0.86975005	0.63807236	0.86108561	0.44239636	1.50826604	0.56608385	1.25419632	0.84877366	1.79718862	0.35713562	1.06787884	0.90218679	0.76102045	1.80685358	0.98100981	0.53016606	0.80788421	0.5884529	0.98206653	1.09719445	1.24980711	1.15269863	0	1.0033539	1.990999	0.79496763	1.66836462
VOD	0.18165646	0.58860402	1.69371208	0.83761009	1.39112176	1.42667857	1.12309146	0.92589562	0.58740461	1.43299341	1.8550631	1.18391801	1.95081979	1.41973047	0.70691359	1.26847653	1.99861917	1.27204139	1.65330654	1.36166085	0.63187727	2.16226684	1.61017577	0.63179821	0.89944328	0.44147021	1.57757911	1.42335259	0.9111997	1.69871487	1.0033539	0	1.56928508	0.67690774	1.95075405
VRE	1.54477213	1.59550261	2.87322909	1.63729632	2.09740076	2.20340899	2.25344859	1.47707769	1.18474866	2.35939418	2.68945117	2.37633251	2.42487471	2.03440533	0.8931698	2.18328704	1.58387565	2.04448753	1.90774986	2.63973616	1.29994126	1.59273887	2.91091553	2.00635931	1.26824342	1.72334311	2.90810663	2.84549565	1.2428781	1.7512505	1.990999	1.56928508	0	1.23335629	2.57467241
ZAG	0.7936452	0.39996047	1.65308097	0.43084903	1.03886202	1.26894103	1.04979452	0.57862811	0.13023337	1.18449894	1.59201982	1.14671492	1.57712601	1.051176	0.55159624	1.25940402	1.59739143	0.96244703	1.25798931	1.42414416	0.32342304	1.60353297	1.71890497	0.83854792	0.45821163	0.55120887	1.67772644	1.63595049	0.86715091	1.2142867	0.79496763	0.67690774	1.23335629	0	1.71198001
ZAL	2.1181102	1.46888787	1.40056793	1.4433075	1.87016785	2.42735653	1.14697127	1.19678681	1.79717335	1.18492292	1.96979866	1.57305214	0.43774332	2.02827751	2.18208108	2.51491536	3.09296946	1.89080085	2.51747427	1.31655248	1.98364406	2.83634622	2.16432363	1.81120346	2.07765312	1.66556155	1.79635101	1.6609227	2.56696461	2.46113641	1.66836462	1.95075405	2.57467241	1.71198001	0

h) Biotic niche

Locality	BAL	BAZ	CAV	DOS	EBE	FAL	FEN	GAV	GNI	GOS	HIR	HOD	HUS	JAK	KAM	KOL	LAG
BAL	0	0.63685501	1.17090351	0.99696891	1.70267011	1.05543922	2.0474997	0.97988031	1.40778204	1.27366117	1.76910793	1.59934471	0.8501432	1.2786553	2.3300346	1.18262171	1.88425741
BAZ	0.63685501	0	1.07103527	0.41879657	1.08402054	0.60792273	1.63728432	0.43766037	0.83120473	0.86402858	1.30590513	1.15491604	1.38391606	0.92895634	2.02348224	0.86611683	1.35138595
CAV	1.17090351	1.07103527	0	1.13365664	1.43637111	1.0531408	1.85340935	0.97323345	1.05990124	0.59837247	1.03907335	0.83463977	1.21462234	0.87876693	1.86369672	0.91438441	1.21193007
DOS	0.99696891	0.41879657	1.13365664	0	0.74171048	0.29710108	1.71903245	0.53102129	0.66397236	0.80419899	1.01472123	1.05237306	1.69498641	0.67238648	2.12839261	1.03871202	1.06261561
EBE	1.70267011	1.08402054	1.43637111	0.74171048	0	0.82933297	1.55286082	0.86785269	0.50927145	0.89264937	0.75747257	0.88789947	2.32685419	0.93880817	1.97290503	1.22697826	0.58670873
FAL	1.05543922	0.60792273	1.0531408	0.29710108	0.82933297	0	1.94873859	0.74360995	0.80307652	0.82508014	0.89539454	1.08933635	1.6627798	0.41560468	2.29868549	1.1985439	1.03494847
FEN	2.0474997	1.63728432	1.85340935	1.71903245	1.55286082	1.94873859	0	1.25507795	1.22302314	1.41841203	1.88924568	1.29047947	2.49349953	2.09025807	0.70544513	0.99498077	1.64611415
GAV	0.97988031	0.43766037	0.97323345	0.53102129	0.86785269	0.74360995	1.25507795	0	0.4693064	0.57220013	1.10844319	0.7897256	1.58943199	0.96062322	1.62680209	0.5224409	1.06918544
GNI	1.40778204	0.83120473	1.05990124	0.66397236	0.50927145	0.80307652	1.22302314	0.4693064	0	0.47463153	0.77515564	0.50904478	1.95685006	0.89929641	1.57316341	0.72404139	0.63706149
GOS	1.27366117	0.86402858	0.59837247	0.80419899	0.89264937	0.82508014	1.41841203	0.57220013	0.47463153	0	0.70728685	0.34168497	1.63568237	0.76076129	1.57804589	0.62707382	0.73553494
HIR	1.76910793	1.30590513	1.03907335	1.01472123	0.75747257	0.89539454	1.88924568	1.10844319	0.77515564	0.70728685	0	0.68814828	2.14497391	0.63863486	2.06416276	1.31035431	0.34383636
HOD	1.59934471	1.15491604	0.83463977	1.05237306	0.88789947	1.08933635	1.29047947	0.7897256	0.50904478	0.34168497	0.68814828	0	1.93654047	0.99335514	1.39587329	0.73195392	0.59330418
HUS	0.8501432	1.38391606	1.21462234	1.69498641	2.32685419	1.6627798	2.49349953	1.58943199	1.95685006	1.63568237	2.14497391	1.93654047	0	1.69601128	2.55147081	1.56007662	2.32088676
JAK	1.2786553	0.92895634	0.87876693	0.67238648	0.93880817	0.41560468	2.09025807	0.96062322	0.89929641	0.76076129	0.63863486	0.99335514	1.69601128	0	2.33251536	1.29622544	0.89566831
KAM	2.3300346	2.02348224	1.86369672	2.12839261	1.97290503	2.29868549	0.70544513	1.62680209	1.57316341	1.57804589	2.06416276	1.39587329	2.55147081	2.33251536	0	1.18981377	1.85812557
KOL	1.18262171	0.86611683	0.91438441	1.03871202	1.22697826	1.1985439	0.99498077	0.5224409	0.72404139	0.62707382	1.31035431	0.73195392	1.56007662	1.29622544	1.18981377	0	1.24122879
LAG	1.88425741	1.35138595	1.21193007	1.06261561	0.58670873	1.03494847	1.64611415	1.06918544	0.63706149	0.73553494	0.34383636	0.59330418	2.32088676	0.89566831	1.85812557	1.24122879	0
LOI	1.06675144	0.60929298	1.08637245	0.77300169	1.02247696	0.99944848	1.03945463	0.25674022	0.5887437	0.69946123	1.29366595	0.85152929	1.64083916	1.20640975	1.42706718	0.37652287	1.21054761
LOR	1.37608473	0.8096071	1.1147116	0.72856116	0.64470624	0.91572718	1.05480531	0.39803424	0.19383075	0.54995226	0.96159637	0.58013939	1.93755827	1.05399929	1.4421821	0.59913532	0.80890732
LYS	0.54917926	0.78577672	0.67068544	1.05559637	1.64209526	1.03952896	1.96109039	0.93287595	1.27143667	0.96608805	1.49618826	1.28549226	0.69016317	1.08672544	2.0966038	0.97975242	1.646349
MAR	1.61335708	1.25891764	0.75254163	1.23807782	1.16474542	1.27388498	1.27903847	0.90101468	0.73979223	0.4516586	0.91133263	0.28403741	1.84014308	1.16205174	1.25565528	0.67450921	0.85745682
MIS	1.43226187	1.08348801	0.9570524	1.18225697	1.21859376	1.32139568	0.89927216	0.69173248	0.71961791	0.61755205	1.24835171	0.59181826	1.75583955	1.36112937	1.00587233	0.26724395	1.14092406
OMS	1.3880347	1.19500459	1.0256761	1.39814646	1.55835315	1.53855439	0.99060351	0.88776623	1.0518404	0.88399205	1.56258287	0.92171206	1.59352445	1.59091052	0.97846063	0.36758059	1.49237406
PIT	2.11932772	1.97748551	1.16823452	2.05989823	2.05415368	2.07258419	1.6929032	1.67313384	1.62096308	1.2783695	1.68377993	1.16758281	1.99964156	1.90560371	1.26166571	1.25550597	1.67593811
POD	2.03332811	1.83540482	1.53362768	1.99788406	1.99040371	2.13794312	0.96323976	1.48393906	1.52060106	1.39182477	1.96709958	1.28310892	2.13145885	2.14553985	0.49377428	0.97486949	1.83677048
PON	1.82692729	1.28027689	1.45108629	1.19437122	0.84403127	1.37439154	0.75354615	0.8584009	0.57858483	0.8866464	1.17964203	0.71177936	2.33995681	1.46397359	1.15134993	0.82781422	0.90974711
RAD	2.03731955	1.62847962	1.32402144	1.60806289	1.34663718	1.71311694	0.91811882	1.20273971	0.97975014	0.94860142	1.29612015	0.67185431	2.31047158	1.66336142	0.79982179	0.89115513	1.09498804
STR	1.07524307	1.22614144	0.51351142	1.4363464	1.85757449	1.39988393	1.97578441	1.20145644	1.42899122	1.00712609	1.54754662	1.23931102	0.82903868	1.31167955	1.92223154	1.02497048	1.69591123
TAN	2.31193131	2.0537179	1.93364502	2.20085921	2.11023735	2.38263519	0.75389062	1.68268439	1.69042053	1.68477451	2.22100634	1.54089261	2.51113393	2.4388928	0.23450552	1.22660695	2.02926504
TRD	0.34801118	0.36076812	0.96920313	0.66988827	1.37098076	0.71884435	1.89445567	0.71219115	1.09809687	0.98094491	1.43210889	1.3115816	1.05398185	0.95188815	2.19700933	1.01046851	1.54766982
VAL	3.01675633	2.73904848	2.66652467	2.86698236	2.68514168	3.06605212	1.2085337	2.36304017	2.32933579	2.3853615	2.85502815	2.20155324	3.22638889	3.13319638	0.81807641	1.9460026	2.62010849
VOD	2.50464398	2.14941794	1.91547479	2.19623303	1.94089363	2.34524263	0.84878723	1.72913429	1.58663421	1.59986251	1.98832798	1.35913494	2.73100823	2.33953009	0.32639416	1.32934558	1.76221867
VRE	0.72625946	1.14641403	1.051297	1.49986269	2.07774141	1.53741263	2.04415912	1.26688328	1.65504688	1.36519517	1.97537652	1.64084848	0.47141348	1.61321401	2.10871223	1.15292278	2.08971735
ZAG	1.88467485	1.63578563	1.32305762	1.76498105	1.73489143	1.89566175	0.88973167	1.26447005	1.2658764	1.13424964	1.69795567	1.01318202	2.03548858	1.89228726	0.56307234	0.77039404	1.56793101
ZAL	1.23024207	0.75018013	1.12882205	0.37551577	0.71035654	0.17637965	1.96261202	0.81728745	0.77527253	0.84499203	0.786774	1.06621231	1.82573148	0.39178332	2.31815443	1.26925085	0.92019617

Locality	LOI	LOR	LYS	MAR	MIS	OMS	PIT	POD	PON	RAD	STR	TAN	TRD	VAL	VOD	VRE	ZAG	ZAL
BAL	1.06675144	1.37608473	0.54917926	1.61335708	1.43226187	1.3880347	2.11932772	2.03332811	1.82692729	2.03731955	1.07524307	2.31193131	0.34801118	3.01675633	2.50464398	0.72625946	1.88467485	1.23024207
BAZ	0.60929298	0.8096071	0.78577672	1.25891764	1.08348801	1.19500459	1.97748551	1.83540482	1.28027689	1.62847962	1.22614144	2.0537179	0.36076812	2.73904848	2.14941794	1.14641403	1.63578563	0.75018013
CAV	1.08637245	1.1147116	0.67068544	0.75254163	0.9570524	1.0256761	1.16823452	1.53362768	1.45108629	1.32402144	0.51351142	1.93364502	0.96920313	2.66652467	1.91547479	1.051297	1.32305762	1.12882205
DOS	0.77300169	0.72856116	1.05559637	1.23807782	1.18225697	1.39814646	2.05989823	1.99788406	1.19437122	1.60806289	1.4363464	2.20085921	0.66988827	2.86698236	2.19623303	1.49986269	1.76498105	0.37551577
EBE	1.02247696	0.64470624	1.64209526	1.16474542	1.21859376	1.55835315	2.05415368	1.99040371	0.84403127	1.34663718	1.85757449	2.11023735	1.37098076	2.68514168	1.94089363	2.07774141	1.73489143	0.71035654
FAL	0.99944848	0.91572718	1.03952896	1.27388498	1.32139568	1.53855439	2.07258419	2.13794312	1.37439154	1.71311694	1.39988393	2.38263519	0.71884435	3.06605212	2.34524263	1.53741263	1.89566175	0.17637965
FEN	1.03945463	1.05480531	1.96109039	1.27903847	0.89927216	0.99060351	1.6929032	0.96323976	0.75354615	0.91811882	1.97578441	0.75389062	1.89445567	1.2085337	0.84878723	2.04415912	0.88973167	1.96261202
GAV	0.25674022	0.39803424	0.93287595	0.90101468	0.69173248	0.88776623	1.67313384	1.48393906	0.8584009	1.20273971	1.20145644	1.68268439	0.71219115	2.36304017	1.72913429	1.26688328	1.26447005	0.81728745
GNI	0.5887437	0.19383075	1.27143667	0.73979223	0.71961791	1.0518404	1.62096308	1.52060106	0.57858483	0.97975014	1.42899122	1.69042053	1.09809687	2.32933579	1.58663421	1.65504688	1.2658764	0.77527253
GOS	0.69946123	0.54995226	0.96608805	0.4516586	0.61755205	0.88399205	1.2783695	1.39182477	0.8866464	0.94860142	1.00712609	1.68477451	0.98094491	2.3853615	1.59986251	1.36519517	1.13424964	0.84499203
HIR	1.29366595	0.96159637	1.49618826	0.91133263	1.24835171	1.56258287	1.68377993	1.96709958	1.17964203	1.29612015	1.54754662	2.22100634	1.43210889	2.85502815	1.98832798	1.97537652	1.69795567	0.786774
HOD	0.85152929	0.58013939	1.28549226	0.28403741	0.59181826	0.92171206	1.16758281	1.28310892	0.71177936	0.67185431	1.23931102	1.54089261	1.3115816	2.20155324	1.35913494	1.64084848	1.01318202	1.06621231
HUS	1.64083916	1.93755827	0.69016317	1.84014308	1.75583955	1.59352445	1.99964156	2.13145885	2.33995681	2.31047158	0.82903868	2.51113393	1.05398185	3.22638889	2.73100823	0.47141348	2.03548858	1.82573148
JAK	1.20640975	1.05399929	1.08672544	1.16205174	1.36112937	1.59091052	1.90560371	2.14553985	1.46397359	1.66336142	1.31167955	2.4388928	0.95188815	3.13319638	2.33953009	1.61321401	1.89228726	0.39178332
KAM	1.42706718	1.4421821	2.0966038	1.25565528	1.00587233	0.97846063	1.26166571	0.49377428	1.15134993	0.79982179	1.92223154	0.23450552	2.19700933	0.81807641	0.32639416	2.10871223	0.56307234	2.31815443
KOL	0.37652287	0.59913532	0.97975242	0.67450921	0.26724395	0.36758059	1.25550597	0.97486949	0.82781422	0.89115513	1.02497048	1.22660695	1.01046851	1.9460026	1.32934558	1.15292278	0.77039404	1.26925085
LAG	1.21054761	0.80890732	1.646349	0.85745682	1.14092406	1.49237406	1.67593811	1.83677048	0.90974711	1.09498804	1.69591123	2.02926504	1.54766982	2.62010849	1.76221867	2.08971735	1.56793101	0.92019617
LOI	0	0.43891099	1.02731083	0.91262831	0.56853871	0.72235475	1.61591255	1.29740654	0.78716352	1.11385845	1.24369495	1.46394878	0.86315647	2.13734513	1.5609608	1.26265593	1.09934205	1.07097464
LOR	0.43891099	0	1.26710951	0.75946551	0.61131764	0.93013536	1.60332908	1.39933022	0.48703003	0.93066358	1.42666176	1.54455879	1.09426489	2.18267539	1.48461943	1.60077832	1.15679778	0.91344934
LYS	1.02731083	1.26710951	0	1.2389673	1.17464903	1.12367874	1.64401151	1.74363739	1.69102069	1.73209252	0.54431379	2.10232855	0.51163551	2.83944157	2.23754608	0.52967517	1.58114132	1.19337487
MAR	0.91262831	0.75946551	1.2389673	0	0.49589001	0.75454981	0.89210525	1.07152242	0.85102844	0.58413468	1.08622816	1.38874626	1.36579069	2.07279862	1.23608722	1.5280039	0.80851547	1.27869218
MIS	0.56853871	0.61131764	1.17464903	0.49589001	0	0.3686124	1.08476892	0.82613814	0.69974968	0.62617597	1.12453654	1.08314087	1.2380613	1.79818019	1.1022906	1.35754449	0.58308675	1.36284235
OMS	0.72235475	0.93013536	1.12367874	0.75454981	0.3686124	0	1.0339452	0.65702408	1.02889262	0.86401888	1.01290453	0.98103221	1.27863925	1.72126775	1.1588609	1.15868737	0.50210049	1.61241556
PIT	1.61591255	1.60332908	1.64401151	0.89210525	1.08476892	1.0339452	0	0.92010458	1.59098328	0.99030275	1.20676096	1.34747073	1.97764083	1.9531315	1.24348698	1.71198052	0.83359768	2.10455401
POD	1.29740654	1.39933022	1.74363739	1.07152242	0.82613814	0.65702408	0.92010458	0	1.26657006	0.83663162	1.50379038	0.47353906	1.93546511	1.17627892	0.70393161	1.70173961	0.27027974	2.18545694
PON	0.78716352	0.48703003	1.69102069	0.85102844	0.69974968	1.02889262	1.59098328	1.26657006	0	0.68301116	1.75267913	1.29580821	1.56731264	1.84131429	1.13364518	1.96381526	1.04169096	1.34073661
RAD	1.11385845	0.93066358	1.73209252	0.58413468	0.62617597	0.86401888	0.99030275	0.83663162	0.68301116	0	1.57552488	0.99274602	1.80897486	1.58121296	0.69650899	1.93430835	0.6051317	1.69675091
STR	1.24369495	1.42666176	0.54431379	1.08622816	1.12453654	1.01290453	1.20676096	1.50379038	1.75267913	1.57552488	0	1.93390887	1.02936304	2.6687001	2.04122658	0.65596836	1.36508489	1.51703476
TAN	1.46394878	1.54455879	2.10232855	1.38874626	1.08314087	0.98103221	1.34747073	0.47353906	1.29580821	0.99274602	1.93390887	0	2.21237475	0.74259777	0.55009689	2.05826748	0.62693676	2.417253
TRD	0.86315647	1.09426489	0.51163551	1.36579069	1.2380613	1.27863925	1.97764083	1.93546511	1.56731264	1.80897486	1.02936304	2.21237475	0	2.92442012	2.33510555	0.89645527	1.75039296	0.8914896
VAL	2.13734513	2.18267539	2.83944157	2.07279862	1.79818019	1.72126775	1.9531315	1.17627892	1.84131429	1.58121296	2.6687001	0.74259777	2.92442012	0	0.91935224	2.76882384	1.34757384	3.08610808
VOD	1.5609608	1.48461943	2.23754608	1.23608722	1.1022906	1.1588609	1.24348698	0.70393161	1.13364518	0.69650899	2.04122658	0.55009689	2.33510555	0.91935224	0	2.30706074	0.70080917	2.34196865
VRE	1.26265593	1.60077832	0.52967517	1.5280039	1.35754449	1.15868737	1.71198052	1.70173961	1.96381526	1.93430835	0.65596836	2.05826748	0.89645527	2.76882384	2.30706074	0	1.61554907	1.69549372
ZAG	1.09934205	1.15679778	1.58114132	0.80851547	0.58308675	0.50210049	0.83359768	0.27027974	1.04169096	0.6051317	1.36508489	0.62693676	1.75039296	1.34757384	0.70080917	1.61554907	0	1.93452475
ZAL	1.07097464	0.91344934	1.19337487	1.27869218	1.36284235	1.61241556	2.10455401	2.18545694	1.34073661	1.69675091	1.51703476	2.417253	0.8914896	3.08610808	2.34196865	1.69549372	1.93452475	0

i) Coenotic niche based on vascular plants

Locality	BAL	BAZ	CAV	DOS	EBE	FAL	FEN	GAV	GNI	GOS	HIR	HOD	HUS	JAK	KAM	KOL	LAG	LOI
BAL	0	0.77164205	1.58983685	1.09981333	2.06128883	2.26414613	1.99895158	0.97501193	1.51251661	1.73704626	1.87003911	1.68117921	1.92615065	1.32344426	2.8635169	1.26378815	1.61817815	1.24610853
BAZ	0.77164205	0	1.65857041	0.9945863	1.98019115	1.91201163	1.43609815	0.51042971	1.0850697	1.80943456	1.65331491	1.63641419	2.15964558	1.68453822	2.48393319	1.11181571	1.1180253	1.10436882
CAV	1.58983685	1.65857041	0	0.7950232	0.8920251	2.62331104	1.9023975	1.23740838	1.45827522	0.48490174	1.28501314	0.24406798	1.35127434	1.15094856	1.78994818	0.60592436	1.3195213	1.23111227
DOS	1.09981333	0.9945863	0.7950232	0	1.01392736	1.91026371	1.2824965	0.55436207	0.76030813	0.82376659	0.89162127	0.77835779	1.31467057	0.98255065	1.7808081	0.42999116	0.75552178	0.52674603
EBE	2.06128883	1.98019115	0.8920251	1.01392736	0	2.18505407	1.54911917	1.49344136	1.23429569	0.51018638	0.68576872	0.81616551	1.02749272	1.23171055	1.15223241	1.09002934	1.18790044	1.08802877
FAL	2.26414613	1.91201163	2.62331104	1.91026371	2.18505407	0	1.13939187	1.83294005	1.25768052	2.38350846	1.50363948	2.55660588	2.17876714	2.15759729	2.32857507	2.2668918	1.60987831	1.4240993
FEN	1.99895158	1.43609815	1.9023975	1.2824965	1.54911917	1.13939187	0	1.15239193	0.55547747	1.78397723	0.96732899	1.75433264	2.04910153	1.98483742	1.46253743	1.47115323	0.63189099	1.04396984
GAV	0.97501193	0.51042971	1.23740838	0.55436207	1.49344136	1.83294005	1.15239193	0	0.73028828	1.36277972	1.21780809	1.17914444	1.82313758	1.43474224	2.00145127	0.66639846	0.68066893	0.76756314
GNI	1.51251661	1.0850697	1.45827522	0.76030813	1.23429569	1.25768052	0.55547747	0.73028828	0	1.34619338	0.68145557	1.3567389	1.61061483	1.45594701	1.56783276	1.03646446	0.40859151	0.50699799
GOS	1.73704626	1.80943456	0.48490174	0.82376659	0.51018638	2.38350846	1.78397723	1.36277972	1.34619338	0	0.973264	0.54730604	0.9284338	0.922721	1.60540472	0.86068523	1.30334653	1.06312468
HIR	1.87003911	1.65331491	1.28501314	0.89162127	0.68576872	1.50363948	0.96732899	1.21780809	0.68145557	0.973264	0	1.19567804	1.14371375	1.25307897	1.21684588	1.15456775	0.81365448	0.65212214
HOD	1.68117921	1.63641419	0.24406798	0.77835779	0.81616551	2.55660588	1.75433264	1.17914444	1.3567389	0.54730604	1.19567804	0	1.45830033	1.30421845	1.58680921	0.5301618	1.16542381	1.21226147
HUS	1.92615065	2.15964558	1.35127434	1.31467057	1.02749272	2.17876714	2.04910153	1.82313758	1.61061483	0.9284338	1.14371375	1.45830033	0	0.63454711	2.09038867	1.59366346	1.79132119	1.18454672
JAK	1.32344426	1.68453822	1.15094856	0.98255065	1.23171055	2.15759729	1.98483742	1.43474224	1.45594701	0.922721	1.25307897	1.30421845	0.63454711	0	2.32113076	1.28053173	1.63423442	0.95713131
KAM	2.8635169	2.48393319	1.78994818	1.7808081	1.15223241	2.32857507	1.46253743	2.00145127	1.56783276	1.60540472	1.21684588	1.58680921	2.09038867	2.32113076	0	1.75274067	1.37922801	1.78798583
KOL	1.26378815	1.11181571	0.60592436	0.42999116	1.09002934	2.2668918	1.47115323	0.66639846	1.03646446	0.86068523	1.15456775	0.5301618	1.59366346	1.28053173	1.75274067	0	0.85124354	0.94037175
LAG	1.61817815	1.1180253	1.3195213	0.75552178	1.18790044	1.60987831	0.63189099	0.68066893	0.40859151	1.30334653	0.81365448	1.16542381	1.79132119	1.63423442	1.37922801	0.85124354	0	0.77803179
LOI	1.24610853	1.10436882	1.23111227	0.52674603	1.08802877	1.4240993	1.04396984	0.76756314	0.50699799	1.06312468	0.65212214	1.21226147	1.18454672	0.95713131	1.78798583	0.94037175	0.77803179	0
LOR	1.52100458	1.72720437	0.91885447	0.84538883	0.83816968	2.11326688	1.79556471	1.37300958	1.29800694	0.57646803	0.96146258	1.03338371	0.50061784	0.39628306	1.94704836	1.10842066	1.42444765	0.85485951
LYS	1.58225078	1.74972531	0.31615125	0.83520808	0.83314758	2.56894175	1.97184792	1.34840135	1.49517786	0.32766326	1.23817439	0.51477788	1.06772977	0.89316687	1.88252715	0.80379322	1.43847313	1.17727395
MAR	1.2602867	1.22592654	0.67646076	0.23573894	0.80852207	1.96115056	1.36087516	0.78648597	0.84905263	0.60307703	0.78556329	0.67743146	1.11575153	0.85668498	1.68775025	0.51467591	0.85732438	0.55499107
MIS	0.64702193	0.27051577	1.78599684	1.09845314	2.08564481	1.85497222	1.54923514	0.69943092	1.17814514	1.89945986	1.72840901	1.79122682	2.13767772	1.63646885	2.65374314	1.28393321	1.29123687	1.12611546
OMS	1.863007	1.86867554	0.39153954	0.91029702	0.54331241	2.53097947	1.81363212	1.39940296	1.42099747	0.28421634	1.08070213	0.36369354	1.20020814	1.19257621	1.48819023	0.81534659	1.29481873	1.22347075
PIT	2.72227111	2.59911647	1.67594464	1.70603343	0.79244607	2.19832064	1.77341453	2.13137291	1.64282123	1.25336545	0.97596725	1.59759631	1.27600214	1.74027167	1.07294241	1.84811829	1.67664152	1.57672212
POD	2.53013789	2.3165108	1.60829832	1.49573282	0.73691829	1.89629899	1.38704104	1.85315517	1.30898145	1.23077565	0.68783423	1.49460056	1.35823059	1.7115741	0.85344539	1.65089558	1.33782275	1.33434983
PON	1.88559358	1.68185996	0.79271017	0.78912759	0.43581801	2.10696471	1.28925964	1.17438973	0.98975404	0.63974798	0.66880812	0.62436962	1.33845722	1.36460229	1.06335103	0.7776064	0.82891787	0.97029664
RAD	1.63268842	1.72156172	0.17208626	0.8002993	0.76826114	2.56188047	1.88779548	1.29214718	1.43798181	0.3212626	1.19492438	0.33105314	1.19109601	1.04260337	1.7464332	0.69804748	1.33477945	1.17919654
STR	2.07320402	2.1965141	0.55911589	1.26521001	0.86104236	2.91669128	2.24688716	1.76092911	1.84031992	0.53931692	1.4685346	0.65462377	1.28357018	1.30749373	1.79847212	1.14426969	1.72499756	1.58903748
TAN	1.97575049	1.68689231	0.90733815	0.89677423	0.66011003	2.16939403	1.23967353	1.17790341	1.02018684	0.86025518	0.8169547	0.69267107	1.59210655	1.59300246	0.95763967	0.79990723	0.76499207	1.10894053
TRD	1.2626423	1.45116043	0.90996515	0.62587512	0.94630005	1.97056714	1.64003518	1.12111812	1.11039893	0.68058816	0.92496297	1.0103251	0.73360266	0.39034971	1.9826475	0.94850243	1.25401919	0.64025443
VAL	2.67980128	2.33683695	2.18046657	1.78989807	1.41389897	1.31723863	1.08479689	1.96859504	1.25694638	1.84329619	0.93677245	2.05633398	1.80803332	2.08062879	1.20847551	2.02817246	1.40356016	1.44773443
VOD	1.88320746	1.52263828	0.97025884	0.85909278	0.89436566	2.15356034	1.15501842	1.02487488	0.95485518	1.01995119	0.9341358	0.7467758	1.76238601	1.68501072	1.08352876	0.70867962	0.62409804	1.112857
VRE	1.38082044	1.79064258	1.76927342	1.3874855	1.74767121	1.94918435	2.12236332	1.68648983	1.63428119	1.53767603	1.54996984	1.90094186	0.98161578	0.65176988	2.73588223	1.76362719	1.92766112	1.1536618
ZAG	2.72664498	2.351652	1.54598336	1.6628745	1.15296436	2.58327296	1.58636711	1.86266292	1.61108623	1.49190042	1.35760803	1.31911282	2.1743627	2.30748007	0.50064447	1.5172572	1.32164982	1.81513205
ZAL	1.83771626	2.01463915	0.83016887	1.05613759	0.66959724	2.38962199	1.97148256	1.60995921	1.52079606	0.40162199	1.06599336	0.94121499	0.56839468	0.72985349	1.82011335	1.20040467	1.57144345	1.15168336

Locality	LOR	LYS	MAR	MIS	OMS	PIT	POD	PON	RAD	STR	TAN	TRD	VAL	VOD	VRE	ZAG	ZAL
BAL	1.52100458	1.58225078	1.2602867	0.64702193	1.863007	2.72227111	2.53013789	1.88559358	1.63268842	2.07320402	1.97575049	1.2626423	2.67980128	1.88320746	1.38082044	2.72664498	1.83771626
BAZ	1.72720437	1.74972531	1.22592654	0.27051577	1.86867554	2.59911647	2.3165108	1.68185996	1.72156172	2.1965141	1.68689231	1.45116043	2.33683695	1.52263828	1.79064258	2.351652	2.01463915
CAV	0.91885447	0.31615125	0.67646076	1.78599684	0.39153954	1.67594464	1.60829832	0.79271017	0.17208626	0.55911589	0.90733815	0.90996515	2.18046657	0.97025884	1.76927342	1.54598336	0.83016887
DOS	0.84538883	0.83520808	0.23573894	1.09845314	0.91029702	1.70603343	1.49573282	0.78912759	0.8002993	1.26521001	0.89677423	0.62587512	1.78989807	0.85909278	1.3874855	1.6628745	1.05613759
EBE	0.83816968	0.83314758	0.80852207	2.08564481	0.54331241	0.79244607	0.73691829	0.43581801	0.76826114	0.86104236	0.66011003	0.94630005	1.41389897	0.89436566	1.74767121	1.15296436	0.66959724
FAL	2.11326688	2.56894175	1.96115056	1.85497222	2.53097947	2.19832064	1.89629899	2.10696471	2.56188047	2.91669128	2.16939403	1.97056714	1.31723863	2.15356034	1.94918435	2.58327296	2.38962199
FEN	1.79556471	1.97184792	1.36087516	1.54923514	1.81363212	1.77341453	1.38704104	1.28925964	1.88779548	2.24688716	1.23967353	1.64003518	1.08479689	1.15501842	2.12236332	1.58636711	1.97148256
GAV	1.37300958	1.34840135	0.78648597	0.69943092	1.39940296	2.13137291	1.85315517	1.17438973	1.29214718	1.76092911	1.17790341	1.12111812	1.96859504	1.02487488	1.68648983	1.86266292	1.60995921
GNI	1.29800694	1.49517786	0.84905263	1.17814514	1.42099747	1.64282123	1.30898145	0.98975404	1.43798181	1.84031992	1.02018684	1.11039893	1.25694638	0.95485518	1.63428119	1.61108623	1.52079606
GOS	0.57646803	0.32766326	0.60307703	1.89945986	0.28421634	1.25336545	1.23077565	0.63974798	0.3212626	0.53931692	0.86025518	0.68058816	1.84329619	1.01995119	1.53767603	1.49190042	0.40162199
HIR	0.96146258	1.23817439	0.78556329	1.72840901	1.08070213	0.97596725	0.68783423	0.66880812	1.19492438	1.4685346	0.8169547	0.92496297	0.93677245	0.9341358	1.54996984	1.35760803	1.06599336
HOD	1.03338371	0.51477788	0.67743146	1.79122682	0.36369354	1.59759631	1.49460056	0.62436962	0.33105314	0.65462377	0.69267107	1.0103251	2.05633398	0.7467758	1.90094186	1.31911282	0.94121499
HUS	0.50061784	1.06772977	1.11575153	2.13767772	1.20020814	1.27600214	1.35823059	1.33845722	1.19109601	1.28357018	1.59210655	0.73360266	1.80803332	1.76238601	0.98161578	2.1743627	0.56839468
JAK	0.39628306	0.89316687	0.85668498	1.63646885	1.19257621	1.74027167	1.7115741	1.36460229	1.04260337	1.30749373	1.59300246	0.39034971	2.08062879	1.68501072	0.65176988	2.30748007	0.72985349
KAM	1.94704836	1.88252715	1.68775025	2.65374314	1.48819023	1.07294241	0.85344539	1.06335103	1.7464332	1.79847212	0.95763967	1.9826475	1.20847551	1.08352876	2.73588223	0.50064447	1.82011335
KOL	1.10842066	0.80379322	0.51467591	1.28393321	0.81534659	1.84811829	1.65089558	0.7776064	0.69804748	1.14426969	0.79990723	0.94850243	2.02817246	0.70867962	1.76362719	1.5172572	1.20040467
LAG	1.42444765	1.43847313	0.85732438	1.29123687	1.29481873	1.67664152	1.33782275	0.82891787	1.33477945	1.72499756	0.76499207	1.25401919	1.40356016	0.62409804	1.92766112	1.32164982	1.57144345
LOI	0.85485951	1.17727395	0.55499107	1.12611546	1.22347075	1.57672212	1.33434983	0.97029664	1.17919654	1.58903748	1.10894053	0.64025443	1.44773443	1.112857	1.1536618	1.81513205	1.15168336
LOR	0	0.66127817	0.64813253	1.73490411	0.85794801	1.37987904	1.35637973	1.01444398	0.77971629	1.0236477	1.25699621	0.28162653	1.81505397	1.38573205	0.97358015	1.93828323	0.39258305
LYS	0.66127817	0	0.66110121	1.834922	0.425087	1.57717022	1.55564256	0.87558733	0.18871558	0.50387017	1.056937	0.71544228	2.14151739	1.1630967	1.53558362	1.70936622	0.55478469
MAR	0.64813253	0.66110121	0	1.31437776	0.72551698	1.51539373	1.34160297	0.65894529	0.63764808	1.08017998	0.82277357	0.4788234	1.71682035	0.85324907	1.33223624	1.5892731	0.82681272
MIS	1.73490411	1.834922	1.31437776	0	1.99397534	2.68031711	2.40953423	1.82911055	1.83272412	2.30715598	1.86374998	1.45339408	2.40023031	1.72223619	1.65104302	2.55286102	2.05669161
OMS	0.85794801	0.425087	0.72551698	1.99397534	0	1.30520906	1.27064585	0.56459076	0.29047542	0.43522457	0.72664967	0.93024561	1.91485394	0.88592148	1.80919105	1.30109726	0.65820457
PIT	1.37987904	1.57717022	1.51539373	2.68031711	1.30520906	0	0.39523867	1.11137498	1.54159399	1.50705788	1.25119531	1.53632149	1.09606192	1.48640295	2.10352957	1.35886466	1.21086878
POD	1.35637973	1.55564256	1.34160297	2.40953423	1.27064585	0.39523867	0	0.91659549	1.49604436	1.57139995	1.02156845	1.44344152	0.79005323	1.22530818	2.04711074	1.17023822	1.27205818
PON	1.01444398	0.87558733	0.65894529	1.82911055	0.56459076	1.11137498	0.91659549	0	0.74552201	0.98907546	0.25427483	1.00456895	1.44467047	0.46231594	1.86624112	0.95220944	0.95408403
RAD	0.77971629	0.18871558	0.63764808	1.83272412	0.29047542	1.54159399	1.49604436	0.74552201	0	0.47588702	0.90190325	0.80992657	2.08834549	1.00311491	1.67246039	1.5438496	0.66046702
STR	1.0236477	0.50387017	1.08017998	2.30715598	0.43522457	1.50705788	1.57139995	0.98907546	0.47588702	0	1.13520733	1.15996335	2.27460396	1.29239617	1.95549733	1.59112585	0.72355142
TAN	1.25699621	1.056937	0.82277357	1.86374998	0.72664967	1.25119531	1.02156845	0.25427483	0.90190325	1.13520733	0	1.22255134	1.4922911	0.25455078	2.08309136	0.77034249	1.19915275
TRD	0.28162653	0.71544228	0.4788234	1.45339408	0.93024561	1.53632149	1.44344152	1.00456895	0.80992657	1.15996335	1.22255134	0	1.81405901	1.3031753	0.90213563	1.95476502	0.64030203
VAL	1.81505397	2.14151739	1.71682035	2.40023031	1.91485394	1.09606192	0.79005323	1.44467047	2.08834549	2.27460396	1.4922911	1.81405901	0	1.59473033	2.20923989	1.6141781	1.87591792
VOD	1.38573205	1.1630967	0.85324907	1.72223619	0.88592148	1.48640295	1.22530818	0.46231594	1.00311491	1.29239617	0.25455078	1.3031753	1.59473033	0	2.15025248	0.85069008	1.3763287
VRE	0.97358015	1.53558362	1.33223624	1.65104302	1.80919105	2.10352957	2.04711074	1.86624112	1.67246039	1.95549733	2.08309136	0.90213563	2.20923989	2.15025248	0	2.79495687	1.32449484
ZAG	1.93828323	1.70936622	1.5892731	2.55286102	1.30109726	1.35886466	1.17023822	0.95220944	1.5438496	1.59112585	0.77034249	1.95476502	1.6141781	0.85069008	2.79495687	0	1.78997422
ZAL	0.39258305	0.55478469	0.82681272	2.05669161	0.65820457	1.21086878	1.27205818	0.95408403	0.66046702	0.72355142	1.19915275	0.64030203	1.87591792	1.3763287	1.32449484	1.78997422	0

j) Coenotic niche based on bryophytes

Locality	BAL	BAZ	CAV	DOS	EBE	FAL	FEN	GAV	GNI	GOS	HIR	HOD	HUS	JAK	KAM	KOL	LAG	LOI	LOR	LYS	MAR	MIS	OMS	PIT	POD	PON	RAD	STR	TAN	TRD	VOD	ZAG	ZAL
BAL	0	1.19241632	2.86740752	1.90357823	2.59367484	2.95712574	1.91941318	1.59015756	1.91838308	2.57351314	2.44533515	2.69617829	3.21314641	3.4594652	1.48223058	2.23075908	2.14942281	1.78929088	2.82323501	2.13348389	3.0076546	1.86339267	2.08477171	2.42934574	2.71318747	2.31689421	2.82928729	2.33010134	2.00608893	1.24765284	2.06920467	1.66424137	2.69425715
BAZ	1.19241632	0	2.22237075	1.42539236	1.91633713	2.99154606	1.1245159	0.6992998	1.96616389	1.77868088	2.1642998	2.15132966	2.27913907	3.03405392	1.42278296	1.73356245	1.60892959	1.60531526	2.41965854	1.68734602	2.07912647	1.63062615	1.36144168	1.48443435	1.66885418	1.65386206	2.13425742	1.68684761	1.17159313	1.40375702	1.35993786	1.10788237	2.10287767
CAV	2.86740752	2.22237075	0	1.24170607	0.33858324	1.73309875	1.11069141	1.78860865	1.54128659	0.68465569	0.84170277	0.26932631	1.13977768	0.96687986	1.71162728	0.77620433	0.86220177	1.63091084	0.62519209	0.94205048	0.97625899	1.18791705	0.91919794	1.0293402	1.30506755	1.10967892	0.76768133	1.08195605	1.07225408	1.83222463	1.17912881	1.22525453	0.71113421
DOS	1.90357823	1.42539236	1.24170607	0	1.101408	1.76802333	0.85556344	0.87278941	1.39000095	1.29527545	1.0448225	1.1176138	1.96734779	1.78071544	0.60554751	1.08714638	0.38285962	1.57277153	1.20018679	0.36942998	1.33702834	0.55372391	0.95569323	1.27326812	1.62931182	0.47467271	0.9539967	1.48656507	0.72517838	1.23784165	0.37931635	0.75075906	1.52738271
EBE	2.59367484	1.91633713	0.33858324	1.101408	0	1.85394807	0.79377537	1.53941074	1.38951108	0.40153879	0.85693429	0.37472242	1.0349701	1.27205665	1.55564986	0.52356517	0.73755425	1.36230348	0.81121527	0.85261101	0.97601002	1.05482065	0.58633684	0.745739	1.07496836	1.03856319	0.86923983	0.78597767	0.79323401	1.59594434	1.02090204	0.93245235	0.55074108
FAL	2.95712574	2.99154606	1.73309875	1.76802333	1.85394807	0	2.22305572	2.60856563	1.40991201	2.22568831	1.0308042	1.50882096	2.83688391	1.25785367	1.71829267	1.73121812	1.63487448	2.12671287	1.11477728	1.49127367	2.49268949	1.37099017	2.10252483	2.52671112	2.90436519	1.94950231	1.87201364	2.27161541	2.21640687	1.89071827	2.05940184	2.00424282	1.97212664
FEN	1.91941318	1.1245159	1.11069141	0.85556344	0.79377537	2.22305572	0	0.8595872	1.36610076	0.69640852	1.23602362	1.06270691	1.34741159	1.98212857	1.20600947	0.71428513	0.74302838	1.11032588	1.40402189	0.88182925	1.21430338	0.96139612	0.26212354	0.56239227	0.94136695	0.97144549	1.22399612	0.77441355	0.2404298	1.20266802	0.73532829	0.37380879	1.04071769
GAV	1.59015756	0.6992998	1.78860865	0.87278941	1.53941074	2.60856563	0.8595872	0	1.87997182	1.49793709	1.79537442	1.73832517	2.03451465	2.54741528	1.07845736	1.5030356	1.09800563	1.72076366	1.96755283	1.18314511	1.53151077	1.26688483	1.11541334	1.22141085	1.45029079	1.0037172	1.52248007	1.61543246	0.75998764	1.45460979	0.73101697	0.9064555	1.88915265
GNI	1.91838308	1.96616389	1.54128659	1.39000095	1.38951108	1.40991201	1.36610076	1.87997182	0	1.55519514	0.88189811	1.2917435	2.25387615	1.85859995	1.23428241	0.91380405	1.27732147	0.76783371	1.3106423	1.21663446	2.26826784	0.86394143	1.25695893	1.7767015	2.16984381	1.75487354	1.90545165	1.27994463	1.50241175	0.67990859	1.64718195	1.05483596	1.29424552
GOS	2.57351314	1.77868088	0.68465569	1.29527545	0.40153879	2.22568831	0.69640852	1.49793709	1.55519514	0	1.2068601	0.76797063	0.74795215	1.63845562	1.73916546	0.64521265	0.98993597	1.31428454	1.20848448	1.1338673	1.00248341	1.30147635	0.49438464	0.43490686	0.73271605	1.23308025	1.13776559	0.56579495	0.75038835	1.66943863	1.14196477	0.9440787	0.55079566
HIR	2.44533515	2.1642998	0.84170277	1.0448225	0.85693429	1.0308042	1.23602362	1.79537442	0.88189811	1.2068601	0	0.57548151	1.86942755	1.03927196	1.22724599	0.72279045	0.77175142	1.3378961	0.43950818	0.69767131	1.6387382	0.65047905	1.08473625	1.50438388	1.88701312	1.21121455	1.15503839	1.27867305	1.25185755	1.2960897	1.24612852	1.08128985	1.02164614
HOD	2.69617829	2.15132966	0.26932631	1.1176138	0.37472242	1.50882096	1.06270691	1.73832517	1.2917435	0.76797063	0.57548151	0	1.34009367	0.93561299	1.52266623	0.62023094	0.73791188	1.46393048	0.44188656	0.78336889	1.16751489	0.96750071	0.86758862	1.11451433	1.44444664	1.08293965	0.84063308	1.04617596	1.04807058	1.61577146	1.13564137	1.09444886	0.69158951
HUS	3.21314641	2.27913907	1.13977768	1.96734779	1.0349701	2.83688391	1.34741159	2.03451465	2.25387615	0.74795215	1.86942755	1.34009367	0	1.98675989	2.46083167	1.35821899	1.6698577	1.92215803	1.75594982	1.82152996	1.12578094	2.03896318	1.20448243	0.82677817	0.6900606	1.78482677	1.5541618	1.08707868	1.36797233	2.38670992	1.73266021	1.65572951	1.02895848
JAK	3.4594652	3.03405392	0.96687986	1.78071544	1.27205665	1.25785367	1.98212857	2.54741528	1.85859995	1.63845562	1.03927196	0.93561299	1.98675989	0	2.12090554	1.50086752	1.46378324	2.25545476	0.64059208	1.42908378	1.70441677	1.60496302	1.8019927	1.99365696	2.26023636	1.67300018	1.24105599	1.92104741	1.93022935	2.33440861	1.85202871	1.97787429	1.45104694
KAM	1.48223058	1.42278296	1.71162728	0.60554751	1.55564986	1.71829267	1.20600947	1.07845736	1.23428241	1.73916546	1.22724599	1.52266623	2.46083167	2.12090554	0	1.35014606	0.88832317	1.5342219	1.52193576	0.79620756	1.93934602	0.59288382	1.3121693	1.73087266	2.10544269	1.06308329	1.5251697	1.75897471	1.16934728	0.92923859	0.94793227	0.92890261	1.85894792
KOL	2.23075908	1.73356245	0.77620433	1.08714638	0.52356517	1.73121812	0.71428513	1.5030356	0.91380405	0.64521265	0.72279045	0.62023094	1.35821899	1.50086752	1.35014606	0	0.79542355	0.85967413	0.93754302	0.86063522	1.44373791	0.83350599	0.4861363	0.9293204	1.3123163	1.2414133	1.26172321	0.56446816	0.83902297	1.13878348	1.13750655	0.6602868	0.5188799
LAG	2.14942281	1.60892959	0.86220177	0.38285962	0.73755425	1.63487448	0.74302838	1.09800563	1.27732147	0.98993597	0.77175142	0.73791188	1.6698577	1.46378324	0.88832317	0.79542355	0	1.45080248	0.8748322	0.17247	1.13188316	0.5249215	0.74141946	1.06864219	1.44193999	0.49290848	0.72827384	1.23374822	0.63260825	1.29219131	0.47475103	0.70169731	1.18596936
LOI	1.78929088	1.60531526	1.63091084	1.57277153	1.36230348	2.12671287	1.11032588	1.72076366	0.76783371	1.31428454	1.3378961	1.46393048	1.92215803	2.25545476	1.5342219	0.85967413	1.45080248	0	1.69347291	1.4801269	2.20268727	1.22930688	1.01320171	1.44217077	1.77259983	1.88761399	2.06812066	0.83528799	1.33149055	0.77346064	1.69101104	0.89900728	1.13518806
LOR	2.82323501	2.41965854	0.62519209	1.20018679	0.81121527	1.11477728	1.40402189	1.96755283	1.3106423	1.20848448	0.43950818	0.44188656	1.75594982	0.64059208	1.52193576	0.93754302	0.8748322	1.69347291	0	0.83305035	1.4442979	0.97762608	1.23979507	1.53442535	1.86971703	1.19512297	0.93341123	1.43732299	1.36868175	1.71438672	1.31442873	1.35346603	1.06510291
LYS	2.13348389	1.68734602	0.94205048	0.36942998	0.85261101	1.49127367	0.88182925	1.18314511	1.21663446	1.1338673	0.69767131	0.78336889	1.82152996	1.42908378	0.79620756	0.86063522	0.17247	1.4801269	0.83305035	0	1.27304672	0.41603719	0.87567613	1.23512	1.61251501	0.56266805	0.79719711	1.342265	0.78778232	1.25473995	0.57967917	0.77774828	1.27218658
MAR	3.0076546	2.07912647	0.97625899	1.33702834	0.97601002	2.49268949	1.21430338	1.53151077	2.26826784	1.00248341	1.6387382	1.16751489	1.12578094	1.70441677	1.93934602	1.44373791	1.13188316	2.20268727	1.4442979	1.27304672	0	1.65197072	1.20207593	0.93868668	0.99036774	0.94176815	0.68070136	1.55410043	1.03765029	2.30336501	1.02812751	1.50029145	1.44943006
MIS	1.86339267	1.63062615	1.18791705	0.55372391	1.05482065	1.37099017	0.96139612	1.26688483	0.86394143	1.30147635	0.65047905	0.96750071	2.03896318	1.60496302	0.59288382	0.83350599	0.5249215	1.22930688	0.97762608	0.41603719	1.65197072	0	0.9497691	1.42151311	1.824459	0.93736637	1.20598162	1.33365945	0.95903246	0.88539339	0.86905168	0.69698613	1.32382861
OMS	2.08477171	1.36144168	0.91919794	0.95569323	0.58633684	2.10252483	0.26212354	1.11541334	1.25695893	0.49438464	1.08473625	0.86758862	1.20448243	1.8019927	1.3121693	0.4861363	0.74141946	1.01320171	1.23979507	0.87567613	1.20207593	0.9497691	0	0.52977883	0.93393286	1.05243759	1.1888935	0.56415494	0.41824364	1.22471062	0.87098332	0.45381214	0.77915457
PIT	2.42934574	1.48443435	1.0293402	1.27326812	0.745739	2.52671112	0.56239227	1.22141085	1.7767015	0.43490686	1.50438388	1.11451433	0.82677817	1.99365696	1.73087266	0.9293204	1.06864219	1.44217077	1.53442535	1.23512	0.93868668	1.42151311	0.52977883	0	0.40884414	1.18619444	1.22819515	0.76243933	0.58062109	1.72469491	1.03233794	0.92000274	0.96202398
POD	2.71318747	1.66885418	1.30506755	1.62931182	1.07496836	2.90436519	0.94136695	1.45029079	2.16984381	0.73271605	1.88701312	1.44444664	0.6900606	2.26023636	2.10544269	1.3123163	1.44193999	1.77259983	1.86971703	1.61251501	0.99036774	1.824459	0.93393286	0.40884414	0	1.47444697	1.46657365	1.04459415	0.93697386	2.10328166	1.33944742	1.30753249	1.23832495
PON	2.31689421	1.65386206	1.10967892	0.47467271	1.03856319	1.94950231	0.97144549	1.0037172	1.75487354	1.23308025	1.21121455	1.08293965	1.78482677	1.67300018	1.06308329	1.2414133	0.49290848	1.88761399	1.19512297	0.56266805	0.94176815	0.93736637	1.05243759	1.18619444	1.47444697	0	0.5812009	1.59085282	0.75962102	1.67816416	0.29874164	1.03982464	1.56124612
RAD	2.82928729	2.13425742	0.76768133	0.9539967	0.86923983	1.87201364	1.22399612	1.52248007	1.90545165	1.13776559	1.15503839	0.84063308	1.5541618	1.24105599	1.5251697	1.26172321	0.72827384	2.06812066	0.93341123	0.79719711	0.68070136	1.20598162	1.1888935	1.22819515	1.46657365	0.5812009	0	1.6097537	1.04396966	2.01586621	0.81204117	1.35279352	1.40837112
STR	2.33010134	1.68684761	1.08195605	1.48656507	0.78597767	2.27161541	0.77441355	1.61543246	1.27994463	0.56579495	1.27867305	1.04617596	1.08707868	1.92104741	1.75897471	0.56446816	1.23374822	0.83528799	1.43732299	1.342265	1.55410043	1.33365945	0.56415494	0.76243933	1.04459415	1.59085282	1.6097537	0	0.96669357	1.38698437	1.43239421	0.87290725	0.52639914
TAN	2.00608893	1.17159313	1.07225408	0.72517838	0.79323401	2.21640687	0.2404298	0.75998764	1.50241175	0.75038835	1.25185755	1.04807058	1.36797233	1.93022935	1.16934728	0.83902297	0.63260825	1.33149055	1.36868175	0.78778232	1.03765029	0.95903246	0.41824364	0.58062109	0.93697386	0.75962102	1.04396966	0.96669357	0	1.34206859	0.52498409	0.51141615	1.15339888
TRD	1.24765284	1.40375702	1.83222463	1.23784165	1.59594434	1.89071827	1.20266802	1.45460979	0.67990859	1.66943863	1.2960897	1.61577146	2.38670992	2.33440861	0.92923859	1.13878348	1.29219131	0.77346064	1.71438672	1.25473995	2.30336501	0.88539339	1.22471062	1.72469491	2.10328166	1.67816416	2.01586621	1.38698437	1.34206859	0	1.48416107	0.8383547	1.61076707
VOD	2.06920467	1.35993786	1.17912881	0.37931635	1.02090204	2.05940184	0.73532829	0.73101697	1.64718195	1.14196477	1.24612852	1.13564137	1.73266021	1.85202871	0.94793227	1.13750655	0.47475103	1.69101104	1.31442873	0.57967917	1.02812751	0.86905168	0.87098332	1.03233794	1.33944742	0.29874164	0.81204117	1.43239421	0.52498409	1.48416107	0	0.80630794	1.49778418
ZAG	1.66424137	1.10788237	1.22525453	0.75075906	0.93245235	2.00424282	0.37380879	0.9064555	1.05483596	0.9440787	1.08128985	1.09444886	1.65572951	1.97787429	0.92890261	0.6602868	0.70169731	0.89900728	1.35346603	0.77774828	1.50029145	0.69698613	0.45381214	0.92000274	1.30753249	1.03982464	1.35279352	0.87290725	0.51141615	0.8383547	0.80630794	0	1.12027711
ZAL	2.69425715	2.10287767	0.71113421	1.52738271	0.55074108	1.97212664	1.04071769	1.88915265	1.29424552	0.55079566	1.02164614	0.69158951	1.02895848	1.45104694	1.85894792	0.5188799	1.18596936	1.13518806	1.06510291	1.27218658	1.44943006	1.32382861	0.77915457	0.96202398	1.23832495	1.56124612	1.40837112	0.52639914	1.15339888	1.61076707	1.49778418	1.12027711	0
