# Supplementary material for: Ecological differentiation, speciation, and rarity: How do they match in Tephroseris longifolia agg. (Asteraceae)?
Source: Ecol Evol. 2018 Jan 31;8(5):2453–70. doi: 10.1002/ece3.3770 (PMC5838062; doi:10.1002/ece3.3770)
Supplement: Supplementary file 2 [file ECE3-8-2453-s002.pdf]

Supporting information to the manuscript of Janišová et al., Ecological differentiation, speciation and rarity – how do they match in *Tephroseris longifolia* agg. (Asteraceae)?

Appendix S2 Correlation (Pearson correlation coefficients) between morphological characters or relative DNA content and climatic variables for particular populations of the *T. longifolia* agg. Marked correlations are significant at p < 0.01 (positive correlations in red and negative correlations in blue).

		Altitude	AMT	ETR	TX30	SU	TNX0	TN10	PTGS	SDII	CDD	RR1	T_MIN	GSS5
**Morphological characters**														
SLI1	indument of lower part of stem - sparsely to moderately hairy	-0.02	0.03	-0.01	-0.11	-0.03	-0.12	0.00	0.12	0.13	-0.24	0.21	0.04	0.05
SLI2	indument of lower part of stem - densely hairy to arachnoid	0.15	-0.08	-0.16	0.13	-0.04	0.16	-0.04	-0.10	-0.06	0.34	-0.31	0.09	0.03
SUI1	indument of upper part of stem - sparsely to moderately hairy	-0.21	0.24	0.12	0.01	0.18	-0.28	-0.16	0.04	0.07	-0.29	0.17	0.10	-0.20
SUI2	indument of upper part of stem - densely hairy to arachnoid	0.21	-0.24	-0.13	-0.01	-0.18	0.28	0.16	-0.04	-0.06	0.30	-0.18	-0.10	0.20
LLUSI1	indument of upper surface of lower stem leaf - sparsely to moderately hairy	0.02	0.06	-0.18	-0.18	-0.14	-0.21	-0.16	0.21	0.25	-0.17	0.17	0.24	0.02
LLUSI2	indument of upper surface of lower stem leaf - densely hairy to arachnoid	0.26	-0.24	-0.11	0.00	-0.12	0.34	0.19	-0.11	-0.13	0.28	-0.14	-0.10	0.22
LLLSI1	indument of lower surface of lower stem leaf - sparsely to moderately hairy	0.06	-0.12	0.00	-0.08	-0.16	0.09	0.09	0.24	0.18	-0.12	0.14	-0.11	0.13
LLLSI2	indument of lower surface of lower stem leaf - densely hairy to arachnoid	0.37	-0.14	-0.38	-0.11	-0.15	0.18	-0.03	-0.18	-0.10	0.35	-0.21	0.27	0.20
LMLSI1	indument of lower surface of middle stem leaf - sparsely to moderately hairy	-0.09	0.10	0.04	-0.10	-0.04	-0.13	-0.07	0.29	0.23	-0.42	0.34	0.04	-0.03
LMLSI2	indument of lower surface of middle stem leaf - densely hairy to arachnoid	0.44	-0.29	-0.39	-0.03	-0.21	0.33	0.10	-0.22	-0.13	0.59	-0.38	0.14	0.30
BI1	indument of involucral bracts - sparsely to moderately hairy	-0.43	0.33	0.27	-0.08	0.20	-0.45	-0.21	0.36	0.35	-0.49	0.31	0.02	-0.33
BI2	indument of involucral bracts - densely hairy to arachnoid	0.56	-0.44	-0.34	-0.08	-0.35	0.52	0.27	-0.31	-0.30	0.52	-0.26	-0.04	0.46
NPC	number of primary capitula	0.06	-0.08	-0.05	0.01	-0.07	0.08	0.11	-0.07	-0.06	-0.02	0.01	-0.06	0.05
NSC	number of secondary capitula	-0.23	0.26	0.23	0.33	0.33	-0.12	-0.07	-0.33	-0.31	-0.19	-0.09	-0.06	-0.28
NL	number of leaves	0.03	-0.02	0.14	0.12	-0.02	-0.03	0.07	-0.27	-0.20	0.07	-0.25	-0.19	0.00
PH	plant height	-0.19	0.27	0.12	0.25	0.29	-0.18	-0.14	-0.34	-0.28	-0.14	-0.13	0.09	-0.26
SLUB	stem length up to branching	-0.16	0.25	0.11	0.24	0.27	-0.17	-0.12	-0.38	-0.32	-0.09	-0.17	0.08	-0.23
LLNT	number of teeth of lower stem leaf	0.09	-0.20	-0.23	-0.53	-0.38	0.03	0.03	0.47	0.42	-0.13	0.44	0.10	0.21
LMNT	number of teeth of middle stem leaf	0.09	-0.16	-0.32	-0.59	-0.39	-0.04	0.00	0.52	0.45	-0.27	0.61	0.22	0.20
LLL	length of blade of lower stem leaf	0.00	0.02	0.37	0.46	0.38	0.26	0.24	-0.61	-0.65	0.06	-0.25	-0.41	-0.04
LLW	width of blade of lower stem leaf	-0.26	0.30	-0.13	-0.23	0.04	-0.38	-0.36	0.19	0.20	-0.31	0.25	0.42	-0.26
LLD	distance of widest part of blade of lower stem leaf	-0.02	0.02	0.43	0.47	0.38	0.25	0.26	-0.57	-0.62	0.03	-0.24	-0.48	-0.04
LLPL	length of peduncules of lower stem leaf	-0.08	0.18	-0.27	-0.31	-0.16	-0.37	-0.37	0.12	0.22	0.00	0.01	0.47	-0.14
LLBA	angle of base of blade of lower stem leaf	0.07	-0.12	-0.12	-0.15	-0.04	0.23	0.18	0.27	0.13	-0.20	0.44	-0.01	0.17
LLTD	depth of maximum tooth of lower stem leaf	0.01	0.02	-0.31	-0.56	-0.31	-0.23	-0.19	0.43	0.44	-0.20	0.37	0.37	0.04
LML	length of blade of middle stem leaf	0.11	0.13	-0.27	-0.12	-0.04	-0.21	-0.27	-0.16	-0.07	0.02	0.00	0.40	-0.04
LMW	width of blade of middle stem leaf	-0.04	0.20	-0.28	-0.30	-0.08	-0.31	-0.32	0.18	0.20	-0.28	0.32	0.48	-0.09
LMD	distance of middle part of blade of lower stem leaf	0.04	0.15	-0.11	-0.21	-0.06	-0.24	-0.26	-0.06	-0.02	-0.12	0.10	0.27	-0.06
LMBA	angle of base of blade of middle stem leaf	-0.25	0.14	0.13	0.25	0.33	-0.03	0.02	-0.15	-0.18	-0.05	-0.01	-0.03	-0.20
LMTW	width of maximum tooth of middle stem leaf	0.09	0.04	-0.33	-0.42	-0.19	-0.18	-0.19	0.30	0.32	-0.19	0.35	0.40	0.05
LMTD	depth of maximum tooth of middle stem leaf	0.18	-0.06	-0.42	-0.52	-0.34	-0.09	-0.12	0.33	0.32	-0.21	0.44	0.40	0.15
CTD	terminal capitulum diameter	-0.45	0.59	0.18	0.13	0.41	-0.56	-0.52	0.03	0.06	-0.41	0.09	0.32	-0.50
CTDD	terminal capitulum disc diameter	-0.24	0.13	0.31	0.35	0.28	0.03	0.04	-0.29	-0.32	-0.09	-0.14	-0.21	-0.20
CTIL	terminal capitulum involucre length	0.37	-0.24	-0.23	-0.04	-0.07	0.37	0.29	-0.29	-0.26	0.23	-0.09	0.01	0.27
CTIW	terminal capitulum involucre width	0.53	-0.51	-0.38	-0.53	-0.50	0.38	0.35	0.07	0.11	0.32	0.12	-0.02	0.55
PTL	length of pedicel of terminal capitulum	-0.49	0.39	0.47	0.34	0.44	-0.21	-0.18	-0.04	-0.10	-0.46	0.12	-0.15	-0.48
CLD	lateral capitulum diameter	-0.42	0.54	0.18	0.12	0.39	-0.50	-0.46	0.00	0.02	-0.43	0.12	0.28	-0.47
CLDD	lateral capitulum disc diameter	-0.23	0.12	0.22	0.22	0.19	-0.02	0.01	-0.22	-0.24	-0.12	-0.05	-0.12	-0.19
CLIL	lateral capitulum involucre length	0.22	-0.10	0.05	0.15	0.12	0.32	0.27	-0.50	-0.50	0.08	-0.12	-0.16	0.13
CLIW	lateral capitulum involucre width	0.44	-0.52	-0.14	-0.49	-0.47	0.44	0.45	0.05	-0.02	0.11	0.31	-0.29	0.53
PLL	length of pedicel of lateral capitulum	-0.34	0.36	0.24	0.23	0.37	-0.22	-0.17	0.01	-0.01	-0.47	0.19	0.05	-0.39
CTIL/CTIW		0.05	0.09	0.00	0.37	0.31	0.17	0.08	-0.44	-0.43	0.03	-0.20	0.04	-0.08
CLIL/CLIW		-0.05	0.23	0.16	0.50	0.45	0.06	-0.01	-0.59	-0.55	0.01	-0.33	0.00	-0.21
SLUB/SL		0.10	-0.06	0.03	-0.01	-0.03	0.00	0.04	-0.28	-0.23	0.25	-0.23	-0.06	0.12
LLW/LLL		-0.22	0.18	-0.25	-0.41	-0.18	-0.40	-0.36	0.52	0.54	-0.27	0.33	0.47	-0.17
LLD/LLL		0.04	-0.01	0.40	0.40	0.30	0.24	0.26	-0.46	-0.50	0.04	-0.22	-0.47	0.01
LLPL/LLL		-0.13	0.14	-0.28	-0.40	-0.24	-0.39	-0.36	0.38	0.44	-0.10	0.17	0.46	-0.14
LMW/LML		-0.20	0.19	-0.14	-0.34	-0.09	-0.32	-0.26	0.43	0.38	-0.48	0.52	0.34	-0.13
LMD/LML		-0.16	0.15	0.25	-0.22	-0.04	-0.23	-0.14	0.17	0.11	-0.33	0.24	-0.09	-0.10
**Relative DNA content** expressed as the ratio of G1 peak of standard (*Bellis perennis* L.) and G1 peak of the sample														
RSS	Relative DNA content	0.60	-0.70	-0.54	-0.19	-0.57	0.49	0.39	0.02	0.12	0.81	-0.38	-0.01	0.61
